# Cell Compartment-Specific Folding of Ty1 Long Terminal Repeat Retrotransposon RNA Genome

**DOI:** 10.3390/v14092007

**Published:** 2022-09-10

**Authors:** Małgorzata Zawadzka, Angelika Andrzejewska-Romanowska, Julita Gumna, David J. Garfinkel, Katarzyna Pachulska-Wieczorek

**Affiliations:** 1 Department of Structure and Function of Retrotransposons, Institute of Bioorganic Chemistry, Polish Academy of Sciences, Noskowskiego 12/14, 61-704 Poznan, Poland; 2 Department of Biochemistry and Molecular Biology, University of Georgia, Athens, GA 30602, USA

**Keywords:** RNA genome, RNA structure, cell compartment-specific folding, LTR-retrotransposon, gRNA dimerization, gRNA cyclization, tRNA annealing, Ty1, Gag

## Abstract

The structural transitions RNAs undergo during trafficking are not well understood. Here, we used the well-developed yeast Ty1 retrotransposon to provide the first structural model of genome (g) RNA in the nucleus from a retrovirus-like transposon. Through a detailed comparison of nuclear Ty1 gRNA structure with those established in the cytoplasm, virus-like particles (VLPs), and those synthesized in vitro, we detected Ty1 gRNA structural alterations that occur during retrotransposition. Full-length Ty1 gRNA serves as the mRNA for Gag and Gag-Pol proteins and as the genome that is reverse transcribed within VLPs. We show that about 60% of base pairs predicted for the nuclear Ty1 gRNA appear in the cytoplasm, and active translation does not account for such structural differences. Most of the shared base pairs are represented by short-range interactions, whereas the long-distance pairings seem unique for each compartment. Highly structured motifs tend to be preserved after nuclear export of Ty1 gRNA. In addition, our study highlights the important role of Ty1 Gag in mediating critical RNA–RNA interactions required for retrotransposition.

## 1. Introduction

Eukaryotic genomes are rich in transposable elements [1]. One prevalent group contains the long terminal repeat (LTR)-retrotransposon families Ty1/Copia (*Pseudoviridae*), Ty3/Gypsy (*Metaviridae*), and BEL/Pao (*Belpaoviridae*) [2]. Their genomic organization resembles that of simple retroviruses, but most lack a functional envelope gene required for extracellular transmission [3]. One of the best characterized LTR-retrotransposons is Ty1, which is present in many *Saccharomyces cerevisiae* strains [4,5] and propagates via an RNA intermediate [6,7]. The integrated Ty1 element is transcribed by the RNA polymerase II, producing a gRNA that comprises two partially overlapped *GAG* and *POL* ORFs flanked by untranslated regions [8]. After export to the cytoplasm, Ty1 gRNA fulfills a dual role, serving as the template for Gag and Gag–Pol protein synthesis and as a genome that is packaged in a dimeric form into VLPs where reverse transcription occurs. In VLPs, Gag and Gag–Pol undergo proteolytic maturation, gRNA is reversed transcribed using tRNA_i_^Met^ as a primer, and the resulting cDNA is imported into the nucleus and integrated into host DNA, usually upstream of the genes transcribed by RNA polymerase III [9,10,11].

Ty1 Gag is the structural component of VLPs and mediates RNA transactions during the process of Ty1 retrotransposition. Direct interactions with Gag are needed for Ty1 RNA trafficking to preassembly sites termed retrosomes, as well as VLP assembly [12]. Gag also enhances the stability and nuclear export of the Ty1 gRNA [13]. Like retroviral Gag polyproteins, Ty1 Gag displays nucleic acid chaperone activity in vitro and facilitates RNA–RNA interactions such as dimerization and cyclization of Ty1 gRNA, annealing of tRNA_i_^Met^, and initiation of reverse transcription [14,15,16,17].

Ty1 gRNA is 5.6-kb long, is not spliced, and ~15% of the transcripts undergo polyadenylation [18]. However, Ty1 transcripts are highly abundant and can comprise ~0.8% of total RNA, in part because Ty1 RNA is more stable than other mRNAs [19,20]. Like retroviruses, the Ty1 gRNA 5′ and 3′ termini contain cis-acting sequences directly involved in retrotransposition [15,16,21,22,23]. Recently, we showed that cytoplasmic Ty1 gRNA adopts a significantly different and more heterogeneous structure than when synthesized under in vitro conditions, but it retains specific well-structured regions containing functional cis-acting sequences [24]. We also provided evidence that critical RNA–RNA interactions required for retrotransposition occur prior to VLP assembly. 

Here, we used SHAPE (Selective 2′ Hydroxyl Acylation analyzed by Primer Extension) mapping to explore the Ty1 gRNA structure inside the nucleus of living yeast cells. Using complementary and synergistic experimental and bioinformatics approaches, we compared structural elements of nuclear Ty1 gRNA with that in the cytoplasm, VLPs, and in vitro. These analyses reveal cell compartment-specific structural transitions that Ty1 gRNA undergoes during the retrotransposon replication cycle. We also demonstrate the importance of Ty1 Gag in functionally important RNA–RNA interactions. Together, our work provides the first RNA structure model for a retroviral-like nuclear gRNA and the structural rearrangements that occur during its cellular journey, further increasing our understanding of RNA folding.

## 2. Materials and Methods

### 2.1. Yeast Strains, Media, and Growth Conditions

Strains DG3408 (MAC483) and DG3412 (MAC520) [13] are derived from Ty1-less *S. paradoxus* strain DG1768 (MATα his3-Δ200hisG ura3 gal3 Spo^-^) and contain multicopy plasmids pGTy1fs (pBDG1112, pGTy1gag-ATGfs) and pGTy1 (pBDG202, wild type), respectively, under the control of GAL1 promoter [25,26,27]. DG3408 was grown overnight to OD600 of 1.0 in SC-Ura 2% raffinose broth at 30 °C with shaking at 250 rpm. The saturated yeast culture was centrifuged at 7500× *g* for 5 min, the pellet was washed once in PBS buffer, and then it was resuspended in SC-Ura 2% galactose broth. The culture was grown for 2–4 h at 22 °C with 250 rpm shaking to induce “Ty1fs” expression from the GAL1 promoter. The *S. cerevisiae* reference strain BY4742 (MATα his3Δ1 leu2Δ0 lys2Δ0 ura3Δ0) [28,29] was used for testing the effectivity of RNA modification by NMIA, and NAI in the nucleus was grown in conditions similar to those mentioned above using SC-complete broth instead of SC-Ura.

### 2.2. RNA Modification

A total of 100 mL of galactose-induced yeast culture (DG3408 or BY4742) was centrifuged at 7500× *g* for 5 min, the cell pellet was washed with PBS, and then it was resuspended in 720 µL of fresh PBS. Samples were divided into two equal parts and treated with either 40 µL of SHAPE reagent in DMSO [final concentration: 10 mM NMIA, 10 mM NAI; (+)] or 40 µL DMSO alone (-). The modification reactions were carried out at 37 °C for 15 min. RNA modification reactions with NAI were additionally quenched by adding 1 M DTT at a 1:1 ratio to the reaction volume. Cells were collected by centrifugation at 8000× *g* for 5 min.

### 2.3. Isolation of Total RNA

RNA isolation was performed as previously described [24]. Briefly, the cell pellet was resuspended in 1 ml of lysis buffer (10 mM Tris-HCl, pH 8.5, 5 mM EDTA, 2% SDS, 2% 2-mercaptoethanol) and incubated at 83 °C for 20 min with constant shaking at 450 rpm. Then, samples were centrifuged at 12,000× *g* for 5 min, supernatants were transferred to fresh tubes, and RNA was extracted twice with phenol (pH 8.0) and twice with acid phenol:chloroform (pH 4.5). RNA was recovered by LiCl precipitation overnight at −20 °C, pellets were washed twice with 70% ethanol, and then they were resuspended in an appropriate amount of water.

### 2.4. Primer Extension and Data Processing

The optimal amount of SHAPE modified (+) or control (−) RNA sample was combined with 1 µL of 10 µM fluorescently labeled primer [Cy5 (+) or Cy5.5(−)] and 1 µL 2 mM EDTA pH 8.0, 3.84 µL of 5 M betaine and water to a final volume of 12 µL. Primer-template solutions were initially incubated at 95 °C for 3 min, 37 °C for 10 min, and 55 °C for 2 min. A total of 8 µL of reverse transcriptase mix (SuperSript III, Invitrogen, Waltham, MA, USA) was added to each reaction tube, followed by an additional 90 min incubation at 50 °C. Sequencing ladders were prepared using labeled primer: WellRed D2 (ddA) or IRD-800 (ddT) and a Thermo Sequenase Cycle Sequencing kit (Applied Biosystems, Waltham, MA, USA) as recommended by the manufacturer. Reverse transcribed samples and sequencing ladders were purified using ZR DNA Sequencing Clean-Up Kit (Zymo Research, Irvine, CA, USA) and analyzed on a GenomeLab GeXP Analysis System (Beckman-Coulter, Pasadena, CA, USA). Raw electropherograms were processed using ShapeFinder software [30] and normalized using RNAthor software [31]. Data for each read were obtained from at least two independent experiments. All primers are listed in Table S1. 

### 2.5. RNA Secondary Structure Modeling and Analysis

A SHAPE-derived minimum free energy (MFE) secondary structure model for nuclear Ty1 gRNA was predicted using an updated version of SuperFold (v1.1) [32]. The previously obtained SHAPE data for Ty1 gRNA in the cytoplasm and the in vitro state [24] were recalculated using the SuperFold v1.1 to avoid biases resulting from algorithm differences. In brief, SuperFold v1.1 software uses Partition and Fold functions implemented in RNA structure together with the SHAPE reactivities input as pseudo-energy constraints [32,33] for prediction of the MFE structure model, calculation of base pairing probabilities for all possible canonical base pairs, and identification of well-defined regions (lowSS). Slope and intercept folding parameters were set as the default (1.8 and −0.6 kcal mol^−1^, respectively) for each calculation. A partition function was computed in 1200-nt sliding windows with the increment of 100 nts, the pairing distance was limited to 600 nts, and the four additional calculations were performed on the 5′ and 3′ ends of the nuclear Ty1 gRNA. Moreover, 300 nts were trimmed from each window to exclude the end effect. Base-pair probabilities from multiple windows were combined. Shannon entropies were calculated from individual base-pairing probabilities and combined into a single, 55-nt sliding windows profile. The MFE structure was predicted with the SHAPE data as pseudo-energy constraints, and base pairs with >99% probability were added as hard constraints. Fold was run in 3000-nt sliding windows with 300 nt-increments, and the pairing distance was set to 600 nts. Four additional fold calculations were performed on the 5′ and 3′ ends, as described above. The final MFE structure was combined from multiple windows with the requirement that base pairs must appear in a majority of the windowed folds. Well-defined regions (lowSS) were identified by selecting regions with at least 40 nts with both median SHAPE and median Shannon entropy values below their respective medians. Some lowSS regions were combined or expanded to include the entirety of helixes predicted in MFE. RNA structures were visualized using VARNA [34]. Additionally, potential pseudoknot formation was analyzed with ShapeKnots [35], in which full-length nuclear Ty1 gRNA was folded in 600-nt sliding windows with 100-nt increments. Pseudoknots occurring in >50% of the windows were included in the MFE structure. 

### 2.6. Signal-To-Background Ratio and Correlation Calculation

The signal-to-background (S/B) ratio for NMIA and NAI modification in the yeast nucleus was calculated based on ShapeFinder raw output files. The signal and background peak area values for each nucleotide were averaged, and their medians were estimated. S/B was then calculated as the ratio between the medians of signal and background. Spearman’s correlations and linear regressions were computed with GraphPad Prism 8 software.

### 2.7. Comparison of SHAPE Reactivities and SHAPE-Directed Structural Models of Ty1 gRNA

Sensitivity and PPV values for the entire nuclear Ty1 gRNA MFE model were calculated relative to the cytoplasmic or in vitro Ty1 gRNA MFE using the Scorer function implemented in RNAStructure. To ensure that compared structures have identical sequence, the adenine at position 58 in the frameshift mutant Ty1fs was manually deleted from cytoplasmic and in vitro Ty1 MFE structures. For identified lowSS regions, sensitivity and PPV values were computed relative to the nuclear Ty1 gRNA MFE. If the region of interest holds only a half of the predicted base pairings, nucleotides from the disrupted helix were added manually to the calculations. SHAPE reactivities for Ty1 gRNA in virio were reported previously [36]. For comparative analysis of nuclear and in virio Ty1 gRNAs, deconvolution of in virio SHAPE reactivities was performed by multiplying them by 0.6, the ratio between SHAPE reactivity medians for nuclear and in virio gRNAs. 

### 2.8. Computing Regions of Large Absolute SHAPE Reactivities Changes

The absolute value of the SHAPE reactivity difference between nuclear and cytoplasmic compartments was summed over 75-nt sliding windows. Spans of at least 100 consecutive nucleotides in which absolute reactivity changes were above the global median change were characterized as regions with the largest SHAPE reactivity differences.

## 3. Results

### 3.1. NMIA Successfully Modifies RNA in the Yeast Cell Nucleus

**Figure 1:**
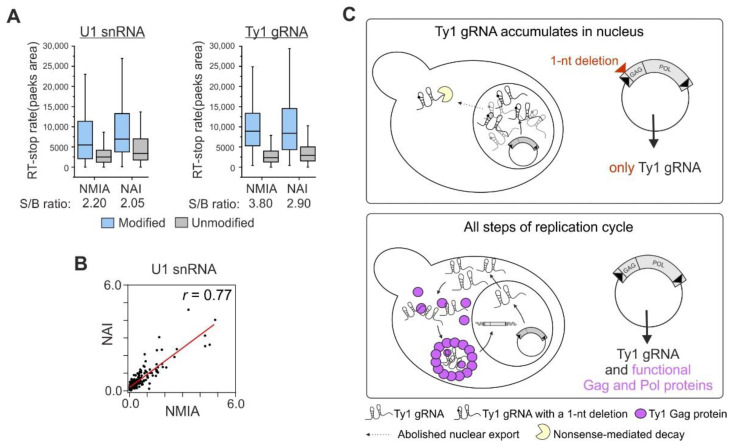
Yeast strains used for Ty1 gRNA structural studies and analysis of the ability of NMIA to modify yeast nuclear RNA. (**A**) Box plot analysis of RT-stop rate measurement with medians for signal and background for U1 snRNA and Ty1 gRNA. Plots present data for approximately 340 nts and 400 nts of U1 snRNA and Ty1 gRNA, respectively. (**B**) Correlation of position-dependent NMIA and NAI reactivities for U1 snRNA (data for 230 nts). (**C**) Comparison of DG3408 (pGTy1fs) and DG3412 (pGTy1 wild type) yeast strains.

SHAPE mapping using an appropriate electrophile to acylate the ribose 2′-hydroxyl groups efficiently interrogates the flexibility of local RNA structures in vitro and in vivo [37]. Several well-validated SHAPE reagents with different cell permeabilities, half-lives, and nucleotide biases are currently used for RNA structure mapping in cells [38,39,40]. In the case of Ty1 gRNA, all prior structural studies were performed using N-methylisatoic anhydride (NMIA) [15,22,24,36,41,42]. For structural comparisons, we also used NMIA to determine the secondary structure of nuclear Ty1 gRNA. Application of the same probing reagent allowed more reliable comparisons of Ty1 gRNA structural transitions between cell compartments or biological states. Recently, we showed that NMIA penetrates yeast cells and effectively modifies cytoplasmic transcripts [24], but its capacity to modify nuclear RNA was not investigated. To that end, we performed structural mapping of the U1 small nuclear RNA (snRNA) in the well-characterized *S. cerevisiae* strain BY4742 using NMIA and 2-methylnicotinic acid imidazolide (NAI)—SHAPE reagent previously shown to be suitable for RNA probing inside the nucleus of mammalian cells [40]. Using capillary electrophoresis to detect modification-induced reverse transcriptase-stops, we obtained high and comparable signal-to-background (S/B) ratios for both SHAPE reagents, showing that NMIA robustly modifies nuclear RNAs in yeast with efficiency similar to NAI (Figure 1A). The correlation between position-dependent NMIA and NAI reactivities in U1 snRNA (r = 0.77) showed that there is a strong similarity in the modification patterns (Figure 1B), but some nucleotide biases were also observed.

### 3.2. The Experimental Strategy Used to Explore Ty1 gRNA Structure in the Nucleus

To determine the structure of Ty1 gRNA in the yeast nucleus, we used a previously-characterized yeast strain—DG3408—that naturally lacks chromosomal Ty1 elements, and expression of Ty1 occurs exclusively from a galactose inducible plasmid pGTy1fs (pBDG1112, pGTy1gag-ATGfs) [13] (Figure 1C). Ty1fs contains a single “A” deletion adjacent to the GAG start codon, which profoundly affects Ty1 gRNA localization in the cell. After galactose induction, pGTy1gag-ATGfs produces full-length Ty1fs transcripts (5651 nts) but, as expected for a frame-shift mutation, does not synthesize complete Gag and Pol proteins. Due to the lack of functional Gag, Ty1 gRNA accumulates in the nucleus, its cytoplasmic level and stability are greatly decreased, and retrosomes and VLPs are not detected [13]. This strain enabled us to obtain a more homogenous pool of correctly initiated nuclear Ty1 transcripts that were subjected to SHAPE analysis using NMIA. We obtained high-quality SHAPE data (S/B = 3.8) for over 93% of the nucleotides in nuclear Ty1 gRNA, further demonstrating that NMIA is suitable for mapping RNA structure in the yeast nucleus (Figure 1A).

To investigate how the structure of LTR-retrotransposon gRNA changes during replication in host cells, we performed a detailed comparison of SHAPE data for the nuclear and cytoplasmic Ty1 gRNA states [24]. We can exclude the impact of strain-specific cellular factors on gRNA folding because yeast strains applied here and for cytoplasmic Ty1 gRNA studies are based on the same parental yeast strain (DG1768) [43] and differ only by the point mutation in the pGTy1fs plasmid. In contrast, wild type pGTy1 transcripts expressed in DG3412 are efficiently exported from the nucleus, the majority of gRNA is in the cytoplasmic compartment, and small fractions are detected in the nucleus or in VLPs (Figure 1C). We also compared the structure of nuclear Ty1fs gRNA to those determined in VLPs or under in vitro conditions [24,36].

### 3.3. Analysis of SHAPE Structural Data for Nuclear Ty1 Grna

**Figure 2:**
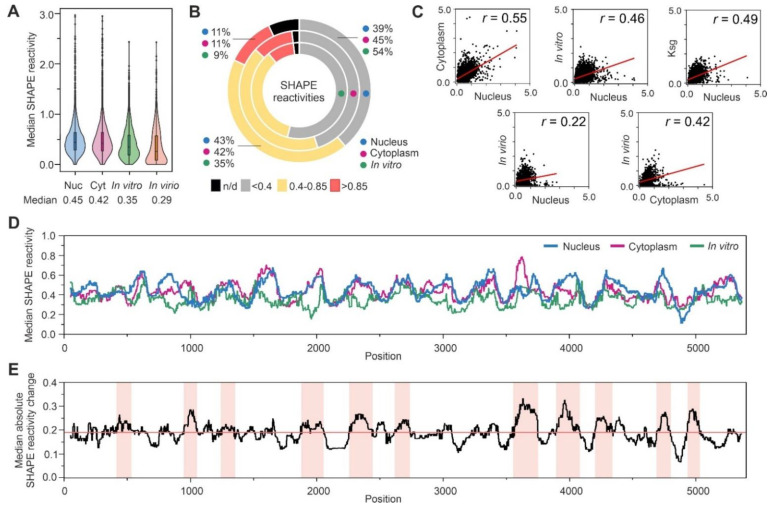
Analysis of SHAPE reactivities for nuclear Ty1 gRNA compared to cytoplasmic and in vitro full-length Ty1 gRNAs, the inhibition of translation initiation state (for 2500 nts), and the **in virio** state (for 1482 nts). (**A**) Violin plot analysis of SHAPE reactivity distributions with medians. (**B**) Pie chart analysis of SHAPE reactivity percentage distribution. (**C**) Correlation of position-dependent NMIA reactivities. (**D**) The median SHAPE reactivity profiles smoothed within a 75-nt window. (**E**) The median profile of absolute SHAPE reactivity change between nuclear and cytoplasmic datasets, smoothed within a 75-nt window. Light-red shadings indicate regions with the greatest absolute differences above the global median change (marked in red) (see Section 2).

We compared the overall SHAPE reactivity distribution in different Ty1 gRNA states (Figure 2A). The distribution of reactivity in the nuclear RNA was more similar to the cytoplasmic state than the in vitro or in virio (within a VLP) states [24,36]. Accordingly, the median SHAPE reactivity of the nuclear Ty1 gRNA was comparable to cytoplasmic gRNA (0.45 and 0.42, respectively), and they were higher than in vitro gRNA (0.35) or in VLPs (0.29) (Figure 2A). Analysis of the percentage reactivity distributions showed that ~11% of nucleotides displayed a high SHAPE reactivity (>0.85), 43% were intermediate (0.4–0.85), and the remaining 39% were unreactive (< 0.4) in the nuclear state (Figure 2B). This SHAPE reactivity content is comparable to the cytoplasmic Ty1 gRNA but different from gRNA transcribed and folded in vitro. Nucleotides with a low SHAPE reactivity are likely base-paired, and highly reactive nucleotides are placed in single-stranded RNA regions, whereas those with intermediate SHAPE reactivity can be involved in alternate pairing. Together, these results suggest that independent of the compartment, Ty1 gRNA is less structured than after packaging into VLPs or under in vitro conditions.

To follow Ty1 gRNA structural changes between cell compartments, we calculated the position-dependent correlation between their SHAPE reactivities (Figure 2C). We found that this correlation was moderate (r = 0.55), suggesting that significant Ty1 gRNA structure remodeling occurred after nuclear export. The reactivity correlation between nuclear and VLPs states was even lower (r = 0.22), implying increased structural differences. Interestingly, the correlation between reactivities in the cytoplasm and VLPs was 0.42 and suggested a slightly higher similarity in Ty1 gRNA structure than that observed between nuclear and in virio states. In an attempt to identify factors contributing to the differences observed between nuclear and cytoplasmic Ty1 gRNA structures, we compared SHAPE data for nuclear Ty1 gRNA with those obtained in cells partially inhibited for translation by kasugamycin treatment [24]. However, we did not detect an increase in the position-dependent correlation between SHAPE reactivities that would indicate a higher structural similarity between nuclear and cytoplasmic Ty1 gRNA in the presence of kasugamycin, which blocks translation initiation [44] (Figure 2C). Moreover, we did not detect any increase in correlation when nuclear data were compared to those from the in vitro state.

Analysis of SHAPE reactivity profiles confirmed the substantial differences detected between many regions of the nuclear, cytoplasmic, or in vitro Ty1 gRNA states (Figure 2D). To estimate the range of SHAPE reactivity alterations between compartments, we calculated the absolute reactivity changes between them, and we searched for Ty1 gRNA regions of at least 100 consecutive nucleotides with the absolute change exceeding the median change (Figure 2E). Based on this approach, we detected 11 regions evenly distributed across the *GAG* and *POL* ORFs. Such extensive reactivity alterations support compartment-specific folding of Ty1 gRNA rather than differences in RNA/protein interactions or RNA modification patterns that usually induce more local changes [45].

### 3.4. The MFE Structure Model of the Nuclear Ty1 RNA Genome

**Figure 3:**
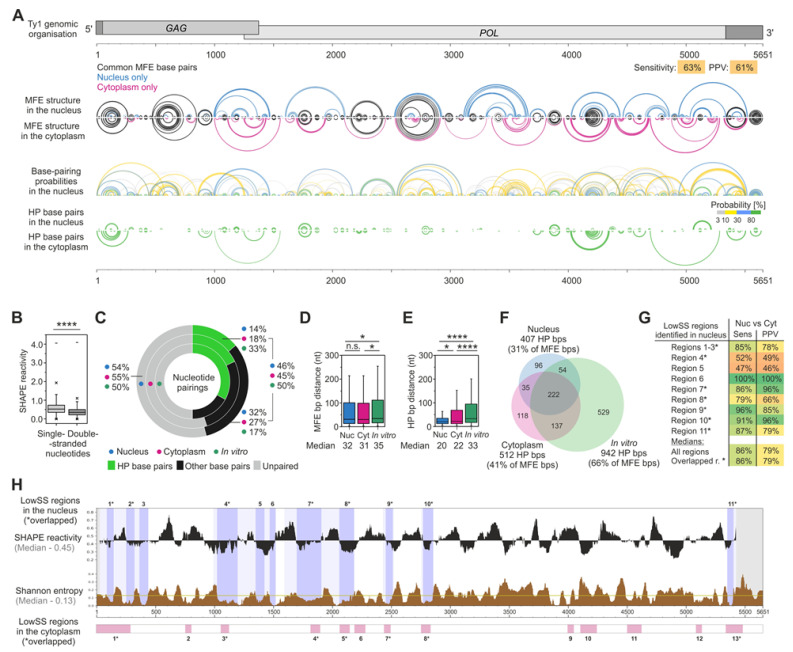
SHAPE-directed structure model of the nuclear Ty1 gRNA and its comparison with MFE structures predicted for cytoplasmic or in vitro Ty1 gRNAs. (**A**) Genomic organization of Ty1 gRNA. (**B**) Box plot analysis of SHAPE reactivities mapped to single- and double-stranded regions of the nuclear Ty1 gRNA MFE structure. (**C**) Pie chart analysis of percentage distributions of nucleotide pairings in the MFE structures, including high probability base pairs (HP bps, pairing probability > 80%). (**D**) Box plot analysis of MFE bps and (**E**) HP bps distance with medians. (**F**) Venn diagram showing overlap of HP bps. (**G**) Sensitivity and PPV parameters for lowSS regions identified in nuclear Ty1 gRNA. (**H**) LowSS regions analysis. Light shadings indicate extending to encompass entire intersecting helices from MFE structures. The bottom bars present locations of lowSS regions in the cytoplasmic Ty1 gRNA. Overlapped regions are marked by *. Significance was computed by unpaired two-tailed Mann–Whitney test; **** *p* < 0.0001. * *p* < 0.05; n.s., not significant.

To better understand Ty1 gRNA folding inside the nucleus, we used the SuperFold pipeline to perform SHAPE-directed windowed modeling of the minimum free energy (MFE) RNA structure [32] (Figure 3A). Examination of SHAPE reactivities in single- and double-stranded regions confirmed the agreement between the predicted structural model and the SHAPE data (Figure 3B). By comparing the nuclear MFE structure to that of cytoplasmic Ty1 gRNA in terms of sensitivity (sens) and positive predictive value (PPV), we found that about 60% of MFE bps predicted for the nuclear state also appeared in the cytoplasmic state (Figure 3A). Most of these shared bps represented short-range interactions, whereas the long-distance pairings seemed unique for each compartment. About 46% of nucleotides are involved in base-pairing in both MFE structure models (Figure 3C), and we did not find significant differences in the median pairing distances or numbers of long-distance pairings (Figure 3D). 

To consider the complete ensemble of possible Ty1 gRNA structures in the nucleus, we calculated the probability of each base-pairing in the MFE model (Figure 3A). Highly probable base pairs (HP bps; >80% of probability) are the most likely to be present in all coexisting structural conformers of an RNA molecule, whereas those with a low probability are much rarer. We identified 407 HP bps, which constitute 31% of all MFE bps (Figure 3F). For comparison, about 100 more HP bps have been identified in the cytoplasmic Ty1 gRNA, and they constituted 41% of all MFE bps [24]. Interestingly, we did not observe a tendency to preserve HP bps between compartments (63% of shared HP bps) compared to other bps. Also, nuclear Ty1 gRNA was depleted for long-range HP bps (>100 nt) (Figure 3A). The median HP pairing distances in both compartments remained similar (20 and 22, respectively), and they were much lower than the median distances calculated for MFE bps (Figure 3E). In agreement with the above findings, the median Shannon entropy of nuclear Ty1 gRNA was higher than that calculated for the cytoplasmic state (0.13 and 0.10, respectively) (Figure 3H) [24]. Taken together, our data suggests that nuclear Ty1 gRNA is less structured and in a more heterogenous state.

To better understand the structural heterogeneity of nuclear Ty1 gRNA, we determined the most compact and thermodynamically stable RNA regions. Based on low SHAPE reactivity and Shannon entropy values (lowSS framework) [46], we identified 11 well-structured regions that cover about 19% of Ty1 gRNA in the nucleus (Figure 3H). We found that most overlap, at least partially, with lowSS regions detected in cytoplasmic Ty1 gRNA. For most lowSS regions, the PPV and sensitivity metrics are higher than for the entire MFE structure, indicating that stable, highly structured motifs tend to be preserved after the export of Ty1 gRNA from the nucleus to the cytoplasm (Figure 3G). 

We also compared the nuclear Ty1 gRNA MFE structure to that determined under defined in vitro conditions [24]. Calculated PPV and sensitivity parameters were 51% and 47%, respectively, indicating that only about half of MFE bps are shared between the nuclear and the in vitro Ty1 gRNA structure models (Figure S1). Even though the percentage of nucleotides engaged in base-pairings in both structures was similar, the nuclear MFE model has 535 fewer HP bps than the in vitro model (Figure 3C,F). Consistently, the median Shannon entropy for gRNA in the nucleus was much higher than in vitro (∆_Shannon_ = 0.09). Based on the lowSS approach, we were able to identify 4 lowSS regions that were partially shared in these states of Ty1 gRNA (Figure S1). Therefore, our results further support the idea that there are substantial differences in Ty1 gRNA folding in vivo and in vitro.

### 3.5. RNA–RNA Interactions Mediated by Gag In Vivo

**Figure 4:**
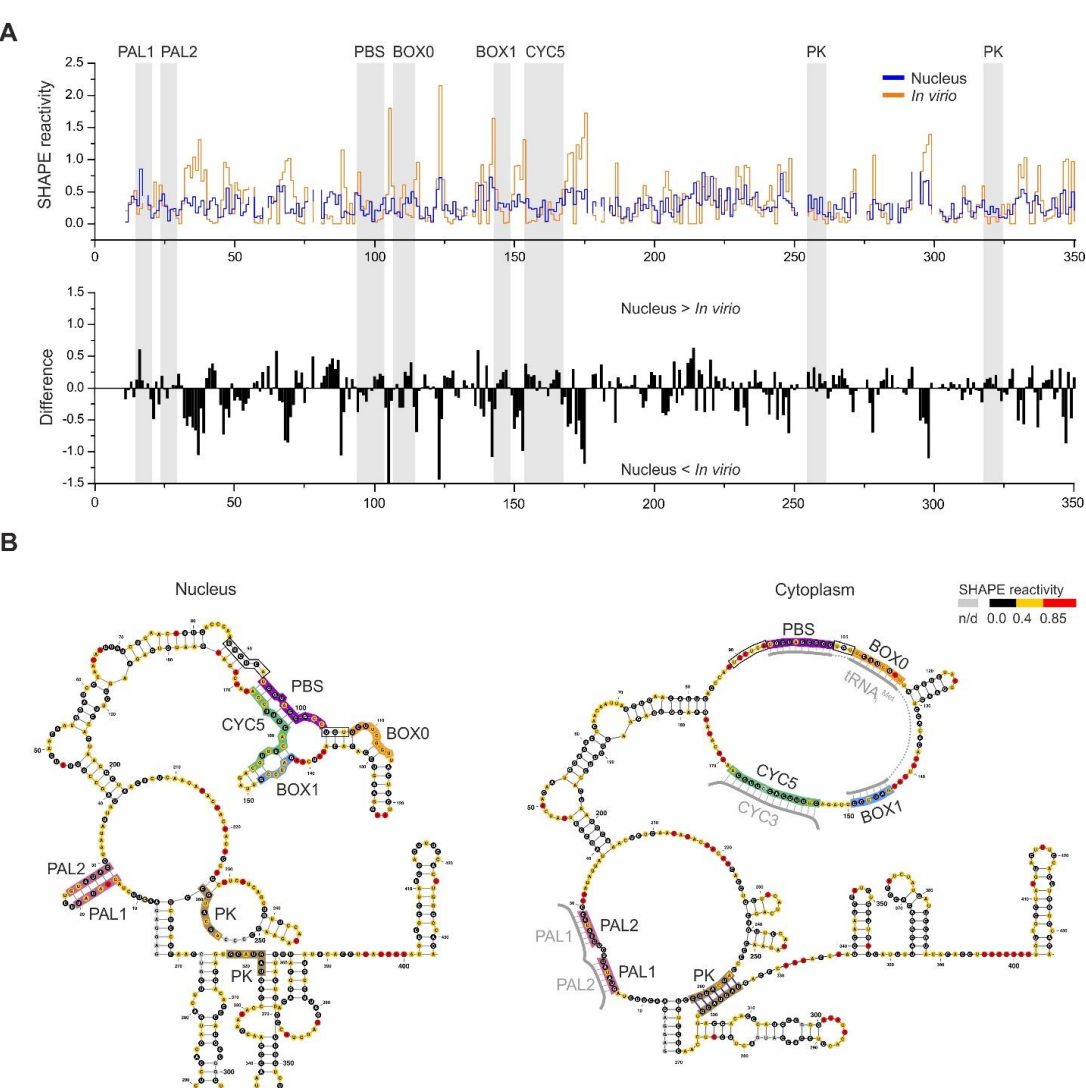
Comparative analysis of Ty1 gRNA regions containing cis-acting sequences. (**A**) The step plot (top) and the difference plot (down) calculated by subtracting the in virio NMIA reactivities from those of the nuclear Ty1 gRNA. (**B**) The MFE models of nuclear and cytoplasmic Ty1 gRNA 5′ end. Cis-acting sequences are marked with colored boxes. Neighboring regions affected by tRNA_i_^Met^ annealing are boxed.

The 5′ terminus of Ty1 gRNA contains well-characterized cis-acting sequences required for the efficient gene expression and propagation of the element. These include the following motifs: PBS, BOX0, and BOX1 necessary for tRNA_i_^Met^ binding; CYC5 involved in genome cyclization; and palindromes PAL1 and PAL2 required for Ty1 gRNA dimerization [9,11] (Figure 4). In vitro analyses suggest that these crucial RNA–RNA interactions are promoted by Ty1 Gag [14,15,17]. However, Ty1 Gag’s role during retrotransposition has not been demonstrated. To address this issue based on changes in Ty1 gRNA structure, we compared SHAPE data obtained in vivo in the absence of Gag to that from VLPs and the cytoplasm, where Ty1 gRNA is dimerized, cyclized, and tRNA is annealed, and to the in vitro state, where Gag-mediated RNA–RNA interactions do not occur [24,36]. 

In the absence of tRNA_i_^Met^ in vitro, the PBS is unreactive due to an intramolecular interaction with CYC5, and segments of BOX0 and BOX1 remain unpaired and accessible to SHAPE modification [36]. Although the PBS remains unreactive independent of primer binding, SHAPE can detect disruption of helical regions adjacent to PBS resulting from tRNA_i_^Met^ hybridization [24,36]. Therefore, increased reactivity in these regions can serve as an indicator for tRNA_i_^Met^ binding. In the nucleus, we did not detect increases in reactivity in the region proceeding PBS or in the 3-nt sequence linking PBS and BOX0 (Figure 4A,B—black frames). Also, we observed increased reactivity in BOX0 and BOX1. These data do not support Ty1 gRNA/tRNA_i_^Met^ complex formation in the nucleus and strongly agree with other studies suggesting that complex formation occurs in the cytoplasm [47,48,49] (Figure 4B). Our results also argue against Ty1 gRNA cyclization occurring in the nucleus, as engagement of CYC5 in interaction with CYC3 would lead to increased reactivity of the PBS when tRNAiMet is not annealed. 

Ty1 gRNA dimerization is mediated by cis-acting palindromic sequences PAL1 and PAL2 [15,36]. In the dimeric state, these sequences remain unreactive due to Gag-mediated PAL1/PAL2 intermolecular interactions. In monomeric gRNA, PALs are also unreactive due to their intramolecular interaction, leading to stem-loop formation. These two distinct interactions can be distinguished based on reactivity changes in nucleotides linking PAL1 and PAL2 (21UCU23). For nuclear Ty1 gRNA, we observed 21UCU23 reactivity similar to that in the dimeric state; however, we also detected increased SHAPE-reactivity in the PALs (Figure 4A). Thus, our data suggest that in the absence of Gag, PAL1/PAL2 intermolecular duplexes are not formed, and the stem-loop with PALs is labile in the Ty1 gRNA monomer (Figure 4B). 

In VLPs and the cytoplasmic state, the 5′end of Ty1 gRNA forms a functionally important H-type pseudoknot (PK), where nucleotides at positions 1–7 and 256–262 form base pairs with nucleotides 264–270 and 319–325, respectively [36,41]. The PK has not been detected under in vitro conditions [24,36]. With nuclear Ty1 gRNA, we observed slightly increased reactivity in the sequences involved in PK formation when compared to VLPs (Figure 4A). Also, we did not detect this important PK interaction in nuclear Ty1 gRNA using ShapeKnot software and nuclear SHAPE data as constraints (Figure 4B).

## 4. Discussion

Due to the rapid development of RNA probing techniques, the structures of several viral RNAs in distinct experimental conditions have been resolved, e.g., [50,51,52,53,54]. These studies reveal meaningful differences between RNA folding in vitro and in cells and highlight the importance of studying RNA structures in their native cellular context. However, they provide an averaged “steady-state” picture of viral RNA in the cell that may fail to resolve functionally important structural transitions during replication. Consequently, little is known about whether and how the structure of viral RNAs change in specific cellular compartments.

Here, we took advantage of a well-developed Ty1 experimental system to resolve the first structure of a retroviral-like RNA in the nucleus. To avoid problems concerning inefficient purification of intact yeast nuclei and the low level of wild type nuclear Ty1 RNA, we utilized a genetically modified element that does not produce Ty1 Gag and accumulates GAL1-promoted Ty1fs RNA in the nucleus [13]. In the absence of Ty1 Gag, Ty1fs RNA that enters the cytoplasm is subjected to robust RNA-turnover pathways including nonsense-mediated decay and degradation by P-body components. We assumed that only a very small, if any, fraction of cytoplasmic Ty1fs RNA could be mapped together with nuclear Ty1fs RNA and strongly believe that the modification signals from this small cytoplasmic fraction do not significantly interfere with our data. Through a direct comparison of SHAPE-derived nuclear Ty1 gRNA structure with those available for Ty1 gRNA in the cytoplasm, within purified VLPs, or synthesized and folded in vitro [24,36], we characterized gRNA structural alterations that occur during Ty1 retrotransposition. Finally, we confirm and extend the role of Gag during RNA–RNA interactions crucial to Ty1 replication in budding yeast.

In support of our previous findings that the cellular environment greatly impacts Ty1 RNA architecture [24], we observe significant structural differences between nuclear and in vitro Ty1 gRNAs. We also discovered large regions with extensive structural alterations between nuclear and cytoplasmic Ty1 gRNAs, indicating compartment-specific folding. mRNA structural changes between compartments have been identified by transcriptome-wide study in the mammalian cells, but in contrast to our observations, most are relatively small and mainly local, suggesting that RNA structures formed during transcription remain largely unchanged during the mRNA life span [45]. However, transcriptome-wide analyses may not reveal the extent of all structural variations for individual RNAs. Thus, Ty1 gRNA is an example of a transcript sensitive to the local nuclear and cytoplasmic environments, and its structure undergoes significant remodeling during the transition between cell compartments. Based on base-pairing probabilities and Shannon entropy calculations, we show that Ty1 gRNA inside the nucleus adopts a more heterogeneous structure than in the cytoplasm. Interestingly, we show that short-range interactions represent the majority of nuclear HP bps, whereas long-range HP bps likely form once Ty1 gRNA exits the nucleus. Our observations are consistent with previous findings regarding cotranscriptional RNA folding, suggesting that short-range base pairs are formed immediately after transcription, whereas long-range interactions are more likely to form transitory, less-probable structures, which refold during RNA maturation and transition between cell compartments [55,56,57]. 

Although Ty1 gRNA displays a higher degree of structural heterogeneity in the nucleus, we identified 11 thermodynamically stable regions (lowSS regions) that significantly overlap with those in the cytoplasm [24]. Prior studies show that such structurally stable motifs are often overrepresented with functionally important cis-acting sequences [46,53,58,59,60]. Most of the cis-acting Ty1 sequences are also placed in lowSS regions of the gRNA, but the functions of other stable regions remain unknown. Our observations suggest that gRNA regions likely necessary for Ty1 retrotransposition adopt well-defined structures during or just after transcription, and they remain unchanged across compartments. However, we also noticed that several nuclear lowSS regions are no longer detected in the cytoplasm where new lowSS regions are detected. Therefore, our results also support a recent study suggesting that some functionally important RNA structural motifs fold differently in the nucleus and cytoplasm [61]. 

The exact mechanisms by which RNA structures are modulated in vivo are not well understood. While some studies suggest that translation is the primary force responsible for the RNA structural remodeling, others highlight the importance of RNA binding proteins or RNA modifications [45,62]. We previously found that the ribosomes are an important factor shaping the Ty1 RNA structure in the cytoplasm [24]. However, here we show that active translation does not explain Ty1 gRNA structural alterations in different cellular compartments. On the other hand, we detected greater structural similarities between Ty1 gRNA in the cytoplasm and VLPs than between nuclear Ty1 gRNA and VLPs. As Ty1 Gag protein displays RNA chaperone activity and is present in the cytoplasm and VLPs, but not in the nucleus, we conclude that Gag may account for the higher gRNA structural similarity between the cytoplasm and VLPs. Analogous to HIV-1 Gag, interactions between Ty1 Gag and retrotransposon gRNA may vary at different stages of retrotransposition and induce diverse structural effects [63]. Although we cannot fully explain a Gag’s role in Ty1 gRNA structure remodeling, our comparative analyses of SHAPE data confirmed and extended the critical role for Gag in Ty1 gRNA dimerization, cyclization, and primer tRNA annealing in vivo. Ty1 Gag does not contain a nuclear localization signal and is detected near but not within the nuclei of cells expressing Ty1 [13]. Thus, we show RNA–RNA interactions essential for retrotransposition cannot occur prior to Ty1 gRNA nuclear export to the cytoplasm and interaction with Gag. 

Studying RNA structures in cellular compartments is essential to gain a comprehensive view of the complex interplay between RNA structure and function. Our work is the first to present the nuclear structure of a viral-like gRNA, although it may be premature to conclude whether compartment-specific gRNA folding is a general feature of LTR-retrotransposons or other reverse-transcribing single-strand RNA viruses. However, additional RNA structural studies across cellular compartments are needed to define the common or specific mechanisms of viral and retrotransposon gRNA structure modulation.

## Data Availability

Most of the data are provided in this work or in the Supplementary Data 1. Other data that support the findings of this study are available from the corresponding author upon reasonable request.

## Supplementary Materials

The following supporting information can be downloaded at: https://www.mdpi.com/article/10.3390/v14092007/s1. Table S1. Reverse transcription primers. Figure S1. Comparison of SHAPE-directed MFE model predicted for nuclear and Ty1 gRNA in vitro.
