# Supplementary material for: Cell Compartment-Specific Folding of Ty1 Long Terminal Repeat Retrotransposon RNA Genome
Source: Viruses. 2022 Sep 10;14(9):2007. doi: 10.3390/v14092007 (PMC9503155; doi:10.3390/v14092007)
Supplement: Supplementary file 1 [file viruses-14-02007-s001.zip › Supplementary_Data_Zawadzka_et_al.pdf]

nt		nuclear Ty1	SD	nt		U1 NMIA	SD	U1 NAI	SD
1	G	-	0.0000	1-128		-	0.0000	-	0.0000
2	A	-	0.0000	129	C	0.004	0.0028	0.235	0.2694
3	G	-	0.0000	130	C	0.217	0.1428	0.145	0.1237
4	G	-	0.0000	131	U	1.394	0.2652	3.045	0.1603
5	A	-	0.0000	132	U	0.203	0.0099	0.554	0.0184
6	G	-	0.0000	133	G	0.34	0.0594	0.687	0.2277
7	A	0.378	0.0750	134	G	0.409	0.0021	0.825	0.3147
8	A	0.145	0.0000	135	U	0.753	0.1640	-	0.0000
9	C	0.390	0.3528	136	C	0.129	0.0064	0.287	0.1315
10	U	0.383	0.0078	137	A	0.085	0.0311	0.216	0.1803
11	U	0.051	0.0456	138	C	0.009	0.00636	0.051	0.0113
12	C	0.500	0.2141	139	A	0.093	0.0120	0.306	0.0170
13	U	0.724	0.2009	140	C	0.078	0.0423	0.017	0.0214
14	A	0.635	0.0746	141	A	0.032	0.0417	0.17	0.0028
15	G	0.469	0.1392	142	C	0.109	0.0679	0.181	0.1061
16	U	1.423	0.4215	143	A	0.282	0.1146	0.498	0.0304
17	A	0.736	0.1042	144	C	-	0.0000	0.156	0.1287
18	U	0.157	0.0881	145	A	-	0.0000	0.03	0.0191
19	A	0.485	0.0250	146	U	-	0.0000	0.272	0.0820
20	U	0.154	0.0930	147	A	-	0.0000	0.049	0.0120
21	U	0.281	0.0685	148	C	0.114	0.0750	0.208	0.1131
22	C	0.342	0.0637	149	G	0.168	0.0354	0.296	0.3097
23	U	0.608	0.2661	150	G	0.252	0.0219	0.334	0.1358
24	G	0.784	0.1652	151	C	0.327	0.0120	0.512	0.0792
25	U	0.454	0.1019	152	G	0.454	0.1704	0.425	0.0134
26	A	0.074	0.0341	153	C	0.446	0.0955	1.051	0.5240
27	U	0.380	0.1035	154	G	0.239	0.1308	0.145	0.0877
28	A	0.395	0.0574	155	G	-	0.0000	-	0.0000
29	C	0.223	0.0831	156	A	-	0.0000	0.49	0.0651
30	C	0.371	0.0758	157	A	-	0.0000	0.134	0.1633
31	U	0.594	0.0917	158	G	-	0.0000	0.37	0.1025
32	A	0.671	0.1048	159	G	0.005	0.0042	0.075	0.0983
33	A	0.543	0.1149	160	C	2.102	0.2772	1.917	0.08254
34	U	0.471	0.1165	161	G	2.216	0.3769	2.361	0.09041
35	A	0.708	0.1651	162	U	0.321	0.1259	0.548	0.0665
36	U	0.285	0.1140	163	G	0.32	0.0615	0.603	0.1358
37	U	0.444	0.2124	164	U	-	0.0000	0.032	0.0000
38	A	0.600	0.1336	165	U	-	0.0000	0.167	0.0000
39	U	0.231	0.0837	166	U	-	0.0000	0.612	0.0000
40	A	0.385	0.1830	167	G	-	0.0000	-	0.0000
41	G	0.597	0.2254	168	C	0.013	0.0099	0.172	0.2305
42	C	0.667	0.2060	169	U	0.233	0.18573	0.188	0.1739
43	C	0.498	0.1863	170	G	0.969	0.1916	1.076	0.0226
44	U	0.163	0.0499	171	A	0.358	0.1619	0.555	0.1952
45	U	0.185	0.0921	172	C	0.255	0.1160	0.493	0.0007
46	U	0.397	0.0560	173	G	0.052	0.00579	0.275	0.2956
47	A	0.933	0.1086	174	U	0.157	0.13923	0.412	0.0969
48	U	0.260	0.0983	175	U	0.125	0.1654	0.371	0.2489
49	C	0.386	0.0251	176	U	0.018	0.0274	0.372	0.0170

50	A	0.757	0.1116	177	C	0.139	0.0000	0.276	0.0276
51	A	0.664	0.1019	178	C	-	0.0000	0.206	0.0332
52	C	0.220	0.1304	179	A	-	0.0000	0.222	0.0544
53	A	0.309	0.0670	180	U	-	0.0000	0.442	0.1039
54	A	0.505	0.1834	181	U	-	0.0000	0.420	0.1295
55	U	0.693	0.0872	182	C	-	0.0000	0.345	0.3635
56	G	0.091	0.0615	183	C	0.037	0.0502	0.135	0.1287
57	G	0.168	0.1005	184	C	0.953	0.2256	1.284	0.0361
58	A	0.202	0.0696	185	U	0.22	0.1039	0.426	0.2666
59	U	0.140	0.0840	186	U	0.229	0.1039	0.42	0.1442
60	C	0.422	0.0594	187	G	0.316	0.0431	0.262	0.0509
61	C	0.293	0.0384	188	U	0.367	0.2065	0.56	0.2454
62	C	0.715	0.1973	189	U	0.164	0.0841	0.358	0.0530
63	A	0.537	0.1093	190	U	0.169	0.0150	0.392	0.0460
64	A	0.584	0.0730	191	C	0.094	0.0799	0.389	0.0509
65	C	0.980	0.2720	192	A	0.099	0.0424	0.259	0.2447
66	A	0.832	0.0732	193	A	1.251	0.3352	1.269	0.2065
67	A	0.988	0.0702	194	U	0.079	0.08121	0.231	0.1591
68	U	0.275	0.1552	195	C	0.024	0.03109	0.308	0.1245
69	U	0.285	0.1382	196	A	0.144	0.0884	0.303	0.0651
70	A	0.388	0.1278	197	U	1.285	0.0707	1.133	0.2447
71	U	0.270	0.0738	198	U	3.552	0.7269	2.530	0.6608
72	C	0.535	0.1047	199	G	0.394	0.0177	0.464	0.2595
73	U	0.182	0.1002	200	G	0.262	0.0460	0.236	0.2220
74	C	0.413	0.0935	201	U	0.163	0.0926	0.28	0.0544
75	A	0.574	0.0478	202	U	0.053	0.0218	0.151	0.1520
76	A	0.589	0.0780	203	A	0.125	0.0791	0.259	0.1605
77	C	0.474	0.1565	204	A	0.072	0.0856	0.224	0.1230
78	A	0.877	0.0934	205	U	0.122	0.0636	0.583	0.0035
79	U	0.307	0.0431	206	C	0.046	0.0141	0.098	0.0269
80	U	0.127	0.0418	207	C	0.109	0.0806	0.129	0.1252
81	C	0.145	0.0532	208	C	0.191	0.0170	0.332	0.1315
82	A	0.552	0.2137	209	U	0.173	0.0151	0.453	0.0339
83	C	0.694	0.3007	210	U	0.239	0.0210	0.389	0.2751
84	C	0.828	0.3940	211	G	-	0.0000	0.2	0.0403
85	C	0.776	0.2316	212	A	0.014	0.0007	0.149	0.1068
86	A	0.502	0.0908	213	U	0.321	0.0926	0.348	0.2397
87	A	0.743	0.0463	214	U	0.569	0.1563	0.909	0.2220
88	U	0.163	0.1249	215	C	0.124	0.0877	0.157	0.1322
89	U	0.326	0.0649	216	C	0.095	0.0923	0.201	0.0778
90	C	0.283	0.0977	217	U	0.119	0.0642	0.391	0.0955
91	U	0.248	0.0625	218	U	0.112	0.0212	0.565	0.1789
92	C	0.237	0.0969	219	U	0.532	0.0226	1.195	0.2786
93	A	0.802	0.4830	220	G	0.74	0.1633	1.183	0.5233
94	U	0.735	0.2588	221	G	1.263	0.7241	0.888	0.1407
95	G	0.351	0.0749	222	G	-	0.0000	1.492	0.3168
96	G	0.210	0.1257	223	G	-	0.0000	-	0.0000
97	U	0.253	0.0624	224	A	-	0.0000	-	0.0000
98	A	0.574	0.1527	225	U	-	0.0000	-	0.0000
99	G	0.059	0.0519	226	U	0.236	0.23059	0.58	0.2298

100	C	0.360	0.1448	227	U	0.076	0.07799	0.53	0.0757
101	G	0.332	0.1370	228	U	0.016	0.0140	0.01	0.0049
102	C	0.505	0.1208	229	U	1.293	0.3055	1.172	0.2758
103	C	0.682	0.1491	230	G	0.234	0.1336	0.317	0.1379
104	U	0.271	0.1003	231	G	0.268	0.0962	0.258	0.0198
105	G	0.340	0.0482	232	G	1.724	0.3224	1.406	0.0000
106	U	0.660	0.1198	233	U	0.332	0.2560	0.621	0.1181
107	G	0.258	0.0720	234	U	0.172	0.0000	0.311	0.1633
108	C	0.311	0.1131	235	A	0.283	0.0523	0.923	0.1018
109	U	0.163	0.0631	236	A	0.09	0.0403	0.706	0.0403
110	U	0.517	0.0532	237	A	0.244	0.0488	0.571	0.0049
111	C	0.681	0.2032	238	C	0.308	0.0311	0.269	0.1082
112	G	0.535	0.1317	239	U	1.263	0.1966	1.172	0.1365
113	G	0.841	0.3988	240	G	1.071	0.0163	0.917	0.1633
114	U	0.670	0.2409	241	A	-	0.0000	-	0.0000
115	U	0.463	0.1292	242	U	1.334	0.2850	1.395	0.4815
116	A	0.136	0.0443	243	U	0.363	0.1541	0.579	0.3302
117	C	0.154	0.0401	244	U	0.146	0.0453	0.324	0.2758
118	U	0.426	0.0538	245	U	0.056	0.0410	0.213	0.0863
119	U	0.223	0.0429	246	U	0.37	0.0580	0.615	0.0643
120	C	0.054	0.0492	247	G	0.23	0.0700	0.253	0.0672
121	U	0.432	0.1459	248	G	0.076	0.0134	0.101	0.0134
122	A	0.899	0.1237	249	G	0.074	0.0184	0.069	0.0134
123	A	1.201	0.0726	250	G	1.505	0.0806	2.337	0.3490
124	G	0.349	0.0523	251	C	1.281	0.2001	2.312	0.4272
125	G	0.495	0.1551	252	C	0.484	0.1358	1.139	0.0643
126	A	0.336	0.1082	253	C	0.934	0.1336	0.44	0.0544
127	A	0.264	0.0699	254	U	3.757	0.5798	2.615	0.3461
128	G	0.580	0.3680	255	U	0.851	0.3578	0.824	0.2779
129	U	0.192	0.0439	256	U	0.559	0.4476	0.769	0.3380
130	C	0.107	0.0730	257	G	0.244	0.0000	0.143	0.1110
131	C	0.202	0.0832	258	U	0.163	0.2298	0.392	0.2970
132	A	0.426	0.1093	259	U	0.841	0.3755	0.895	0.2595
133	C	0.371	0.1948	260	U	0.072	0.0373	0.385	0.1082
134	A	0.439	0.1276	261	C	0.188	0.1273	0.231	0.1082
135	C	0.312	0.1769	262	U	0.137	0.1435	0.416	0.0948
136	A	0.613	0.0473	263	U	-	0.0000	0.309	0.0926
137	A	1.000	0.1600	264	C	0.027	0.00508	0.075	0.07136
138	A	1.007	0.1055	265	U	0.161	0.02963	0.665	0.1662
139	U	0.351	0.1092	266	G	0.236	0.0813	0.405	0.0495
140	C	0.597	0.1045	267	C	0.132	0.1096	0.167	0.0948
141	A	1.213	0.1408	268	C	0.171	0.0785	0.23	0.0113
142	A	0.939	0.1294	269	U	0.656	0.0346	1.491	0.1640
143	G	0.362	0.1430	270	G	0.673	0.1280	0.633	0.1301
144	A	0.543	0.0874	271	G	0.227	0.1096	0.312	0.1457
145	U	0.533	0.2705	272	A	0.096	0.0778	0.121	0.1230
146	C	0.436	0.2199	273	G	0.659	0.0771	0.581	0.1287
147	C	0.517	0.1905	274	A	0.855	0.1619	0.793	0.0339
148	G	0.361	0.1944	275	A	0.362	0.2114	0.579	0.1803
149	U	0.593	0.1915	276	G	0.26	0.0594	0.183	0.05674

150	U	0.446	0.3053	277	U	0.459	0.0962	0.794	0.1541
151	A	0.765	0.2277	278	U	0.362	0.1626	1.044	0.0926
152	G	0.571	0.2134	279	U	0.695	0.0891	2.177	0.5664
153	A	0.546	0.2116	280	G	-	0.0000	0.107	0.0071
154	C	0.655	0.2311	281	A	-	0.0000	0.173	0.0629
155	G	0.411	0.1457	282	C	0.112	0.0827	0.15	0.1082
156	U	0.387	0.1241	283	A	0.123	0.0559	0.64	0.0000
157	U	0.405	0.1484	284	C	0.062	0.0000	0.082	0.0000
158	U	0.174	0.0950	285	C	0.239	0.0997	0.269	0.0000
159	C	0.425	0.1608	286	A	0.51	0.0615	0.581	0.0000
160	A	0.618	0.1016	287	A	0.421	0.0000	0.619	0.0000
161	G	0.068	0.0664	288	A	3.737	0.0000	3.133	0.5706
162	C	0.370	0.3815	289	U	0.683	0.1605	1.423	0.7863
163	U	0.367	0.0972	290	U	0.431	0.1725	0.95	0.6314
164	U	0.317	0.1825	291	C	0.528	0.1428	1.092	0.3649
165	C	0.558	0.0692	292	A	0.694	0.0403	1.037	0.5346
166	C	0.665	0.2392	293	A	0.435	0.1648	0.921	0.3776
167	A	0.487	0.2158	294	A	0.417	0.2602	0.686	0.3627
168	A	0.894	0.3048	295	U	0.069	0.0665	0.117	0.1428
169	A	0.815	0.2810	296	U	-	0.0000	0.084	0.0000
170	A	0.929	0.1882	297	G	0.342	0.0085	0.156	0.0332
171	C	0.503	0.2207	298	G	1.329	0.0778	1.109	0.1754
172	A	0.851	0.2328	299	U	0.799	0.0629	1.124	0.0615
173	G	0.841	0.2156	300	G	0.638	0.0559	0.585	0.1697
174	A	0.648	0.1500	301	U	-	0.0000	0.133	0.1577
175	A	0.899	0.1835	302	U	-	0.0000	0.173	0.0742
176	G	0.287	0.1658	303	A	0.39	0.0665	0.578	0.1061
177	A	0.456	0.1162	304	G	0.154	0.0544	0.188	0.0523
178	A	0.572	0.1967	305	G	0.085	0.0134	0.082	0.0092
179	U	0.461	0.1176	306	G	0.056	0.0000	0.075	0.0311
180	G	0.109	0.0321	307	G	0.241	0.0346	0.269	0.0453
181	U	0.673	0.2703	308	A	0.359	0.0891	0.595	0.0007
182	G	0.344	0.1418	309	G	0.049	0.0262	0.164	0.0240
183	A	0.514	0.2072	310	C	-	0.0000	0.147	0.0969
184	G	0.467	0.1942	311	U	-	0.0000	0.519	0.1011
185	A	0.227	0.0980	312	G	-	0.0000	0.253	0.1485
186	A	0.684	0.1663	313	G	0.976	0.0000	0.133	0.1032
187	G	0.462	0.1065	314	G	0.478	0.2659	0.744	0.0134
188	G	0.137	0.0766	315	G	0.547	0.0000	0.541	0.1513
189	C	0.176	0.1070	316	C	1.845	0.0000	-	0.0000
190	U	0.419	0.0774	317	C	1.224	0.7658	0.791	0.0000
191	U	0.085	0.0542	318	U	0.445	0.3118	0.686	0.0919
192	C	0.269	0.1220	319	U	0.133	0.1146	0.642	0.0184
193	C	0.534	0.0855	320	U	0.029	0.0000	0.23	0.1407
194	A	0.672	0.1607	321	C	0.114	0.01038	0.294	0.0269
195	C	0.347	0.1475	322	A	0.343	0.4207	0.377	0.0877
196	U	0.503	0.1700	323	A	0.296	0.1506	0.584	0.5289
197	A	0.407	0.0457	324	A	0.228	0.0827	0.427	0.0629
198	A	0.690	0.2003	325	A	0.119	0.1068	0.476	0.0849
199	G	0.467	0.0933	326	G	0.112	0.0290	0.254	0.1322

200	G	0.351	0.1162
201	C	0.015	0.0021
202	U	0.586	0.1434
203	A	0.309	0.0511
204	A	0.672	0.1293
205	C	0.294	0.1265
206	U	0.177	0.0815
207	C	0.750	0.3126
208	U	0.308	0.1330
209	C	0.485	0.2317
210	A	0.629	0.2320
211	A	0.721	0.1916
212	C	0.665	0.3253
213	A	0.778	0.2079
214	G	1.061	0.3167
215	A	0.543	0.1765
216	C	0.490	0.1538
217	A	1.335	0.1248
218	A	0.620	0.2375
219	C	0.723	0.2132
220	A	1.230	0.3716
221	A	0.605	0.2512
222	C	0.429	0.1524
223	A	0.917	0.2078
224	C	0.249	0.1297
225	C	0.602	0.0706
226	U	0.941	0.1176
227	G	0.198	0.0887
228	C	0.524	0.1573
229	U	0.907	0.1507
230	U	0.636	0.1707
231	C	0.658	0.2493
232	A	1.013	0.2409
233	U	0.492	0.1665
234	C	0.636	0.1493
235	A	0.694	0.0625
236	G	0.820	0.0956
237	C	0.425	0.0860
238	U	0.354	0.0951
239	G	0.379	0.1325
240	U	0.425	0.1273
241	U	0.319	0.1084
242	C	0.440	0.1036
243	C	0.430	0.0786
244	A	0.726	0.0502
245	G	1.303	0.3123
246	A	0.651	0.1737
247	G	0.606	0.1905
248	A	0.536	0.1061
249	A	0.423	0.1270

327	A	0.312	0.1428	0.332	0.1188
328	G			0.341	0.0212
329	A			0.467	0.1308
330	G			0.269	0.0750
331	C			0.05	0.0219
332	U			0.401	0.2758
333	U			0.347	0.1605
334	U			0.224	0.1428
335	G			0.203	0.0933
336	U			0.071	0.0474
337	A			0.654	0.0283
338	G			0.16	0.0481
339	A			0.207	0.1530
340	G			0.373	0.1810
341	G			0.442	0.1598
342	C			0.131	0.0990
343	A			-	0.0000
344	U			0.262	0.1803
345	U			-	0.0000
346	C			-	0.0000
347	U			0.448	0.3161
348	U			0.26	0.2998
349	U			0.515	0.1506
350	U			0.686	0.3203

250	C	0.230	0.0496
251	C	0.304	0.0916
252	C	-	0.0000
253	C	-	0.0000
254	C	-	0.0000
255	A	0.747	0.0443
256	U	0.398	0.0837
257	C	0.650	0.0606
258	A	0.262	0.0813
259	U	0.686	0.0410
260	G	0.243	0.0653
261	C	0.184	0.0527
262	C	0.306	0.1057
263	U	0.104	0.0815
264	C	0.496	0.0548
265	U	0.382	0.1223
266	C	0.382	0.0576
267	C	0.677	0.1639
268	U	0.292	0.0453
269	C	0.163	0.0315
270	A	0.671	0.0850
271	A	0.796	0.1007
272	C	0.252	0.0134
273	C	-	0.0000
274	U	-	0.0000
275	G	-	0.0000
276	C	0.319	0.0556
277	U	0.116	0.0610
278	U	0.634	0.1377
279	C	0.531	0.1239
280	A	0.368	0.1335
281	G	0.347	0.1598
282	U	0.499	0.1308
283	A	0.534	0.2298
284	C	0.121	0.1023
285	C	0.528	0.1108
286	A	0.156	0.0497
287	C	0.045	0.0576
288	C	0.412	0.0479
289	U	0.292	0.0892
290	C	0.418	0.0697
291	C	0.617	0.1342
292	A	1.337	0.1271
293	C	-	0.0000
294	A	-	0.0000
295	G	0.524	0.2586
296	A	1.095	0.1476
297	A	1.130	0.2815
298	U	0.488	0.1462
299	G	0.383	0.1130

300	G	-	0.0000
301	G	-	0.0000
302	C	0.326	0.1461
303	C	0.192	0.0246
304	G	0.061	0.0042
305	U	0.139	0.0516
306	A	0.525	0.0505
307	C	0.136	0.0368
308	C	0.152	0.0312
309	C	0.265	0.0128
310	A	0.683	0.0835
311	C	0.516	0.0708
312	A	0.583	0.0320
313	G	0.174	0.0395
314	C	0.291	0.1319
315	A	0.454	0.0814
316	G	0.420	0.0348
317	U	0.623	0.0398
318	G	0.328	0.0671
319	C	0.244	0.0550
320	A	0.459	0.0488
321	U	0.297	0.0397
322	G	0.398	0.1111
323	A	0.164	0.1476
324	U	0.175	0.0773
325	G	0.150	0.1512
326	A	0.358	0.1883
327	C	0.248	0.0436
328	C	0.513	0.1271
329	C	0.661	0.2944
330	A	0.694	0.1655
331	A	0.895	0.3313
332	A	0.784	0.2232
333	A	0.550	0.1299
334	C	0.287	0.1345
335	C	0.596	0.3099
336	A	0.734	0.1343
337	A	0.507	0.0758
338	G	0.298	0.0925
339	C	0.214	0.0851
340	C	0.339	0.0636
341	A	0.374	0.1884
342	A	0.675	0.1373
343	U	0.412	0.1485
344	C	0.181	0.0793
345	C	0.790	0.1187
346	A	0.881	0.4590
347	U	0.254	0.1871
348	C	0.427	0.0812
349	U	0.830	0.2215

350	G	0.352	0.1138
351	G	0.166	0.1049
352	U	0.365	0.0899
353	U	0.313	0.0724
354	G	0.412	0.0701
355	G	0.394	0.0968
356	U	0.431	0.4173
357	C	0.328	0.2483
358	A	0.599	0.1151
359	U	0.631	0.1670
360	U	0.127	0.1291
361	U	0.297	0.0697
362	U	0.183	0.1102
363	A	0.436	0.0564
364	C	0.183	0.0718
365	G	0.400	0.1061
366	G	0.324	0.1645
367	A	0.132	0.1253
368	C	0.153	0.0601
369	A	0.439	0.0387
370	C	0.225	0.3855
371	C	0.147	0.1430
372	C	0.462	0.1109
373	A	1.043	0.2611
374	U	0.514	0.2478
375	C	0.503	0.2055
376	U	0.647	0.1732
377	A	0.695	0.2559
378	U	0.973	0.1643
379	G	0.330	0.2419
380	A	0.316	0.1614
381	U	0.233	0.0937
382	U	0.198	0.1214
383	C	0.249	0.0574
384	C	0.406	0.0506
385	G	0.354	0.1623
386	U	0.329	0.1098
387	A	0.545	0.1752
388	U	0.430	0.1721
389	A	0.260	0.1749
390	C	0.433	0.0922
391	A	0.521	0.1561
392	C	0.277	0.0943
393	C	0.332	0.1273
394	U	0.527	0.2856
395	U	0.963	0.1646
396	A	0.773	0.3560
397	U	0.885	0.5280
398	C	1.617	0.2076
399	A	2.006	0.7353

400	A	1.324	0.6738
401	A	0.723	0.4296
402	U	0.412	0.2211
403	G	0.423	0.1602
404	U	0.183	0.1064
405	C	0.376	0.0216
406	G	0.495	0.0198
407	C	0.209	0.0462
408	C	0.235	0.0280
409	U	0.565	0.0875
410	A	0.724	0.0382
411	U	0.326	0.0580
412	G	0.384	0.0582
413	U	0.251	0.0632
414	A	0.642	0.0613
415	C	0.345	0.0273
416	U	0.309	0.0832
417	U	0.634	0.0480
418	U	0.334	0.0111
419	C	0.038	0.0671
420	C	0.464	0.0367
421	A	0.289	0.1202
422	C	0.441	0.0075
423	C	0.918	0.2786
424	U	0.490	0.3170
425	G	0.531	0.1128
426	G	0.628	0.2397
427	G	0.192	0.2959
428	C	0.471	0.1005
429	C	0.457	0.1754
430	A	0.381	0.0520
431	C	0.232	0.0949
432	A	0.763	0.0626
433	A	0.378	0.0942
434	U	0.393	0.0490
435	C	0.284	0.0966
436	A	0.561	0.0880
437	C	0.509	0.1169
438	A	0.831	0.0657
439	G	0.439	0.1322
440	U	0.049	0.0211
441	U	0.424	0.0269
442	U	0.506	0.0917
443	C	0.230	0.1262
444	C	0.267	0.1117
445	G	0.620	0.0481
446	C	0.437	0.0465
447	A	0.641	0.1107
448	G	0.092	0.0719
449	U	0.278	0.0919

450	A	0.690	0.0782
451	U	0.475	0.1029
452	C	0.140	0.0703
453	C	0.340	0.0590
454	A	1.000	0.0296
455	U	0.388	0.0438
456	C	0.882	0.0528
457	A	0.284	0.3345
458	U	0.544	0.0119
459	C	0.443	0.0726
460	A	0.748	0.0575
461	G	0.447	0.0869
462	U	0.099	0.0408
463	U	0.864	0.0341
464	G	0.293	0.0481
465	G	0.262	0.0335
466	A	0.617	0.0991
467	A	0.126	0.1223
468	C	0.544	0.0217
469	G	0.145	0.0809
470	C	0.218	0.0255
471	C	0.486	0.0265
472	U	0.373	0.0677
473	C	0.363	0.1299
474	U	0.577	0.0374
475	G	0.588	0.1080
476	A	0.600	0.0723
477	G	0.387	0.1857
478	C	0.310	0.0851
479	A	0.280	0.1426
480	C	0.298	0.0958
481	U	0.548	0.0474
482	C	0.316	0.0520
483	C	0.790	0.0651
484	A	0.580	0.1413
485	U	0.054	0.0235
486	C	0.428	0.0247
487	A	0.618	0.0771
488	C	0.304	0.0663
489	C	0.125	0.0629
490	U	-	0.0000
491	G	0.427	0.0989
492	A	0.666	0.1131
493	G	0.368	0.1359
494	U	0.104	0.0539
495	C	0.303	0.0549
496	A	0.642	0.0219
497	G	0.521	0.0542
498	G	0.544	0.3448
499	U	0.508	0.1980

500	A	0.415	0.0967
501	A	0.541	0.0556
502	U	0.316	0.0398
503	A	0.849	0.0220
504	C	0.428	0.1176
505	A	0.811	0.0995
506	U	0.201	0.0907
507	U	0.369	0.0866
508	U	0.325	0.0831
509	A	0.487	0.0784
510	C	0.290	0.0226
511	U	0.571	0.0266
512	G	0.319	0.1166
513	A	0.749	0.0719
514	U	0.571	0.0437
515	U	0.224	0.1109
516	C	0.319	0.0312
517	A	0.941	0.0459
518	U	0.230	0.0590
519	C	0.193	0.0919
520	C	0.361	0.0614
521	U	0.189	0.0447
522	C	0.334	0.0519
523	A	0.588	0.0595
524	G	0.782	0.0991
525	C	0.420	0.4449
526	G	0.776	0.0325
527	G	0.434	0.6640
528	A	0.499	0.1597
529	C	0.276	0.0560
530	U	0.397	0.0289
531	C	0.271	0.0755
532	U	0.536	0.0220
533	G	0.536	0.1045
534	A	0.416	0.0461
535	U	0.562	0.0376
536	A	0.540	0.1001
537	U	0.611	0.0692
538	G	0.601	0.0979
539	A	0.174	0.0622
540	C	0.343	0.1272
541	A	0.725	0.0989
542	U	0.117	0.1419
543	C	0.276	0.0476
544	C	0.410	0.0465
545	A	0.881	0.2284
546	C	1.324	0.3362
547	U	0.231	0.5367
548	A	1.160	0.0191
549	A	1.294	0.1379

550	A	1.164	0.1824
551	A	1.070	0.2404
552	A	1.102	0.3311
553	A	0.550	0.2077
554	U	0.714	0.1226
555	A	0.584	0.1612
556	U	0.608	0.0863
557	G	0.278	0.1570
558	U	0.419	0.1250
559	C	0.483	0.0933
560	A	0.261	0.1570
561	G	0.554	0.0519
562	A	0.248	0.1323
563	C	0.167	0.0589
564	C	0.618	0.0318
565	A	0.267	0.1352
566	C	0.171	0.1038
567	C	-	0.0000
568	A	0.739	0.0757
569	C	0.366	0.0693
570	C	0.238	0.0530
571	A	0.591	0.1577
572	A	0.829	0.0028
573	U	0.545	0.1153
574	G	0.681	0.1612
575	U	0.498	0.1384
576	U	0.473	0.0412
577	A	0.959	0.0104
578	A	0.574	0.2176
579	C	0.329	0.1157
580	C	0.375	0.0761
581	U	0.105	0.1262
582	C	0.431	0.0312
583	A	0.683	0.0958
584	C	0.380	0.1452
585	C	0.598	0.0085
586	U	0.603	0.1741
587	A	1.256	0.1020
588	A	0.593	0.3264
589	U	0.818	0.0491
590	G	1.070	0.2089
591	A	1.316	0.3141
592	C	0.396	0.2523
593	U	0.973	0.2210
594	U	0.731	0.2795
595	U	0.721	0.2204
596	C	0.396	0.0894
597	C	0.574	0.0338
598	A	0.819	0.1455
599	A	1.195	0.2606

600	A	1.069	0.7571
601	U	0.092	0.6082
602	U	0.575	0.0788
603	G	0.520	0.2920
604	G	0.354	0.2765
605	G	-	0.0000
606	U	1.037	0.2291
607	U	0.659	0.5688
608	A	0.500	0.0745
609	A	0.951	0.1513
610	A	1.126	0.2072
611	A	0.768	0.4544
612	C	0.453	0.3981
613	A	0.583	0.0911
614	U	0.767	0.0648
615	A	0.836	0.1731
616	C	0.666	0.1260
617	A	0.978	0.2327
618	U	0.640	0.3903
619	C	0.781	0.1635
620	A	1.296	0.1731
621	A	1.393	0.5720
622	A	1.077	0.6732
623	U	0.582	0.4745
624	U	0.479	0.2305
625	U	0.306	0.1278
626	U	0.384	0.1060
627	U	0.958	0.0366
628	A	1.270	0.2132
629	C	0.997	0.4115
630	A	0.628	0.1876
631	A	-	0.0000
632	A	1.199	0.0764
633	A	1.039	0.3321
634	C	0.498	0.3152
635	U	0.431	0.0896
636	C	0.641	0.1129
637	G	0.423	0.1485
638	A	1.173	0.1026
639	A	1.020	0.4384
640	U	0.791	0.2027
641	C	0.836	0.1724
642	U	0.684	0.1942
643	C	0.680	0.1152
644	G	0.410	0.1799
645	G	0.237	0.1025
646	U	0.395	0.0987
647	G	0.278	0.0986
648	G	0.248	0.1087
649	U	0.255	0.1097

650	A	0.770	0.1690
651	U	0.587	0.2925
652	U	0.907	0.1672
653	A	0.544	0.3588
654	U	0.244	0.1702
655	U	0.456	0.0914
656	C	0.138	0.1416
657	C	0.396	0.1200
658	G	0.198	0.1727
659	A	0.793	0.2123
660	C	0.511	0.2998
661	A	0.982	0.1695
662	G	0.876	0.5460
663	U	0.411	0.4097
664	A	0.470	0.1181
665	A	0.591	0.1588
666	A	0.348	0.2028
667	C	0.388	0.1348
668	G	0.359	0.1580
669	G	0.447	0.1247
670	A	0.835	0.1481
671	A	0.968	0.3201
672	A	0.392	0.2867
673	A	0.431	0.1266
674	C	0.244	0.1709
675	C	0.404	0.0912
676	C	0.335	0.1250
677	G	0.204	0.1466
678	U	0.903	0.2628
679	A	0.531	0.1336
680	C	0.582	0.3006
681	G	0.396	0.0982
682	U	0.238	0.1182
683	C	0.434	0.0464
684	A	0.332	0.1012
685	G	0.391	0.1193
686	A	0.211	0.0435
687	U	0.214	0.0730
688	C	0.162	0.0535
689	A	0.489	0.1024
690	C	0.165	0.0325
691	U	0.236	0.0569
692	G	0.289	0.0673
693	A	0.304	0.0811
694	U	0.324	0.0828
695	G	0.228	0.0329
696	A	0.482	0.0749
697	U	0.268	0.0705
698	G	0.409	0.1095
699	A	0.305	0.1189

700	A	0.491	0.1666
701	C	0.346	0.0894
702	U	0.163	0.1989
703	C	0.364	0.1797
704	A	0.394	0.1170
705	C	0.186	0.1816
706	C	0.370	0.0818
707	U	0.386	0.2291
708	U	0.341	0.0857
709	C	0.346	0.1074
710	U	0.415	0.1051
711	U	0.436	0.1639
712	G	0.328	0.1755
713	U	0.392	0.1569
714	A	0.463	0.1242
715	U	2.251	0.4906
716	A	0.568	0.1252
717	A	0.963	0.2020
718	C	0.316	0.0821
719	A	0.476	0.0283
720	C	0.360	0.0565
721	U	0.245	0.0581
722	U	0.567	0.0857
723	U	0.368	0.1737
724	U	0.485	0.1606
725	C	0.978	0.2020
726	A	0.726	0.4075
727	A	0.840	0.1117
728	A	0.707	0.0963
729	U	0.531	0.0862
730	A	0.705	0.0881
731	U	0.246	0.0549
732	U	0.631	0.1203
733	U	0.414	0.0782
734	G	0.196	0.1276
735	C	0.319	0.0782
736	U	0.621	0.1517
737	C	0.135	0.0671
738	C	0.306	0.0483
739	C	0.522	0.2666
740	U	0.865	0.2475
741	C	0.681	0.0474
742	U	0.824	0.2581
743	C	1.007	0.4342
744	A	0.723	0.3367
745	A	0.620	0.1859
746	U	0.634	0.2576
747	U	0.682	0.2761
748	C	0.173	0.1998
749	C	0.274	0.0662

750	U	0.647	0.1020
751	A	0.914	0.1833
752	C	0.188	0.0759
753	C	0.112	0.0770
754	U	0.422	0.1223
755	A	0.787	0.1708
756	C	0.226	0.1418
757	C	0.183	0.0495
758	U	0.681	0.3062
759	G	-	0.0000
760	G	1.315	0.3239
761	G	0.809	0.5331
762	U	0.518	0.1929
763	C	0.315	0.1317
764	A	0.936	0.1350
765	A	0.561	0.0229
766	A	0.552	0.0673
767	G	0.531	0.0726
768	A	0.218	0.0608
769	C	0.090	0.0095
770	A	0.580	0.0710
771	U	0.393	0.0251
772	C	0.416	0.0822
773	C	0.297	0.0864
774	U	0.363	0.0904
775	A	0.299	0.0296
776	U	0.575	0.1308
777	C	0.224	0.0344
778	C	0.291	0.0270
779	G	0.412	0.0996
780	U	0.302	0.0907
781	U	0.697	0.0962
782	G	0.853	0.0890
783	A	1.923	0.5346
784	U	1.291	0.2394
785	U	0.715	0.1339
786	A	0.585	0.0756
787	U	0.432	0.0743
788	A	0.358	0.0907
789	C	0.513	0.0789
790	G	0.393	0.0140
791	G	0.533	0.0896
792	A	0.275	0.0491
793	U	0.686	0.0639
794	A	-	0.0000
795	U	0.158	0.0490
796	C	0.424	0.0312
797	A	0.613	0.0427
798	U	0.626	0.0206
799	G	0.553	0.0268

800	A	0.816	0.1181
801	A	0.954	0.2079
802	A	0.651	0.0628
803	A	0.788	0.1084
804	U	0.400	0.0510
805	U	0.410	0.0486
806	C	0.192	0.1298
807	U	0.297	0.0186
808	U	0.321	0.0214
809	U	0.617	0.0757
810	C	0.625	0.1406
811	C	0.616	0.0863
812	A	0.855	0.0268
813	A	1.335	0.1201
814	A	1.324	0.1855
815	A	1.280	0.1424
816	G	0.462	0.0503
817	U	0.358	0.0856
818	A	1.060	0.4122
819	U	0.596	0.0374
820	U	0.900	0.0293
821	G	0.602	0.2000
822	A	0.997	0.1625
823	A	1.099	0.1443
824	A	1.062	0.1873
825	A	0.893	0.0638
826	A	0.857	0.0570
827	A	0.885	0.1316
828	U	0.817	0.1667
829	G	0.556	0.0638
830	C	0.530	0.1204
831	A	1.020	0.2722
832	A	0.771	0.1041
833	U	0.477	0.1030
834	C	0.409	0.1335
835	U	0.945	0.1669
836	G	0.804	0.2007
837	A	0.444	0.0481
838	U	0.637	0.1722
839	A	0.801	0.3879
840	C	0.418	0.4021
841	C	0.271	0.1598
842	C	0.526	0.1697
843	A	0.491	0.0762
844	A	0.716	0.1213
845	G	0.282	0.0777
846	A	0.712	0.1757
847	G	0.582	0.0915
848	G	0.728	0.0368
849	C	0.391	0.0637

850	A	0.544	0.0494
851	A	1.123	0.0767
852	A	0.606	0.0617
853	C	0.617	0.0886
854	G	0.534	0.1552
855	A	0.434	0.0490
856	C	0.211	0.0521
857	A	0.915	0.1000
858	U	0.550	0.0408
859	U	1.254	0.0148
860	G	0.290	0.0471
861	U	0.346	0.0715
862	G	0.386	0.0802
863	A	0.681	0.1025
864	C	0.153	0.0076
865	C	0.208	0.0395
866	C	0.798	0.2079
867	U	-	0.0000
868	G	-	0.0000
869	G	-	0.0000
870	C	-	0.0000
871	A	0.936	0.1197
872	A	0.946	0.0890
873	A	0.589	0.2451
874	U	0.536	0.1731
875	U	0.554	0.1511
876	U	0.533	0.1151
877	G	0.493	0.1192
878	C	0.333	0.1136
879	A	0.766	0.0864
880	A	0.995	0.0286
881	U	0.651	0.0539
882	A	1.471	0.1909
883	U	0.396	0.0973
884	A	1.136	0.0912
885	A	0.915	0.1166
886	U	0.739	0.0417
887	G	0.555	0.0743
888	G	0.141	0.0327
889	C	0.529	0.1287
890	A	0.440	0.0868
891	G	0.487	0.0881
892	U	1.059	0.2909
893	A	0.307	0.1713
894	C	0.329	0.0644
895	A	0.569	0.0728
896	C	0.224	0.0732
897	C	0.289	0.0913
898	U	0.364	0.0294
899	G	0.118	0.0299

900	C	0.181	0.0452
901	A	0.589	0.0959
902	G	0.341	0.0722
903	A	0.452	0.0726
904	U	0.479	0.0323
905	G	0.254	0.0810
906	C	0.334	0.0444
907	A	0.224	0.0774
908	U	0.316	0.0710
909	U	0.210	0.0405
910	U	0.567	0.0537
911	G	0.356	0.0431
912	A	0.577	0.0956
913	A	0.831	0.1160
914	A	0.962	0.0604
915	C	0.431	0.0466
916	A	0.660	0.2075
917	A	1.107	0.1110
918	A	0.678	0.1354
919	A	0.870	0.1036
920	G	0.427	0.1005
921	U	0.372	0.0632
922	C	0.451	0.0952
923	A	0.687	0.1363
924	C	0.381	0.0817
925	A	0.643	0.1572
926	A	0.830	0.1831
927	A	0.781	0.1215
928	C	0.365	0.0520
929	A	0.891	0.1126
930	U	0.093	0.0021
931	U	0.309	0.0686
932	A	0.906	0.0495
933	U	0.225	0.1032
934	C	0.403	0.0897
935	G	0.275	0.0675
936	A	0.473	0.0779
937	C	0.267	0.0045
938	A	0.545	0.0905
939	G	0.372	0.0666
940	A	0.366	0.1115
941	C	0.237	0.0320
942	U	0.273	0.0318
943	G	0.267	0.0236
944	A	0.375	0.0438
945	A	0.400	0.0423
946	C	0.240	0.0218
947	A	0.484	0.0485
948	A	0.485	0.0531
949	U	0.280	0.1040

950	A	0.621	0.0727
951	A	0.411	0.0575
952	U	0.417	0.0465
953	G	0.360	0.0090
954	G	0.160	0.0430
955	C	0.246	0.0862
956	A	0.514	0.1115
957	U	0.383	0.0662
958	U	0.310	0.0336
959	C	0.415	0.0472
960	A	0.294	0.0465
961	U	0.254	0.0307
962	A	0.464	0.0434
963	U	0.351	0.0840
964	C	0.313	0.1069
965	A	0.575	0.0817
966	A	0.452	0.0399
967	U	0.293	0.1186
968	A	0.576	0.1293
969	A	0.480	0.0849
970	C	0.227	0.1253
971	A	0.462	0.1607
972	A	0.340	0.0255
973	G	0.276	0.0163
974	G	0.202	0.0303
975	U	0.186	0.0353
976	C	0.284	0.1291
977	G	0.156	0.0354
978	C	0.178	0.0267
979	A	0.220	0.0587
980	U	0.310	0.1117
981	G	0.200	0.2071
982	C	1.090	0.5089
983	C	0.877	0.1920
984	A	0.583	0.1351
985	A	0.423	0.0170
986	U	0.265	0.0396
987	U	0.280	0.0276
988	A	0.670	0.2164
989	A	0.602	0.0460
990	U	0.424	0.0820
991	U	0.471	0.1294
992	A	0.540	0.3988
993	U	0.387	0.3316
994	G	0.386	0.4731
995	A	0.200	0.1888
996	G	0.321	0.5218
997	A	0.237	0.1994
998	G	0.396	0.0555
999	G	0.107	0.0304

1000	U	0.069	0.1342
1001	C	0.225	0.4510
1002	U	1.242	0.4179
1003	A	0.262	0.1963
1004	U	0.036	0.2949
1005	C	0.108	0.2668
1006	U	0.185	0.0838
1007	G	0.342	0.5202
1008	G	1.315	0.5305
1009	C	0.266	0.1080
1010	G	0.097	0.1260
1011	A	0.060	0.0770
1012	A	0.152	0.1042
1013	U	0.088	0.1940
1014	A	0.084	0.1823
1015	U	0.122	0.0225
1016	A	0.153	0.2165
1017	A	0.627	0.3174
1018	A	0.830	0.3728
1019	U	0.589	0.3718
1020	U	0.457	0.4907
1021	U	0.611	0.4914
1022	U	0.269	0.2143
1023	U	0.591	0.4879
1024	A	0.510	0.2489
1025	C	0.547	0.2242
1026	G	0.074	0.0813
1027	C	0.353	0.1527
1028	U	0.623	0.5353
1029	A	0.884	0.4052
1030	C	0.612	0.3726
1031	A	0.226	0.1541
1032	C	1.074	0.7863
1033	A	0.632	0.4426
1034	C	0.633	0.3882
1035	G	0.413	0.0771
1036	U	0.111	0.0516
1037	C	0.046	0.0325
1038	A	0.489	0.0615
1039	U	0.088	0.0269
1040	C	0.273	0.0926
1041	G	0.549	0.1004
1042	A	0.247	0.1082
1043	C	2.110	0.8591
1044	A	0.841	0.3041
1045	U	0.165	0.0509
1046	C	0.091	0.0587
1047	U	0.243	0.0983
1048	A	0.480	0.1584
1049	A	0.552	0.2708

1050	A	0.273	0.1329
1051	U	0.307	0.1259
1052	A	0.364	0.1054
1053	U	0.428	0.1435
1054	G	0.016	0.0156
1055	A	0.512	0.1916
1056	C	0.083	0.0283
1057	A	0.300	0.0445
1058	G	0.238	0.0665
1059	U	0.197	0.0933
1060	C	0.947	0.4172
1061	G	0.264	0.1174
1062	C	0.461	0.2737
1063	U	0.122	0.1230
1064	G	0.251	0.1075
1065	A	0.283	0.2539
1066	A	0.763	0.1761
1067	C	0.850	0.2737
1068	U	-	0.0000
1069	G	-	0.0000
1070	U	-	0.0000
1071	U	0.043	0.0134
1072	C	0.229	0.1831
1073	U	0.289	0.0728
1074	U	0.450	0.2744
1075	A	0.662	0.3210
1076	G	0.440	0.2263
1077	A	0.173	0.0877
1078	U	0.185	0.1301
1079	A	0.515	0.2369
1080	U	0.132	0.0544
1081	C	-	0.0000
1082	C	-	0.0000
1083	A	0.335	0.1435
1084	U	0.242	0.1577
1085	G	0.308	0.0750
1086	C	0.863	0.1147
1087	U	0.277	0.0387
1088	A	0.709	0.0894
1089	U	0.184	0.0906
1090	U	0.070	0.0106
1091	U	0.501	0.2217
1092	A	0.443	0.0983
1093	U	0.256	0.0709
1094	G	0.285	0.1603
1095	A	0.699	0.1016
1096	A	0.410	0.1286
1097	G	0.498	0.0765
1098	A	0.597	0.0848
1099	A	0.156	0.0347

1100	C	0.195	0.0432
1101	A	0.230	0.0185
1102	A	0.204	0.0327
1103	C	0.147	0.0535
1104	A	0.378	0.0590
1105	G	0.911	0.1814
1106	G	1.059	0.2382
1107	G	4.097	0.7250
1108	A	2.059	0.2021
1109	U	0.116	0.0834
1110	C	0.610	0.0512
1111	G	0.213	0.0820
1112	A	2.027	0.1397
1113	G	0.497	0.2082
1114	A	0.348	0.0579
1115	A	0.263	0.0876
1116	A	1.212	0.3199
1117	C	2.063	0.2494
1118	A	1.062	0.0699
1119	G	0.682	0.1645
1120	U	0.323	0.0446
1121	A	0.054	0.0057
1122	A	0.382	0.1216
1123	A	0.330	0.0305
1124	C	0.242	0.0155
1125	C	0.668	0.1076
1126	U	0.424	0.2703
1127	A	0.630	0.1646
1128	A	0.105	0.0875
1129	U	0.157	0.0266
1130	U	0.564	0.2212
1131	A	0.252	0.1253
1132	C	0.084	0.0495
1133	A	0.449	0.1370
1134	G	2.144	0.2484
1135	G	1.955	0.5857
1136	A	2.136	0.3644
1137	G	0.750	0.1781
1138	A	0.123	0.0315
1139	A	0.289	0.0834
1140	A	0.106	0.0150
1141	U	0.202	0.0216
1142	C	0.059	0.0511
1143	C	0.476	0.2662
1144	G	0.285	0.1881
1145	A	1.357	0.3145
1146	G	0.817	0.2609
1147	U	0.688	0.1833
1148	G	0.819	0.3935
1149	A	-	0.0000

1150	U	-	0.0000
1151	G	0.814	0.4130
1152	A	0.280	0.1626
1153	G	0.234	0.1464
1154	A	0.337	0.0704
1155	A	0.321	0.2061
1156	G	0.186	0.1767
1157	A	0.461	0.0495
1158	A	0.403	0.0482
1159	U	0.454	0.2602
1160	G	0.228	0.0631
1161	A	0.238	0.1358
1162	U	0.128	0.0650
1163	U	0.150	0.0984
1164	C	0.189	0.1075
1165	U	0.207	0.0699
1166	C	0.156	0.0812
1167	G	0.213	0.0968
1168	C	0.246	0.0529
1169	A	0.247	0.1872
1170	G	0.160	0.1279
1171	C	0.226	0.0463
1172	U	0.188	0.1180
1173	A	0.334	0.3157
1174	U	0.865	0.9436
1175	A	1.673	0.8695
1176	C	1.438	0.7701
1177	G	1.110	0.3209
1178	A	1.069	0.0926
1179	A	0.807	0.1887
1180	U	0.614	0.4658
1181	A	0.299	0.0778
1182	C	0.405	0.2593
1183	A	0.617	0.0759
1184	A	0.498	0.1762
1185	C	0.559	0.3587
1186	C	0.686	0.0228
1187	A	0.578	0.1741
1188	A	0.704	0.0762
1189	A	0.544	0.1328
1190	C	0.267	0.0411
1191	C	0.234	0.0306
1192	C	0.362	0.2832
1193	A	0.900	0.0856
1194	A	1.037	0.1675
1195	A	1.008	0.3160
1196	G	0.534	0.2729
1197	U	0.461	0.1343
1198	U	0.478	0.2261
1199	A	0.370	0.1301

1200	U	0.506	0.1279
1201	A	0.343	0.2661
1202	G	0.107	0.0748
1203	C	0.144	0.1195
1204	U	0.327	0.0540
1205	C	0.348	0.0963
1206	G	0.313	0.1595
1207	G	0.263	0.0779
1208	A	0.191	0.0757
1209	A	0.232	0.1602
1210	U	0.049	0.7814
1211	C	0.461	0.5381
1212	C	0.353	0.1546
1213	U	0.560	0.3474
1214	C	0.156	0.0599
1215	A	0.429	0.0866
1216	A	0.575	0.1844
1217	A	0.712	0.1368
1218	A	1.015	0.0580
1219	A	0.842	0.2771
1220	A	0.474	0.0356
1221	C	0.470	0.0421
1222	A	0.546	0.1022
1223	A	0.651	0.1316
1224	A	0.561	0.2359
1225	U	0.884	0.2963
1226	A	0.554	0.1768
1227	A	0.669	0.0959
1228	U	0.480	0.0721
1229	U	0.428	0.0749
1230	C	0.287	0.1699
1231	G	0.485	0.1383
1232	A	0.458	0.2672
1233	A	0.781	0.0115
1234	A	0.447	0.1300
1235	U	0.299	0.0940
1236	C	0.381	0.1658
1237	G	0.394	0.1222
1238	A	0.411	0.1869
1239	A	0.599	0.0404
1240	A	0.661	0.0410
1241	A	0.418	0.0637
1242	C	0.378	0.0538
1243	A	0.907	0.2035
1244	G	0.046	0.0370
1245	C	0.180	0.2503
1246	C	0.423	0.0966
1247	A	0.452	0.1289
1248	G	0.565	0.2786
1249	G	0.327	0.0661

1250	G	-	0.0000
1251	C	0.075	0.0376
1252	U	0.293	0.0796
1253	C	0.399	0.0790
1254	A	0.380	0.1012
1255	C	0.174	0.0784
1256	A	0.701	0.0615
1257	A	0.612	0.0095
1258	U	0.516	0.0316
1259	G	0.140	0.1152
1260	U	0.162	0.0263
1261	A	0.658	0.0895
1262	U	0.283	0.0373
1263	C	0.145	0.0730
1264	C	0.526	0.1038
1265	A	0.183	0.0711
1266	C	-	0.0000
1267	A	0.677	0.2767
1268	U	0.309	0.0810
1269	C	0.412	0.1131
1270	U	0.776	0.0332
1271	A	1.298	0.0191
1272	A	0.702	0.0785
1273	U	0.790	0.0399
1274	A	0.796	0.0679
1275	A	0.972	0.1909
1276	C	0.114	0.0122
1277	U	0.248	0.0609
1278	C	0.274	0.0721
1279	U	0.335	0.0907
1280	C	0.152	0.0176
1281	C	0.225	0.0814
1282	C	0.851	0.0780
1283	A	0.551	0.0344
1284	G	0.163	0.0465
1285	C	0.367	0.0545
1286	A	0.919	0.0330
1287	C	0.291	0.1378
1288	G	-	0.0000
1289	G	0.696	0.0637
1290	A	0.140	0.0223
1291	C	0.309	0.0365
1292	A	0.872	0.0423
1293	A	0.905	0.0676
1294	C	0.802	0.1738
1295	G	0.559	0.0701
1296	A	0.550	0.0384
1297	U	0.481	0.0268
1298	U	0.502	0.0330
1299	C	0.069	0.0214

1300	C	0.444	0.0711
1301	A	0.582	0.0455
1302	U	2.088	0.0898
1303	C	2.496	1.3397
1304	A	2.509	1.1175
1305	G	0.797	0.5394
1306	U	0.429	0.2107
1307	A	0.438	0.1453
1308	A	0.738	0.1507
1309	A	1.086	0.1792
1310	U	0.123	0.0501
1311	C	0.591	0.0949
1312	A	0.932	0.0662
1313	A	0.232	0.0174
1314	C	0.553	0.0557
1315	U	0.259	0.0594
1316	A	0.717	0.0499
1317	C	0.328	0.0972
1318	U	0.321	0.0100
1319	G	0.385	0.2141
1320	A	0.380	0.0355
1321	A	0.597	0.0250
1322	C	0.252	0.0420
1323	C	0.503	0.0783
1324	G	0.208	0.0167
1325	A	0.683	0.0725
1326	U	0.523	0.0563
1327	U	0.302	0.0669
1328	C	0.427	0.0527
1329	A	0.397	0.1105
1330	A	0.560	0.0730
1331	U	0.629	0.1308
1332	U	0.614	0.1171
1333	G	0.668	0.1293
1334	A	0.856	0.1172
1335	A	0.927	0.0201
1336	C	0.596	0.1100
1337	A	0.727	0.0800
1338	A	0.794	0.0901
1339	U	0.385	0.0437
1340	A	1.097	0.0438
1341	A	0.423	0.0823
1342	G	0.105	0.0456
1343	C	0.463	0.0647
1344	A	0.455	0.1477
1345	C	0.309	0.0318
1346	G	0.190	0.0562
1347	A	0.531	0.0596
1348	C	0.237	0.0051
1349	C	0.266	0.0368

1350	U	0.639	0.0442
1351	U	0.349	0.0741
1352	C	0.708	0.1510
1353	A	1.149	0.1110
1354	U	0.553	0.1398
1355	C	0.367	0.1591
1356	U	0.327	0.1570
1357	U	0.241	0.1188
1358	A	0.353	0.1295
1359	G	0.293	0.1358
1360	G	0.122	0.0340
1361	C	0.263	0.0601
1362	C	0.243	0.0261
1363	A	0.816	0.0976
1364	G	0.289	0.0547
1365	A	0.569	0.0892
1366	A	0.657	0.0770
1367	A	0.568	0.1160
1368	C	0.081	0.0375
1369	U	0.274	0.0423
1370	U	0.186	0.0240
1371	A	0.474	0.0871
1372	C	0.205	0.0437
1373	U	0.415	0.0930
1374	G	0.275	0.1097
1375	A	0.336	0.0605
1376	A	0.680	0.1523
1377	U	0.192	0.0340
1378	C	0.074	0.0265
1379	U	0.225	0.0191
1380	A	0.449	0.0507
1381	C	0.256	0.0183
1382	A	0.344	0.0772
1383	G	0.290	0.0684
1384	U	0.342	0.0605
1385	A	0.370	0.0528
1386	A	0.626	0.0960
1387	A	0.601	0.0941
1388	U	0.353	0.0427
1389	C	0.321	0.0741
1390	A	0.760	0.1592
1391	U	0.465	0.0179
1392	A	0.707	0.1420
1393	C	0.333	0.0243
1394	U	0.326	0.0457
1395	A	0.924	0.1830
1396	A	0.760	0.1334
1397	U	0.353	0.0691
1398	C	0.327	0.0785
1399	A	0.588	0.1288

1400	U	0.356	0.0422
1401	U	0.396	0.0718
1402	C	0.155	0.0628
1403	U	0.287	0.0304
1404	G	0.306	0.0584
1405	A	0.262	0.0476
1406	U	0.296	0.0456
1407	G	0.170	0.0689
1408	A	0.493	0.1046
1409	U	0.249	0.0644
1410	G	0.484	0.1257
1411	A	0.387	0.0841
1412	A	0.338	0.1350
1413	C	0.146	0.0438
1414	U	0.266	0.0907
1415	C	0.167	0.0533
1416	C	0.116	0.0261
1417	C	0.735	0.4039
1418	U	0.596	0.2579
1419	G	0.406	0.1357
1420	G	0.285	0.1102
1421	A	0.152	0.0331
1422	C	0.257	0.1171
1423	A	0.331	0.1408
1424	C	0.127	0.0346
1425	C	0.209	0.1042
1426	U	0.253	0.1382
1427	C	0.132	0.1069
1428	C	0.243	0.0880
1429	U	0.197	0.0607
1430	U	0.177	0.0443
1431	C	0.172	0.0776
1432	U	0.217	0.0477
1433	C	0.334	0.0850
1434	G	0.133	0.0205
1435	A	0.521	0.0514
1436	U	0.276	0.0514
1437	U	0.194	0.0563
1438	C	0.230	0.0835
1439	A	0.425	0.1430
1440	G	0.259	0.0913
1441	G	0.152	0.0906
1442	A	0.390	0.0804
1443	G	0.068	0.0449
1444	C	0.280	0.0873
1445	A	0.274	0.0681
1446	U	0.201	0.0994
1447	C	-	0.0000
1448	A	0.773	0.2968
1449	C	0.155	0.0467

1450	G	0.376	0.0690
1451	A	0.200	0.0740
1452	A	0.439	0.0490
1453	C	0.207	0.0582
1454	C	0.119	0.0204
1455	C	0.261	0.4653
1456	U	0.440	0.1294
1457	U	0.368	0.1006
1458	A	0.547	0.1480
1459	U	0.505	0.0547
1460	A	0.804	0.0980
1461	A	0.517	0.1063
1462	G	0.350	0.0428
1463	A	0.435	0.0349
1464	U	0.207	0.0306
1465	C	0.256	0.0272
1466	U	0.369	0.0460
1467	G	0.216	0.0283
1468	C	0.247	0.0673
1469	U	0.149	0.0354
1470	C	0.389	0.1378
1471	A	0.338	0.0653
1472	U	0.215	0.0334
1473	C	0.258	0.0822
1474	A	0.577	0.1333
1475	C	0.337	0.1547
1476	A	0.672	0.0658
1477	U	0.299	0.0285
1478	A	0.641	0.0944
1479	C	0.518	0.0889
1480	A	0.469	0.1132
1481	C	0.281	0.1260
1482	U	0.591	0.1611
1483	C	0.208	0.1316
1484	A	0.507	0.0945
1485	G	0.166	0.0296
1486	C	0.215	0.0832
1487	A	0.406	0.0395
1488	U	0.102	0.0669
1489	C	0.262	0.0519
1490	A	0.494	0.0901
1491	U	0.273	0.0668
1492	C	0.313	0.0716
1493	U	0.404	0.1239
1494	A	0.215	0.0642
1495	A	0.680	0.1027
1496	U	0.241	0.0749
1497	C	0.243	0.0596
1498	C	0.239	0.0579
1499	U	0.368	0.0504

1500	G	0.543	0.0493
1501	A	1.129	0.0328
1502	C	0.575	0.1322
1503	A	1.385	0.1141
1504	U	0.366	0.0390
1505	A	0.611	0.0737
1506	A	0.729	0.0398
1507	A	0.652	0.0632
1508	C	0.174	0.0488
1509	G	0.420	0.1026
1510	U	0.260	0.1070
1511	A	0.442	0.1044
1512	G	0.281	0.0874
1513	U	0.170	0.0473
1514	U	0.261	0.0471
1515	G	0.172	0.0169
1516	A	0.180	0.0440
1517	U	0.220	0.0222
1518	G	0.183	0.0336
1519	C	0.231	0.0641
1520	U	0.544	0.1691
1521	C	0.088	0.0664
1522	A	0.695	0.1197
1523	A	0.614	0.1192
1524	A	0.527	0.0956
1525	A	0.582	0.0954
1526	A	0.907	0.0449
1527	A	0.757	0.1085
1528	G	0.703	0.0551
1529	A	0.725	0.0965
1530	A	1.196	0.1091
1531	A	0.795	0.0693
1532	U	0.538	0.0247
1533	A	0.576	0.0718
1534	U	0.662	0.0514
1535	A	0.565	0.0185
1536	C	0.203	0.0406
1537	C	0.364	0.0458
1538	A	0.627	0.0538
1539	A	0.766	0.0790
1540	U	0.326	0.0987
1541	U	0.748	0.0670
1542	A	0.750	0.1010
1543	A	0.840	0.0782
1544	C	0.555	0.0495
1545	G	0.417	0.0955
1546	C	0.357	0.0624
1547	U	0.492	0.0804
1548	A	0.477	0.1343
1549	U	0.508	0.0817

1550	U	0.355	0.0785
1551	G	0.444	0.0625
1552	G	0.184	0.1088
1553	U	0.234	0.0996
1554	G	0.274	0.0790
1555	A	0.574	0.0679
1556	C	0.172	0.0540
1557	C	0.332	0.0615
1558	U	0.849	0.2205
1559	A	0.881	0.2024
1560	C	0.569	0.1612
1561	A	1.151	0.2221
1562	A	0.874	0.0655
1563	U	0.869	0.1020
1564	U	0.431	0.0370
1565	U	0.971	0.0899
1566	C	1.108	0.1830
1567	A	0.456	0.2450
1568	C	0.295	0.1385
1569	U	0.330	0.1538
1570	U	0.567	0.1056
1571	C	0.316	0.0824
1572	C	0.437	0.0686
1573	A	0.677	0.0930
1574	G	0.431	0.0455
1575	G	0.500	0.0761
1576	A	0.253	0.0709
1577	C	0.664	0.1517
1578	A	0.321	0.0254
1579	A	0.315	0.0768
1580	C	0.361	0.0372
1581	A	0.601	0.0956
1582	C	0.398	0.1205
1583	C	0.672	0.0731
1584	A	0.534	0.0442
1585	A	0.780	0.0476
1586	A	1.042	0.0940
1587	A	0.570	0.0952
1588	C	0.610	0.1198
1589	A	0.803	0.1214
1590	U	0.702	0.0917
1591	C	0.644	0.0598
1592	A	1.005	0.0869
1593	A	1.197	0.1857
1594	U	0.798	0.0587
1595	A	0.956	0.0727
1596	A	0.962	0.0499
1597	A	0.919	0.0806
1598	G	0.411	0.1193
1599	G	0.320	0.0695

1600	U	0.341	0.0657
1601	A	0.393	0.0729
1602	U	0.614	0.0216
1603	U	0.423	0.0660
1604	G	0.637	0.0421
1605	C	0.418	0.0237
1606	A	0.497	0.1174
1607	C	0.416	0.0360
1608	A	0.344	0.1070
1609	C	0.524	0.0658
1610	U	0.403	0.0474
1611	C	0.526	0.0459
1612	C	0.414	0.0476
1613	U	0.721	0.0570
1614	A	1.177	0.1048
1615	A	1.172	0.0767
1616	C	0.797	0.1297
1617	A	0.879	0.0480
1618	U	0.834	0.0574
1619	A	0.834	0.0265
1620	G	0.659	0.0502
1621	C	0.319	0.0474
1622	C	0.328	0.0402
1623	U	0.862	0.0739
1624	A	0.315	0.0399
1625	U	0.725	0.0367
1626	G	0.577	0.0444
1627	A	0.548	0.0679
1628	C	0.089	0.0471
1629	U	0.628	0.0347
1630	U	0.590	0.0746
1631	A	0.843	0.0132
1632	C	0.143	0.0418
1633	U	0.476	0.0753
1634	C	0.693	0.0931
1635	A	0.960	0.1176
1636	G	0.429	0.0971
1637	U	0.424	0.0623
1638	U	0.340	0.0282
1639	U	0.876	0.0677
1640	G	0.680	0.1143
1641	A	1.089	0.1038
1642	A	1.172	0.1037
1643	U	0.908	0.0727
1644	G	0.879	0.0918
1645	A	0.912	0.1396
1646	A	0.970	0.1537
1647	U	0.452	0.0882
1648	U	0.439	0.0834
1649	G	0.631	0.1501

1650	G	0.239	0.0825
1651	C	0.384	0.0774
1652	U	0.456	0.0778
1653	G	0.702	0.0699
1654	C	0.623	0.1288
1655	A	0.710	0.0559
1656	G	0.606	0.0622
1657	U	0.571	0.0214
1658	A	1.156	0.0460
1659	G	0.914	0.1204
1660	A	0.760	0.0633
1661	U	1.132	0.0674
1662	A	1.162	0.0313
1663	U	0.462	0.0500
1664	C	0.675	0.1158
1665	A	0.845	0.0344
1666	C	0.686	0.0451
1667	A	0.936	0.1241
1668	G	0.542	0.0703
1669	C	0.325	0.0298
1670	A	0.645	0.0331
1671	U	0.819	0.0519
1672	G	0.502	0.0503
1673	C	0.346	0.0866
1674	U	0.053	0.0267
1675	U	0.405	0.0713
1676	U	0.623	0.0710
1677	A	0.753	0.1005
1678	C	0.549	0.1153
1679	C	0.494	0.0522
1680	A	1.231	0.3263
1681	A	1.148	0.2333
1682	A	0.860	0.2392
1683	A	1.144	0.3638
1684	A	0.349	0.1128
1685	C	0.419	0.1538
1686	G	0.830	0.2984
1687	U	0.378	0.1215
1688	C	0.301	0.0754
1689	U	0.522	0.2181
1690	U	1.211	0.4297
1691	A	0.916	0.3454
1692	G	0.683	0.1498
1693	A	0.514	0.1861
1694	A	0.701	0.2044
1695	C	0.326	0.0954
1696	G	0.391	0.1638
1697	G	0.266	0.0548
1698	U	0.473	0.1923
1699	C	0.193	0.1220

1700	U	0.383	0.1033
1701	G	0.487	0.2000
1702	A	0.424	0.0690
1703	C	0.560	0.2830
1704	G	0.333	0.1317
1705	G	0.166	0.0343
1706	C	0.230	0.0316
1707	A	0.567	0.3968
1708	C	0.337	0.2134
1709	U	0.311	0.0893
1710	G	0.322	0.1244
1711	U	0.166	0.0268
1712	A	0.420	0.1713
1713	C	0.175	0.0632
1714	U	0.402	0.1731
1715	U	0.379	0.1090
1716	G	0.207	0.0874
1717	C	0.404	1.3426
1718	A	0.320	0.1839
1719	C	0.202	0.0367
1720	C	0.100	0.0195
1721	U	0.187	0.0322
1722	A	0.674	0.2010
1723	U	0.458	0.1724
1724	C	0.428	0.1190
1725	G	0.371	0.1290
1726	U	0.254	0.1143
1727	A	0.585	0.2337
1728	A	0.563	0.1716
1729	A	0.493	0.0690
1730	A	0.389	0.0775
1731	U	0.187	0.0488
1732	A	0.613	0.0843
1733	U	0.377	0.1513
1734	G	0.291	0.1135
1735	G	0.189	0.0715
1736	A	0.494	0.1383
1737	G	0.149	0.0581
1738	A	0.286	0.0466
1739	C	0.198	0.0519
1740	U	0.315	0.1155
1741	U	0.261	0.3320
1742	U	0.288	0.1030
1743	U	0.231	0.0646
1744	A	0.447	0.1348
1745	C	0.070	0.0342
1746	U	0.441	0.2004
1747	G	0.298	0.0875
1748	G	0.196	0.1083
1749	G	0.116	0.0363

1750	U	0.201	0.0776
1751	A	0.544	0.1366
1752	U	0.213	0.0688
1753	C	0.384	0.1502
1754	U	0.560	0.1333
1755	A	0.660	0.3224
1756	A	0.600	0.2716
1757	A	0.756	0.3113
1758	A	0.823	0.2960
1759	A	0.311	0.1227
1760	G	0.404	0.1893
1761	U	0.258	0.1370
1762	A	0.608	0.1153
1763	C	0.165	0.0537
1764	U	0.401	0.1183
1765	U	0.582	0.1977
1766	G	0.200	0.0915
1767	C	0.312	0.1927
1768	U	0.441	0.2819
1769	U	0.489	0.2171
1770	C	0.154	0.1070
1771	C	0.444	0.1090
1772	A	0.735	0.1467
1773	U	0.284	0.0627
1774	C	0.440	0.1192
1775	A	0.659	0.1362
1776	A	1.024	0.1607
1777	A	0.343	0.0208
1778	U	0.724	0.1953
1779	A	0.681	0.1390
1780	U	0.045	0.0304
1781	C	0.126	0.0184
1782	U	0.393	0.0558
1783	C	0.223	0.0749
1784	C	0.312	0.1606
1785	G	0.193	0.1399
1786	U	-	0.0000
1787	A	0.441	0.2076
1788	C	0.070	0.0490
1789	C	0.080	0.0318
1790	C	0.405	0.2597
1791	A	0.519	0.1493
1792	C	0.084	0.0176
1793	C	0.376	0.0764
1794	A	0.770	0.0861
1795	U	0.439	0.1757
1796	C	0.618	0.1950
1797	A	1.118	0.3024
1798	A	1.050	0.3376
1799	U	0.658	0.1565

1800	A	1.202	0.4987
1801	A	0.565	0.2306
1802	U	0.375	0.1904
1803	G	0.380	0.0859
1804	U	0.186	0.0650
1805	C	0.199	0.0454
1806	C	0.211	0.0401
1807	A	0.582	0.1237
1808	U	0.529	0.1491
1809	A	0.297	0.1895
1810	C	0.252	0.0989
1811	A	0.371	0.1692
1812	A	0.512	0.1029
1813	G	0.397	0.0538
1814	U	0.328	0.0277
1815	G	0.132	0.0625
1816	A	0.396	0.0587
1817	A	0.609	0.0768
1818	A	0.292	0.0252
1819	G	0.307	0.0314
1820	U	0.139	0.0075
1821	A	0.424	0.0260
1822	C	0.178	0.0417
1823	A	0.423	0.0183
1824	C	0.459	0.0254
1825	G	0.381	0.0792
1826	C	0.362	0.1562
1827	A	0.828	0.3467
1828	A	0.929	0.0790
1829	A	0.523	0.0949
1830	U	0.503	0.0644
1831	A	0.778	0.0206
1832	U	0.594	0.0762
1833	C	0.322	0.0101
1834	C	0.498	0.0118
1835	U	0.291	0.0660
1836	U	0.474	0.0842
1837	A	0.768	0.0471
1838	U	0.464	0.0916
1839	C	0.387	0.0820
1840	C	0.252	0.0991
1841	U	0.553	0.0982
1842	U	0.437	0.0910
1843	U	0.639	0.0376
1844	C	0.434	0.2157
1845	A	0.668	0.1086
1846	U	0.693	0.1737
1847	U	0.119	0.0505
1848	C	0.422	0.0287
1849	A	0.559	0.0250

1850	U	0.466	0.1007
1851	C	0.579	0.1354
1852	G	0.380	0.0320
1853	A	0.361	0.0362
1854	A	-	0.0000
1855	U	1.006	0.1987
1856	G	0.193	0.0552
1857	C	0.129	0.1028
1858	U	0.340	0.1010
1859	U	0.418	0.1129
1860	G	0.420	0.1310
1861	C	0.442	0.1005
1862	G	0.215	0.0692
1863	C	0.444	0.0286
1864	A	0.237	0.0656
1865	U	0.694	0.1988
1866	G	0.516	0.1125
1867	C	0.353	0.0173
1868	C	0.593	0.0656
1869	A	0.331	0.0748
1870	A	0.602	0.1464
1871	U	0.376	0.0188
1872	G	0.131	0.0774
1873	C	0.169	0.0619
1874	A	0.412	0.0820
1875	C	0.136	0.0941
1876	A	0.572	0.1747
1877	G	0.356	0.0938
1878	A	0.268	0.1261
1879	C	0.416	0.0883
1880	A	0.259	0.0679
1881	A	0.417	0.1151
1882	U	0.328	0.1159
1883	U	0.189	0.0699
1884	C	0.409	0.0930
1885	G	0.042	0.0325
1886	A	0.418	0.0869
1887	U	0.280	0.1166
1888	A	0.450	0.1115
1889	C	0.113	0.0214
1890	U	0.424	0.1255
1891	C	0.792	0.4249
1892	A	0.835	0.1335
1893	C	0.335	0.0774
1894	U	0.543	0.0535
1895	U	1.919	0.3835
1896	A	1.853	0.3730
1897	A	2.239	0.3097
1898	A	1.033	0.0967
1899	A	1.823	0.1492

1900	A	0.736	0.0813
1901	U	1.531	0.3851
1902	A	1.278	0.2025
1903	A	0.725	0.1198
1904	C	0.305	0.0281
1905	A	0.791	0.1013
1906	C	0.150	0.0049
1907	C	0.664	0.0975
1908	A	0.601	0.0511
1909	U	0.353	0.0692
1910	C	0.639	0.1069
1911	A	0.653	0.1001
1912	C	0.373	0.1775
1913	G	0.378	0.0171
1914	U	0.260	0.0994
1915	A	0.836	0.1077
1916	U	0.207	0.0042
1917	U	0.500	0.0736
1918	U	0.137	0.0772
1919	U	0.331	0.1164
1920	A	0.757	0.1185
1921	A	0.689	0.0784
1922	C	0.495	0.0884
1923	G	0.359	0.0957
1924	A	0.626	0.0704
1925	A	0.694	0.0908
1926	U	0.327	0.0964
1927	C	0.371	0.0850
1928	A	0.628	0.1612
1929	G	0.918	0.1454
1930	A	0.404	0.0993
1931	U	0.416	0.1046
1932	G	0.438	0.1104
1933	U	0.123	0.0424
1934	C	0.443	0.0910
1935	G	0.595	0.1197
1936	A	0.378	0.1419
1937	C	0.380	0.1070
1938	U	0.489	0.1799
1939	G	0.613	0.0562
1940	G	0.413	0.0639
1941	U	0.193	0.0171
1942	C	0.395	0.1391
1943	U	0.483	0.2060
1944	A	0.445	0.1126
1945	G	0.519	0.1527
1946	U	0.501	0.1690
1947	G	0.367	0.0533
1948	C	0.322	0.1192
1949	U	0.356	0.1302

1950	A	0.827	0.2344
1951	U	0.269	0.0898
1952	U	0.685	0.2500
1953	G	0.554	0.1369
1954	A	0.710	0.1381
1955	C	0.244	0.0613
1956	U	0.457	0.0910
1957	A	1.953	0.3707
1958	U	0.635	0.0894
1959	C	0.484	0.1153
1960	A	1.392	0.3322
1961	A	1.175	0.2124
1962	U	0.655	0.2156
1963	G	0.634	0.1586
1964	U	0.480	0.0947
1965	C	0.414	0.0910
1966	C	0.392	0.0793
1967	U	0.549	0.1778
1968	G	0.253	0.1636
1969	A	0.530	0.2521
1970	U	0.407	0.2582
1971	U	0.862	0.3142
1972	G	0.863	0.1495
1973	U	0.619	0.2552
1974	U	0.967	0.3633
1975	U	0.353	0.0318
1976	A	0.575	0.1438
1977	A	0.757	0.1796
1978	U	0.285	0.0830
1979	C	0.541	0.1344
1980	G	0.735	0.1640
1981	G	0.422	0.1426
1982	C	0.324	0.1473
1983	A	0.848	0.2035
1984	A	0.632	0.1420
1985	A	1.197	0.3238
1986	A	0.330	0.0575
1987	G	0.148	0.0301
1988	C	0.323	0.0582
1989	A	0.573	0.1164
1990	C	0.215	0.0508
1991	C	0.212	0.0166
1992	A	0.839	0.2255
1993	A	0.656	0.1331
1994	A	0.660	0.1206
1995	C	0.363	0.1926
1996	A	0.857	0.1467
1997	C	0.499	0.1281
1998	A	0.715	0.0841
1999	G	0.671	0.0985

2000	A	0.540	0.0546
2001	C	0.605	0.1126
2002	A	0.600	0.0514
2003	U	0.587	0.0815
2004	A	1.022	0.0841
2005	U	0.758	0.0696
2006	C	0.577	0.1495
2007	A	0.717	0.1051
2008	A	0.965	0.2452
2009	A	1.085	0.3174
2010	G	0.521	0.1677
2011	G	0.579	0.1946
2012	U	0.062	0.0297
2013	U	0.135	0.0674
2014	C	0.666	0.2219
2015	A	0.277	0.0232
2016	C	0.599	0.1402
2017	G	0.573	0.1239
2018	A	0.825	0.1995
2019	C	0.501	0.2945
2020	U	0.347	0.1500
2021	A	1.047	0.3292
2022	A	1.110	0.2983
2023	A	0.586	0.0420
2024	A	0.355	0.0933
2025	U	0.555	0.0691
2026	A	0.978	0.1874
2027	C	0.482	0.1033
2028	C	0.445	0.1370
2029	A	1.058	0.2366
2030	A	1.368	0.2485
2031	A	0.950	0.2019
2032	A	1.143	0.2712
2033	U	0.530	0.1200
2034	U	0.888	0.1582
2035	C	0.882	0.2537
2036	A	0.569	0.0772
2037	U	1.011	0.1662
2038	A	1.063	0.1953
2039	C	0.208	0.0941
2040	G	0.376	0.1748
2041	A	0.267	0.1110
2042	A	0.537	0.2061
2043	C	0.226	0.0536
2044	C	0.314	0.1168
2045	C	0.273	0.1231
2046	U	0.641	0.1368
2047	U	0.582	0.0856
2048	U	0.673	0.1282
2049	C	0.990	0.2070

2050	A	0.981	0.2005
2051	A	0.301	0.1129
2052	U	0.689	0.1373
2053	A	0.987	0.2366
2054	C	0.320	0.0368
2055	C	0.289	0.0523
2056	U	0.615	0.0288
2057	A	0.823	0.0985
2058	C	0.468	0.0578
2059	A	0.626	0.0622
2060	U	0.853	0.1071
2061	A	0.638	0.0833
2062	C	0.240	0.0348
2063	U	0.413	0.1012
2064	G	0.593	0.0885
2065	A	0.405	0.0542
2066	C	0.276	0.0370
2067	A	0.512	0.0813
2068	U	0.445	0.0485
2069	A	0.461	0.1051
2070	U	0.292	0.0493
2071	U	0.327	0.0248
2072	U	0.297	0.0428
2073	G	0.285	0.0644
2074	G	0.272	0.0422
2075	U	0.151	0.0297
2076	C	0.291	0.0302
2077	C	0.321	0.0336
2078	A	0.489	0.0466
2079	G	0.325	0.0561
2080	U	0.302	0.0358
2081	U	0.421	0.0304
2082	C	0.816	0.0419
2083	A	0.374	0.0965
2084	C	0.305	0.1063
2085	A	0.438	0.0928
2086	A	0.640	0.0583
2087	C	0.068	0.0396
2088	C	0.119	0.0757
2089	U	0.394	0.1052
2090	A	0.544	0.1456
2091	C	0.074	0.0035
2092	C	0.238	0.0491
2093	A	0.312	0.0032
2094	A	0.198	0.0237
2095	A	0.319	0.0011
2096	U	0.241	0.0509
2097	A	0.162	0.0428
2098	G	0.505	0.0329
2099	U	0.359	0.0573

2100	G	0.421	0.1623
2101	C	0.177	0.0286
2102	A	0.233	0.1082
2103	C	0.250	0.1390
2104	C	0.312	0.1215
2105	A	0.480	0.0937
2106	U	0.217	0.0416
2107	C	0.247	0.1486
2108	C	0.219	0.0829
2109	U	0.414	0.1931
2110	A	0.424	0.1225
2111	U	0.366	0.0516
2112	U	0.521	0.2489
2113	U	0.293	0.1461
2114	C	0.356	0.2631
2115	A	0.483	0.3983
2116	U	0.172	0.0477
2117	C	0.145	0.0314
2118	U	0.128	0.0479
2119	C	0.294	0.0796
2120	A	0.768	0.1276
2121	U	0.155	0.1376
2122	U	0.264	0.0542
2123	U	0.354	0.1428
2124	A	0.751	0.0829
2125	C	0.131	0.0298
2126	U	0.382	0.0205
2127	G	0.385	0.0262
2128	A	0.110	0.1134
2129	U	0.059	0.0191
2130	G	0.096	0.0487
2131	A	0.162	0.1478
2132	G	0.301	0.1562
2133	A	0.554	0.1771
2134	C	0.189	0.0941
2135	A	0.337	0.2898
2136	A	0.750	0.2637
2137	C	0.650	0.2786
2138	A	0.369	0.1377
2139	A	0.735	0.2451
2140	A	0.958	0.3361
2141	A	0.741	0.2824
2142	U	0.281	0.2366
2143	U	0.129	0.0439
2144	C	0.422	0.0981
2145	C	0.388	0.0598
2146	G	0.188	0.0481
2147	U	0.262	0.1044
2148	U	-	0.0000
2149	G	0.311	0.1683

2150	G	0.381	0.2892
2151	G	0.262	0.0502
2152	U	0.268	0.1336
2153	U	0.262	0.0787
2154	U	0.161	0.1398
2155	A	0.279	0.2043
2156	U	0.386	0.2016
2157	C	0.220	0.2086
2158	C	-	0.0000
2159	A	0.454	0.0028
2160	U	0.178	0.0177
2161	U	0.346	0.0354
2162	A	0.560	0.1234
2163	C	0.129	0.0849
2164	A	0.695	0.0822
2165	C	0.151	0.0860
2166	G	0.122	0.0729
2167	A	-	0.0000
2168	C	-	0.0000
2169	C	0.179	0.0960
2170	G	0.409	0.0296
2171	U	0.227	0.1083
2172	C	0.227	0.0332
2173	G	0.857	0.1616
2174	C	0.289	0.0683
2175	G	0.336	0.3674
2176	A	0.573	0.3046
2177	G	0.215	0.0912
2178	G	0.143	0.0970
2179	A	-	0.0000
2180	C	0.457	0.0675
2181	U	0.491	0.0759
2182	C	0.504	0.0247
2183	U	0.138	0.0926
2184	A	0.342	0.0983
2185	U	0.739	0.1796
2186	C	-	0.0000
2187	C	0.066	0.0170
2188	U	0.324	0.0718
2189	C	0.122	0.0455
2190	G	0.126	0.0339
2191	A	0.355	0.3076
2192	U	0.571	0.1137
2193	G	0.355	0.0709
2194	U	0.494	0.1223
2195	U	0.300	0.1891
2196	U	0.667	0.1176
2197	U	0.087	0.1117
2198	U	0.327	0.2181
2199	A	0.934	0.0384

2200	C	0.164	0.1178
2201	U	0.137	0.0252
2202	A	0.415	0.1040
2203	C	0.550	0.1294
2204	G	0.311	0.1370
2205	A	0.150	0.0205
2206	U	0.717	0.0972
2207	A	0.329	0.0337
2208	C	0.545	0.1078
2209	U	0.078	0.0622
2210	A	0.431	0.0966
2211	G	0.618	0.1235
2212	C	0.559	0.0362
2213	U	0.253	0.0081
2214	U	0.402	0.0493
2215	U	0.324	0.0720
2216	U	0.234	0.0951
2217	A	0.322	0.0998
2218	U	1.236	0.0731
2219	U	0.581	0.1700
2220	A	1.465	0.4295
2221	A	1.020	0.0392
2222	A	0.181	0.0650
2223	A	0.441	0.1601
2224	A	0.638	0.2141
2225	C	0.335	0.0612
2226	C	0.380	0.0476
2227	A	0.297	0.0422
2228	G	0.942	0.0642
2229	U	0.637	0.1373
2230	U	0.524	0.0315
2231	U	0.484	0.1026
2232	C	0.401	0.0340
2233	A	0.596	0.1829
2234	G	0.495	0.0215
2235	G	0.538	0.1897
2236	C	-	0.0000
2237	C	0.161	0.0532
2238	A	0.203	0.0523
2239	G	0.916	0.1457
2240	U	0.200	0.0300
2241	G	0.492	0.0281
2242	U	0.543	0.0838
2243	C	0.311	0.0461
2244	U	0.225	0.0131
2245	U	0.564	0.0140
2246	G	0.542	0.0787
2247	G	0.432	0.0170
2248	U	0.309	0.0396
2249	U	0.293	0.0823

2250	A	0.612	0.1262
2251	U	0.478	0.0722
2252	A	-	0.0000
2253	C	0.569	0.0717
2254	A	0.523	0.1505
2255	A	0.599	0.1205
2256	A	0.382	0.1113
2257	U	0.795	0.1184
2258	G	0.844	0.1973
2259	G	-	0.0000
2260	A	0.253	0.1725
2261	C	0.392	0.1861
2262	C	0.297	0.1300
2263	G	0.416	0.1133
2264	U	0.425	0.2881
2265	G	0.743	0.2600
2266	G	0.379	0.1120
2267	U	0.368	0.1692
2268	U	0.494	0.1340
2269	C	0.350	0.2668
2270	U	0.506	0.3891
2271	G	0.615	0.1263
2272	A	0.952	0.0819
2273	G	0.761	0.1120
2274	U	0.417	0.0595
2275	A	2.229	0.3072
2276	U	0.761	0.0789
2277	A	1.950	0.1773
2278	C	0.389	0.1506
2279	U	0.284	0.0174
2280	A	0.970	0.0718
2281	A	0.613	0.1352
2282	C	0.527	0.1556
2283	A	0.484	0.0812
2284	G	0.657	0.0743
2285	A	0.159	0.0804
2286	A	0.499	0.1693
2287	C	0.081	0.0565
2288	U	0.099	0.0280
2289	C	0.260	0.1004
2290	U	0.383	0.0415
2291	C	0.293	0.0633
2292	C	0.371	0.0216
2293	A	0.584	0.0101
2294	U	0.652	0.0336
2295	A	1.522	0.0266
2296	A	1.067	0.0814
2297	A	0.866	0.0601
2298	U	0.605	0.1202
2299	U	0.325	0.0999

2300	C	0.349	0.0824
2301	C	0.564	0.0761
2302	U	0.554	0.0395
2303	U	1.454	0.1683
2304	G	0.797	0.1546
2305	A	0.894	0.1328
2306	A	1.379	0.1013
2307	A	1.571	0.1210
2308	A	2.011	0.1824
2309	A	0.975	0.0963
2310	A	0.691	0.1132
2311	A	0.756	0.0618
2312	U	0.558	0.0938
2313	G	0.433	0.0739
2314	G	0.304	0.0156
2315	U	0.540	0.0286
2316	A	0.438	0.1079
2317	U	0.600	0.0384
2318	A	0.837	0.0570
2319	A	0.675	0.0229
2320	C	0.429	0.0734
2321	U	0.382	0.0276
2322	C	0.161	0.0370
2323	C	0.135	0.0573
2324	A	0.710	0.0074
2325	U	0.736	0.1101
2326	G	0.433	0.0134
2327	C	0.171	0.1007
2328	U	0.465	0.2300
2329	A	0.719	0.0274
2330	U	1.052	0.0518
2331	A	0.633	0.0501
2332	C	0.494	0.1361
2333	A	1.087	0.0326
2334	A	0.606	0.0323
2335	C	0.256	0.0464
2336	C	1.012	0.0991
2337	A	0.196	0.0476
2338	C	0.440	0.1164
2339	A	0.836	0.0367
2340	G	0.709	0.0751
2341	C	0.713	0.1240
2342	G	0.699	0.0609
2343	G	0.676	0.0363
2344	A	0.547	0.0669
2345	U	0.397	0.0279
2346	U	0.299	0.0600
2347	C	0.234	0.0722
2348	C	0.205	0.1018
2349	C	0.368	0.0964

2350	G	0.788	0.1788
2351	A	1.029	0.3263
2352	G	0.516	0.0538
2353	C	0.804	0.0784
2354	A	0.789	0.2304
2355	C	0.410	0.0536
2356	A	0.975	0.0815
2357	U	0.475	0.0418
2358	G	0.506	0.1008
2359	G	0.115	0.0463
2360	A	0.518	0.0583
2361	G	0.253	0.0579
2362	U	0.360	0.0357
2363	C	0.520	0.0094
2364	G	0.288	0.0056
2365	C	0.462	0.0275
2366	U	0.411	0.0171
2367	G	0.631	0.0693
2368	A	0.784	0.0205
2369	A	0.150	0.0095
2370	C	0.312	0.0745
2371	G	-	0.0000
2372	G	-	0.0000
2373	C	0.445	0.1311
2374	U	0.815	0.0549
2375	A	0.778	0.0472
2376	A	0.763	0.0188
2377	A	0.802	0.0737
2378	C	0.205	0.0438
2379	C	0.434	0.0645
2380	G	0.242	0.0253
2381	U	0.289	0.0025
2382	A	0.989	0.0231
2383	C	0.280	0.0270
2384	C	0.490	0.0050
2385	U	0.794	0.0294
2386	U	0.740	0.0293
2387	A	1.365	0.0466
2388	U	1.157	0.0950
2389	U	0.862	0.0128
2390	A	1.092	0.0774
2391	G	1.189	0.0566
2392	A	0.493	0.0816
2393	U	0.524	0.0965
2394	G	0.679	0.1033
2395	A	0.736	0.0510
2396	C	0.088	0.0053
2397	U	0.492	0.0551
2398	G	0.493	0.1393
2399	C	0.328	0.0797

2400	C	0.253	0.0187
2401	G	0.307	0.1107
2402	U	0.299	0.0761
2403	A	0.816	0.0962
2404	C	0.547	0.0838
2405	U	0.145	0.0103
2406	C	0.623	0.1475
2407	A	0.875	0.1412
2408	A	0.554	0.0954
2409	C	0.226	0.0648
2410	U	0.831	0.0926
2411	G	0.280	0.1375
2412	C	-	0.0000
2413	A	0.768	0.2124
2414	A	0.989	0.2010
2415	U	0.497	0.1758
2416	G	0.427	0.0702
2417	U	1.611	0.2585
2418	A	0.555	0.1513
2419	G	0.414	0.0953
2420	U	0.699	0.0597
2421	G	0.477	0.0890
2422	G	0.446	0.0387
2423	U	0.111	0.0126
2424	U	0.315	0.0473
2425	U	0.574	0.0125
2426	A	0.971	0.0869
2427	C	0.381	0.0826
2428	C	0.558	0.1767
2429	G	0.382	0.1208
2430	A	0.531	0.0696
2431	A	0.513	0.1523
2432	C	0.228	0.0436
2433	C	0.532	0.2848
2434	A	0.933	0.1110
2435	U	0.693	0.1404
2436	U	0.634	0.0938
2437	U	0.654	0.2094
2438	A	0.969	0.0719
2439	U	0.690	0.1648
2440	G	0.373	0.1241
2441	G	0.311	0.0566
2442	U	0.245	0.1037
2443	U	0.299	0.1054
2444	C	0.322	0.0711
2445	U	0.209	0.1189
2446	C	0.389	0.0793
2447	U	0.747	0.1490
2448	G	0.583	0.1715
2449	C	0.173	0.0574

2450	A	0.721	0.1956
2451	A	0.762	0.1614
2452	U	0.565	0.1385
2453	C	0.380	0.1453
2454	G	0.491	0.1377
2455	A	0.674	0.0808
2456	A	0.695	0.0862
2457	U	0.307	0.0466
2458	U	0.276	0.0532
2459	U	0.592	0.0952
2460	U	0.145	0.1050
2461	C	0.305	0.1043
2462	U	0.534	0.0881
2463	A	0.602	0.0819
2464	C	0.167	0.0714
2465	U	0.285	0.0729
2466	A	1.192	0.2143
2467	U	0.491	0.1153
2468	U	0.436	0.1519
2469	G	0.168	0.0416
2470	U	0.414	0.1056
2471	G	0.265	0.0384
2472	A	0.401	0.0930
2473	G	0.310	0.1227
2474	A	0.909	0.1373
2475	A	0.614	0.3156
2476	A	0.433	0.1729
2477	U	0.119	0.1163
2478	U	0.302	0.1457
2479	C	0.263	0.1636
2480	A	1.003	0.5001
2481	C	1.541	0.7107
2482	U	0.373	0.1998
2483	A	0.631	0.1088
2484	G	0.036	0.0234
2485	C	0.178	0.0329
2486	U	0.191	0.0591
2487	U	0.084	0.0276
2488	C	0.335	0.0892
2489	A	0.689	0.0961
2490	C	0.202	0.0292
2491	C	0.561	0.1296
2492	U	0.318	0.0935
2493	A	0.390	0.1166
2494	A	0.497	0.0640
2495	A	0.445	0.1109
2496	A	0.463	0.1158
2497	G	0.484	0.0939
2498	C	0.113	0.0442
2499	A	0.518	0.1218

2500	A	0.509	0.0379
2501	A	0.822	0.0765
2502	A	0.413	0.0913
2503	A	0.600	0.0626
2504	A	0.696	0.1156
2505	U	0.289	0.1215
2506	C	0.290	0.0777
2507	U	0.643	0.4342
2508	G	0.116	0.6628
2509	C	0.544	0.1044
2510	A	0.249	0.0270
2511	A	0.419	0.1201
2512	G	0.514	0.0662
2513	A	0.172	0.0828
2514	C	0.299	0.0583
2515	A	0.493	0.0739
2516	A	0.270	0.1467
2517	C	0.158	0.0601
2518	A	0.753	0.0947
2519	U	0.381	0.0560
2520	G	0.310	0.1761
2521	C	0.208	0.0877
2522	U	0.435	0.0913
2523	G	0.351	0.0852
2524	G	0.086	0.1153
2525	C	0.112	0.0566
2526	U	0.221	0.0391
2527	U	0.266	0.0276
2528	G	0.304	0.0766
2529	G	0.042	0.0170
2530	C	0.303	0.0987
2531	A	0.368	0.0986
2532	G	0.222	0.0815
2533	G	-	0.0000
2534	A	0.139	0.0269
2535	C	0.185	0.0501
2536	U	0.350	0.0490
2537	U	0.421	0.0482
2538	G	0.774	0.2383
2539	A	0.543	0.1273
2540	U	0.317	0.0676
2541	A	0.306	0.1049
2542	U	0.343	0.1038
2543	C	0.497	0.1428
2544	A	0.257	0.1350
2545	G	0.108	0.0402
2546	U	0.101	0.0548
2547	A	0.312	0.2628
2548	C	0.410	0.4791
2549	U	0.557	0.3080

2550	U	0.372	0.0721
2551	U	0.250	0.0739
2552	G	0.395	0.0771
2553	U	0.240	0.0882
2554	U	0.238	0.0431
2555	A	1.044	0.3040
2556	C	0.894	0.2341
2557	C	0.097	0.0445
2558	U	0.363	0.0978
2559	U	0.368	0.0989
2560	U	0.499	0.1378
2561	C	0.460	0.0798
2562	G	0.425	0.1238
2563	G	0.339	0.1347
2564	U	0.170	0.1208
2565	C	0.282	0.0630
2566	A	0.486	0.1227
2567	A	0.378	0.1732
2568	C	0.234	0.1140
2569	C	0.391	0.1216
2570	U	0.339	0.1984
2571	G	0.448	0.2131
2572	U	-	0.0000
2573	U	0.259	0.1792
2574	A	0.611	0.0799
2575	U	0.228	0.0808
2576	C	0.419	0.1075
2577	G	0.368	0.1207
2578	U	0.203	0.0455
2579	C	0.287	0.0754
2580	A	0.575	0.1516
2581	A	0.721	0.1416
2582	U	0.427	0.1360
2583	G	0.519	0.1604
2584	A	0.522	0.1227
2585	U	0.266	0.0715
2586	C	0.901	0.1436
2587	A	0.175	0.0920
2588	C	0.435	0.0968
2589	A	0.199	0.0253
2590	A	0.522	0.0765
2591	C	0.153	0.0627
2592	C	0.205	0.0255
2593	C	0.218	0.0526
2594	U	0.861	0.0804
2595	A	0.790	0.0773
2596	A	0.632	0.1129
2597	C	0.388	0.1749
2598	U	0.688	0.2274
2599	C	0.458	0.1114

2600	C	0.592	0.0962
2601	A	1.296	0.1667
2602	A	0.979	0.2182
2603	A	0.913	0.1485
2604	A	1.257	0.1436
2605	U	1.536	0.2742
2606	A	0.746	0.1138
2607	C	0.619	0.1685
2608	A	0.902	0.1277
2609	U	0.424	0.0597
2610	C	0.434	0.0535
2611	C	0.570	0.1042
2612	U	0.170	0.0896
2613	C	0.794	0.1941
2614	G	-	0.0000
2615	U	0.800	0.1424
2616	G	0.594	0.1471
2617	G	0.120	0.0444
2618	C	0.361	0.0520
2619	A	0.526	0.0246
2620	U	0.481	0.0743
2621	C	0.212	0.1640
2622	C	0.259	0.0269
2623	C	0.233	0.1979
2624	A	0.147	0.0499
2625	G	0.677	0.1187
2626	G	1.070	0.3924
2627	C	-	0.0000
2628	U	-	0.0000
2629	A	0.488	0.9184
2630	C	0.492	0.1458
2631	G	-	0.0000
2632	C	0.574	0.1399
2633	U	0.256	0.1622
2634	C	0.400	0.1080
2635	U	1.024	0.0721
2636	A	0.517	0.0925
2637	C	0.733	0.1857
2638	A	0.476	0.0984
2639	U	0.576	0.1190
2640	C	0.313	0.0669
2641	C	0.586	0.0643
2642	G	0.423	0.0411
2643	U	0.273	0.0499
2644	C	0.533	0.0662
2645	U	0.360	0.0561
2646	C	0.580	0.0137
2647	G	0.320	0.0312
2648	A	0.984	0.0410
2649	A	0.809	0.0177

2650	A	0.785	0.0300
2651	C	0.275	0.0286
2652	U	0.562	0.0342
2653	C	0.642	0.0464
2654	U	1.095	0.1528
2655	U	0.755	0.0703
2656	A	0.921	0.0718
2657	U	0.686	0.0878
2658	G	0.396	0.0666
2659	G	0.361	0.0384
2660	A	0.602	0.0641
2661	U	0.508	0.0573
2662	A	0.505	0.0488
2663	U	1.214	0.1253
2664	A	0.971	0.0525
2665	U	0.248	0.0335
2666	C	0.442	0.0566
2667	A	0.896	0.0352
2668	U	0.430	0.0687
2669	C	0.219	0.0655
2670	U	0.437	0.0517
2671	A	0.938	0.0495
2672	U	0.291	0.0558
2673	C	0.467	0.0388
2674	U	0.471	0.0293
2675	U	0.521	0.0492
2676	C	0.072	0.0049
2677	C	0.172	0.0240
2678	A	1.122	0.0997
2679	U	0.467	0.1028
2680	C	0.545	0.1602
2681	C	0.706	0.1704
2682	U	0.503	0.0932
2683	U	0.414	0.0343
2684	A	1.010	0.0399
2685	A	1.155	0.0615
2686	A	0.442	0.0448
2687	G	0.833	0.0049
2688	A	0.637	0.0411
2689	A	0.468	0.0553
2690	G	0.227	0.0141
2691	A	0.680	0.0160
2692	C	0.655	0.0331
2693	A	0.418	0.0306
2694	G	0.411	0.0330
2695	U	0.390	0.0416
2696	A	0.878	0.0520
2697	G	0.489	0.0170
2698	A	0.601	0.0083
2699	U	0.870	0.0425

2700	A	0.483	0.0388
2701	C	0.581	0.0872
2702	A	1.144	0.0733
2703	A	1.109	0.0872
2704	C	0.174	0.0672
2705	U	0.683	0.0619
2706	A	1.171	0.1223
2707	A	0.909	0.0547
2708	C	0.286	0.0333
2709	U	0.805	0.0543
2710	A	0.913	0.0537
2711	U	0.540	0.0550
2712	G	0.438	0.0717
2713	U	0.541	0.0682
2714	U	0.575	0.0968
2715	A	1.186	0.1459
2716	U	0.552	0.1658
2717	U	0.405	0.2186
2718	C	0.611	0.1955
2719	U	0.614	0.0071
2720	U	0.321	0.0544
2721	C	0.523	0.0630
2722	A	0.980	0.0544
2723	G	0.516	0.0655
2724	G	0.468	0.0951
2725	G	0.185	0.1040
2726	C	0.379	0.0560
2727	A	0.832	0.1088
2728	A	1.058	0.1849
2729	G	0.313	0.0905
2730	G	0.376	0.0373
2731	A	0.435	0.0266
2732	A	0.686	0.1033
2733	U	0.207	0.0355
2734	C	0.213	0.0631
2735	C	0.394	0.1573
2736	A	1.012	0.2104
2737	G	0.834	0.1404
2738	A	0.602	0.1241
2739	U	0.627	0.1279
2740	U	0.797	0.1157
2741	A	0.815	0.1028
2742	G	0.561	0.0866
2743	A	0.792	0.0944
2744	U	0.509	0.0477
2745	C	0.636	0.0916
2746	A	0.861	0.0795
2747	A	1.098	0.7865
2748	U	0.723	0.2897
2749	U	0.480	0.1535

2750	C	0.644	0.1472
2751	A	1.042	0.3557
2752	A	0.520	0.1995
2753	U	0.312	0.0767
2754	U	1.419	0.5961
2755	A	0.928	0.4360
2756	C	1.056	0.6464
2757	G	0.288	0.0911
2758	A	0.544	0.3919
2759	C	0.401	0.3304
2760	G	0.231	0.0385
2761	C	0.268	0.2541
2762	A	0.410	0.0927
2763	C	0.262	0.1252
2764	U	0.369	0.1573
2765	C	0.569	0.2518
2766	A	0.528	0.1281
2767	C	0.169	0.0178
2768	U	0.233	0.0728
2769	U	0.455	0.1010
2770	U	0.379	0.1203
2771	C	0.323	0.0848
2772	G	0.264	0.1545
2773	A	0.378	0.1384
2774	U	0.201	0.0909
2775	G	0.332	0.0984
2776	A	0.080	0.0180
2777	A	0.376	0.1028
2778	G	0.137	0.0430
2779	A	0.430	0.1337
2780	C	0.132	0.0522
2781	U	0.550	0.2052
2782	U	0.791	0.2044
2783	A	0.599	0.1784
2784	A	0.755	0.3340
2785	A	1.149	0.4596
2786	C	0.362	0.1656
2787	C	0.800	0.3332
2788	G	0.670	0.2397
2789	U	0.344	0.1800
2790	U	0.291	0.1428
2791	U	0.332	0.1616
2792	A	0.812	0.2317
2793	A	0.448	0.1267
2794	C	0.123	0.0578
2795	U	0.529	0.1493
2796	G	0.198	0.0514
2797	C	0.168	0.0810
2798	U	0.257	0.1191
2799	U	0.334	0.3425

2800	C	0.322	0.2025
2801	A	0.320	0.0635
2802	U	0.163	0.0600
2803	A	0.552	0.2606
2804	U	0.406	0.3279
2805	C	0.319	0.0538
2806	A	0.320	0.0677
2807	U	0.261	0.0403
2808	U	0.451	0.1652
2809	C	0.395	0.0936
2810	G	0.331	0.1671
2811	U	0.304	0.1036
2812	U	0.244	0.0721
2813	C	0.398	0.0719
2814	A	0.298	0.0547
2815	U	0.172	0.1026
2816	U	0.300	0.0507
2817	G	0.084	0.0551
2818	C	0.181	0.1013
2819	G	0.358	0.1621
2820	U	0.177	0.0343
2821	C	0.289	0.0501
2822	A	0.370	0.0702
2823	A	0.577	0.1421
2824	A	0.425	0.1071
2825	U	0.821	0.4060
2826	G	0.594	0.1685
2827	A	0.173	0.0478
2828	G	0.193	0.0531
2829	A	0.399	0.1053
2830	U	0.136	0.0446
2831	C	0.064	0.0085
2832	C	0.309	0.0760
2833	A	0.508	0.1063
2834	A	0.859	0.2070
2835	G	0.462	0.1153
2836	A	0.791	0.1036
2837	A	0.839	0.2691
2838	U	0.468	0.3037
2839	C	0.271	0.0797
2840	C	0.274	0.0909
2841	A	0.473	0.1059
2842	A	0.539	0.1096
2843	U	0.293	0.1754
2844	G	1.035	0.7142
2845	A	0.438	0.0512
2846	U	0.285	0.0428
2847	C	1.167	1.2652
2848	U	0.480	0.3877
2849	U	0.479	0.9624

2850	A	0.618	0.3448
2851	A	0.693	0.0391
2852	C	0.403	0.0838
2853	A	0.707	0.0657
2854	U	0.801	0.1826
2855	A	0.986	0.0691
2856	G	0.507	0.0850
2857	A	0.581	0.0894
2858	A	0.426	0.0376
2859	U	0.348	0.0737
2860	C	0.156	0.0595
2861	U	0.383	0.1346
2862	G	0.220	0.0127
2863	A	0.454	0.0775
2864	C	0.036	0.0113
2865	C	0.566	0.2316
2866	A	0.432	0.1616
2867	U	0.378	0.0809
2868	G	0.549	0.0889
2869	A	0.268	0.0980
2870	C	0.238	0.0555
2871	U	0.351	0.0351
2872	U	0.365	0.0084
2873	C	0.299	0.0571
2874	C	0.280	0.0323
2875	A	0.455	0.1357
2876	A	0.412	0.0380
2877	U	0.420	0.0705
2878	C	0.274	0.1730
2879	C	0.363	0.0439
2880	G	0.597	0.1725
2881	A	0.525	0.1265
2882	C	0.280	0.1677
2883	A	0.776	0.0860
2884	U	0.671	0.0873
2885	U	0.228	0.1227
2886	G	0.344	0.0792
2887	A	0.791	0.0774
2888	A	0.442	0.1217
2889	C	0.361	0.0670
2890	U	0.897	0.0248
2891	A	0.224	0.1732
2892	C	0.456	0.0577
2893	A	0.577	0.1820
2894	U	0.289	0.1402
2895	C	0.344	0.0440
2896	C	0.305	0.0931
2897	U	0.669	0.2277
2898	G	0.729	0.2573
2899	A	0.902	0.5166

2900	G	0.201	0.0892
2901	C	0.309	0.1556
2902	A	0.684	0.1170
2903	A	0.867	0.0907
2904	C	0.175	0.1302
2905	C	0.361	0.1447
2906	G	0.572	0.2276
2907	A	0.849	0.2050
2908	G	0.682	0.0434
2909	A	0.605	0.0924
2910	A	0.883	0.1240
2911	A	0.520	0.1453
2912	U	0.235	0.0908
2913	G	0.415	0.1817
2914	U	0.247	0.1383
2915	C	0.305	0.0737
2916	C	0.370	0.0233
2917	U	0.416	0.0946
2918	U	0.671	0.1572
2919	U	0.607	0.0806
2920	C	0.351	0.2091
2921	A	0.764	0.0785
2922	A	0.876	0.2786
2923	A	0.890	0.1106
2924	A	0.710	0.5184
2925	G	0.417	0.0753
2926	C	0.247	0.1391
2927	U	-	0.0000
2928	G	0.313	0.2217
2929	U	0.498	0.0464
2930	G	0.525	0.2071
2931	A	0.544	0.0731
2932	G	0.381	0.0346
2933	U	0.388	0.0829
2934	C	0.144	0.0722
2935	C	0.429	0.0651
2936	A	0.685	0.1015
2937	A	0.736	0.1473
2938	C	0.405	0.0968
2939	C	0.595	0.0339
2940	G	0.651	0.0877
2941	A	0.298	0.0552
2942	U	0.511	0.1676
2943	U	0.355	0.0512
2944	C	0.350	0.0562
2945	C	0.645	0.0922
2946	A	0.267	0.0600
2947	C	0.470	0.1069
2948	A	0.471	0.0884
2949	C	0.086	0.0670

2950	C	0.156	0.0420
2951	U	0.722	0.1714
2952	C	0.411	0.0609
2953	C	0.497	0.0789
2954	G	0.450	0.1191
2955	U	0.168	0.0104
2956	C	0.580	0.0335
2957	A	0.298	0.0258
2958	A	0.487	0.1285
2959	C	1.261	0.4596
2960	U	0.494	0.2814
2961	C	0.581	0.1202
2962	A	0.573	0.0800
2963	U	0.353	0.0827
2964	A	0.753	0.0735
2965	C	0.367	0.1276
2966	U	0.389	0.1000
2967	G	0.458	0.0205
2968	A	0.507	0.1062
2969	A	0.870	0.1656
2970	G	0.501	0.0471
2971	A	0.471	0.1190
2972	U	0.210	0.2065
2973	U	1.439	1.9725
2974	C	0.701	0.1631
2975	G	0.425	0.0616
2976	A	0.390	0.2009
2977	A	0.789	0.1478
2978	A	0.555	0.1169
2979	C	0.519	0.1295
2980	G	0.391	0.0450
2981	U	0.555	0.1402
2982	G	0.279	0.0133
2983	U	0.334	0.0748
2984	U	0.529	0.0623
2985	U	0.518	0.1141
2986	C	0.585	0.0691
2987	U	0.978	0.2557
2988	A	0.710	0.1400
2989	A	0.997	0.2753
2990	A	0.712	0.1745
2991	A	0.788	0.1314
2992	C	0.338	0.1046
2993	C	0.493	0.1537
2994	A	1.007	0.1108
2995	A	1.125	0.3618
2996	U	0.642	0.0531
2997	A	1.058	0.2787
2998	U	0.451	0.1678
2999	U	0.406	0.1412

3000	C	0.507	0.1732
3001	G	0.411	0.0966
3002	C	1.619	0.2889
3003	G	0.520	0.0935
3004	C	0.452	0.0619
3005	A	0.548	0.1086
3006	C	0.251	0.0403
3007	C	0.193	0.1074
3008	C	0.413	0.1076
3009	A	0.954	0.1363
3010	G	0.682	0.0805
3011	A	0.574	0.1105
3012	G	0.620	0.1347
3013	A	0.740	0.1688
3014	A	0.892	0.1134
3015	G	0.554	0.1105
3016	U	0.327	0.0764
3017	U	0.332	0.0855
3018	G	0.452	0.0670
3019	A	0.413	0.2051
3020	C	0.251	0.0520
3021	C	0.226	0.0876
3022	C	0.141	0.1450
3023	C	0.297	0.1523
3024	A	1.144	0.2172
3025	A	0.484	0.3221
3026	C	0.692	0.3615
3027	A	0.768	0.0264
3028	U	0.613	0.3375
3029	A	0.829	0.2236
3030	U	0.616	0.3701
3031	C	0.276	0.1926
3032	U	0.811	0.4348
3033	G	0.580	0.2174
3034	A	0.792	0.4525
3035	A	0.947	0.3881
3036	U	0.556	0.0343
3037	C	0.421	0.2405
3038	U	1.329	0.6126
3039	A	1.002	0.3949
3040	A	1.133	0.4612
3041	U	0.583	0.1667
3042	A	0.951	0.5138
3043	U	0.537	0.3120
3044	U	0.251	0.0562
3045	C	0.229	0.0870
3046	U	0.340	0.0811
3047	U	0.550	0.1118
3048	C	0.132	0.0267
3049	C	0.411	0.0622

3050	A	1.330	0.1786
3051	U	0.841	0.0891
3052	C	0.702	0.0576
3053	A	1.030	0.0971
3054	A	1.077	0.2004
3055	A	2.488	0.1509
3056	G	-	0.0000
3057	A	0.845	0.0241
3058	A	1.072	0.1398
3059	G	0.702	0.1353
3060	A	0.475	0.0771
3061	G	0.648	0.0596
3062	A	0.613	0.1374
3063	U	0.310	0.0474
3064	C	1.443	0.1867
3065	U	-	0.0000
3066	A	0.804	0.1867
3067	G	0.162	0.0455
3068	C	0.421	0.0579
3069	A	0.343	0.0381
3070	C	0.192	0.0416
3071	C	0.349	0.1352
3072	C	0.463	0.0641
3073	C	0.311	0.0592
3074	C	0.475	0.0181
3075	C	0.509	0.1468
3076	A	0.602	0.1812
3077	A	0.855	0.2054
3078	A	0.595	0.1852
3079	U	0.292	0.1871
3080	U	0.445	0.2067
3081	U	0.469	0.1401
3082	C	0.232	0.1148
3083	C	0.276	0.1144
3084	A	0.837	0.2035
3085	A	0.541	0.1381
3086	U	0.523	0.2010
3087	A	0.884	0.1763
3088	U	0.592	0.1522
3089	C	0.664	0.1037
3090	G	0.465	0.1484
3091	A	0.744	0.2014
3092	G	0.690	0.0896
3093	A	0.269	0.0637
3094	G	0.090	0.0314
3095	U	0.363	0.1201
3096	A	0.374	0.1577
3097	C	0.116	0.0315
3098	C	0.574	0.0799
3099	G	0.268	0.0587

3100	G	0.244	0.0717
3101	U	0.302	0.0398
3102	U	0.399	0.2057
3103	C	0.379	0.1182
3104	G	0.344	0.1302
3105	G	0.221	0.0955
3106	G	0.257	0.0460
3107	U	0.265	0.0223
3108	G	0.107	0.0255
3109	G	0.041	0.0209
3110	U	0.184	0.0803
3111	A	0.272	0.0315
3112	U	0.286	0.0976
3113	G	0.244	0.1389
3114	C	0.170	0.0301
3115	A	0.361	0.0463
3116	U	0.453	0.0000
3117	A	0.490	0.1450
3118	A	0.496	0.1153
3119	A	0.439	0.0626
3120	U	0.336	0.1195
3121	U	0.463	0.1513
3122	A	0.732	0.1821
3123	A	0.584	0.0000
3124	A	0.596	0.0293
3125	U	0.441	0.0071
3126	G	0.251	0.0481
3127	U	0.153	0.0095
3128	U	0.258	0.0410
3129	C	0.189	0.0247
3130	C	0.316	0.0244
3131	U	0.192	0.0923
3132	U	0.401	0.0216
3133	U	0.461	0.0276
3134	A	0.595	0.0095
3135	C	0.189	0.0612
3136	U	0.444	0.0297
3137	U	0.510	0.0516
3138	G	0.166	0.0156
3139	C	0.200	0.0293
3140	U	0.194	0.0997
3141	C	0.165	0.1103
3142	C	0.061	0.0106
3143	C	0.306	0.2029
3144	A	0.358	0.1167
3145	U	0.389	0.0651
3146	G	0.241	0.0403
3147	U	0.238	0.1393
3148	C	0.074	0.0481
3149	C	-	0.0000

3150	C	0.195	0.0127
3151	A	0.688	0.2022
3152	A	0.471	0.1471
3153	U	0.304	0.1117
3154	C	0.128	0.0318
3155	U	0.631	0.1096
3156	A	0.478	0.0877
3157	A	0.698	0.0707
3158	C	0.266	0.0622
3159	A	0.565	0.0212
3160	C	0.461	0.0587
3161	A	0.566	0.0742
3162	C	0.523	0.0247
3163	A	0.311	0.0410
3164	U	0.670	0.0000
3165	G	0.333	0.1032
3166	A	0.154	0.0325
3167	G	0.348	0.1160
3168	U	0.058	0.0000
3169	C	0.278	0.0191
3170	G	0.353	0.1534
3171	U	0.512	0.0255
3172	C	0.215	0.1146
3173	G	0.078	0.0573
3174	C	0.354	0.0106
3175	A	0.569	0.1485
3176	C	0.371	0.1273
3177	G	0.129	0.0042
3178	C	0.351	0.2510
3179	C	0.255	0.0651
3180	A	0.704	0.1421
3181	G	0.272	0.0665
3182	U	0.682	0.0262
3183	A	0.247	0.0841
3184	A	0.445	0.0339
3185	A	0.483	0.0071
3186	U	0.280	0.1018
3187	C	0.318	0.1612
3188	U	0.425	0.1442
3189	A	1.025	0.2638
3190	A	1.051	0.1294
3191	A	0.793	0.0834
3192	G	0.395	0.1230
3193	A	0.530	0.3486
3194	U	0.216	0.2029
3195	U	0.116	0.0771
3196	U	0.328	0.2546
3197	C	0.202	0.1421
3198	A	0.809	0.1874
3199	G	0.113	0.1421

3200	A	0.438	0.2581
3201	C	0.110	0.0771
3202	A	0.458	0.1761
3203	C	0.177	0.1520
3204	U	0.205	0.1138
3205	C	0.167	0.0488
3206	A	0.603	0.1987
3207	G	0.365	0.1838
3208	A	0.299	0.0000
3209	C	0.139	0.0000
3210	U	0.439	0.2814
3211	C	0.187	0.2425
3212	G	0.219	0.1803
3213	U	0.485	0.1379
3214	A	0.222	0.2107
3215	C	0.173	0.1690
3216	A	0.566	0.0396
3217	G	0.315	0.1937
3218	U	0.465	0.0742
3219	G	0.389	0.0325
3220	A	0.559	0.1598
3221	A	0.538	0.1520
3222	A	0.821	0.1478
3223	A	0.223	0.1039
3224	U	0.499	0.0127
3225	G	0.198	0.0445
3226	A	0.707	0.0834
3227	G	0.426	0.0559
3228	A	0.656	0.2150
3229	C	0.512	0.0721
3230	U	0.486	0.0820
3231	A	0.700	0.2489
3232	A	0.906	0.1322
3233	U	0.460	0.1563
3234	C	0.354	0.1209
3235	A	0.738	0.1754
3236	U	0.454	0.0856
3237	A	1.120	0.1124
3238	C	0.270	0.2715
3239	A	0.640	0.2567
3240	A	0.839	0.0000
3241	A	0.747	0.0849
3242	C	0.271	0.1633
3243	G	0.194	0.0544
3244	U	0.072	0.1004
3245	A	0.369	0.0368
3246	C	0.258	0.1541
3247	C	0.368	0.0389
3248	A	1.112	0.1831
3249	A	0.721	0.3585

3250	U	0.689	0.0346
3251	A	1.201	0.3861
3252	U	0.211	0.0721
3253	C	0.204	0.0346
3254	C	0.795	0.1435
3255	A	0.836	0.2864
3256	G	0.495	0.0714
3257	U	0.610	0.0764
3258	A	0.926	0.1619
3259	C	0.581	0.0177
3260	G	0.530	0.1237
3261	G	0.470	0.0134
3262	G	0.328	0.0495
3263	U	0.725	0.0000
3264	G	0.097	0.0651
3265	G	0.138	0.0057
3266	U	0.252	0.1103
3267	A	0.534	0.1598
3268	C	0.194	0.1966
3269	C	0.295	0.1372
3270	A	1.017	0.0948
3271	A	0.541	0.2553
3272	C	0.321	0.0460
3273	A	0.896	0.0000
3274	A	1.023	0.0106
3275	C	0.724	0.1047
3276	A	0.691	0.2496
3277	A	1.161	0.3175
3278	A	0.939	0.1810
3279	A	0.555	0.0368
3280	C	0.496	0.2164
3281	U	0.656	0.0276
3282	G	0.128	0.1655
3283	U	0.442	0.0304
3284	U	0.309	0.0438
3285	C	0.377	0.1365
3286	C	0.643	0.0021
3287	G	0.377	0.1110
3288	C	0.462	0.0354
3289	A	0.974	0.0064
3290	G	0.761	0.1216
3291	A	0.636	0.0212
3292	U	0.734	0.0255
3293	A	0.834	0.1301
3294	A	1.164	0.1075
3295	G	0.133	0.0997
3296	U	0.371	0.1570
3297	G	0.400	0.0148
3298	A	0.491	0.2737
3299	C	0.251	0.0000

3300	C	0.586	0.0933
3301	A	0.441	0.2574
3302	A	1.098	0.2079
3303	G	0.222	0.0000
3304	A	0.380	0.2623
3305	G	0.450	0.0375
3306	A	0.487	0.4568
3307	C	0.109	0.1294
3308	U	0.391	0.1308
3309	G	0.427	0.2956
3310	A	0.611	0.0127
3311	G	0.460	0.2751
3312	A	0.462	0.2942
3313	A	0.383	0.3868
3314	A	0.433	0.3769
3315	A	0.556	0.1718
3316	G	0.423	0.2595
3317	G	0.301	0.1853
3318	A	0.581	0.2270
3319	U	0.530	0.1640
3320	U	0.571	0.0742
3321	A	1.220	0.2206
3322	U	1.073	0.0042
3323	A	0.729	0.1372
3324	C	0.542	0.0806
3325	A	0.718	0.1591
3326	C	0.599	0.1817
3327	C	0.559	0.0629
3328	G	0.856	0.0205
3329	U	0.635	0.0834
3330	U	0.268	0.1089
3331	C	0.432	0.0163
3332	A	0.714	0.0933
3333	C	0.330	0.0269
3334	C	0.397	0.1450
3335	U	0.554	0.0134
3336	U	0.769	0.0622
3337	C	0.734	0.0113
3338	A	0.851	0.1442
3339	A	1.078	0.0000
3340	U	0.661	0.0990
3341	U	0.663	0.1018
3342	G	0.570	0.1223
3343	A	0.651	0.0516
3344	U	0.551	0.0870
3345	G	0.200	0.0587
3346	C	0.408	0.1259
3347	U	0.414	0.1358
3348	U	0.300	0.1096
3349	C	0.400	0.0481

3350	U	0.213	0.0332
3351	C	0.124	0.0233
3352	C	0.344	0.0127
3353	A	0.691	0.0700
3354	C	0.307	0.1287
3355	C	0.349	0.0000
3356	G	0.514	0.1541
3357	G	0.332	0.2058
3358	A	0.624	0.0255
3359	A	0.718	0.1223
3360	A	0.910	0.0127
3361	A	0.851	0.0389
3362	U	1.041	0.1025
3363	A	1.005	0.1188
3364	A	0.861	0.0092
3365	U	0.848	0.0163
3366	U	0.211	0.0219
3367	C	0.906	0.1372
3368	A	0.860	0.0134
3369	U	0.690	0.1287
3370	C	0.669	0.0940
3371	G	0.635	0.1315
3372	C	0.632	0.1174
3373	A	0.670	0.2440
3374	C	0.635	0.0870
3375	A	1.050	0.1308
3376	A	1.098	0.1386
3377	U	0.674	0.0410
3378	A	1.157	0.0000
3379	U	0.875	0.0290
3380	U	0.720	0.1054
3381	G	0.424	0.0134
3382	U	0.518	0.0304
3383	U	0.540	0.0255
3384	C	0.445	0.0198
3385	C	0.212	0.0728
3386	U	0.592	0.2376
3387	A	1.342	0.0000
3388	U	0.787	0.3620
3389	C	1.195	0.4306
3390	A	0.922	0.1619
3391	A	1.198	0.1909
3392	A	1.177	0.2461
3393	A	0.852	0.0877
3394	C	0.531	0.1683
3395	G	0.565	0.0099
3396	C	0.160	0.0601
3397	C	0.599	0.2835
3398	A	1.395	0.3818
3399	A	0.439	0.2461

3400	C	0.488	0.1230
3401	U	0.476	0.1287
3402	A	0.843	0.0000
3403	C	0.148	0.0530
3404	U	0.457	0.0905
3405	G	0.364	0.0820
3406	U	0.254	0.0919
3407	U	0.365	0.0078
3408	U	0.439	0.0035
3409	C	0.398	0.0955
3410	U	0.589	0.1492
3411	G	0.830	0.4243
3412	A	0.664	0.1732
3413	A	0.806	0.2977
3414	C	0.490	0.1973
3415	A	1.062	0.4646
3416	G	1.034	0.3932
3417	A	1.023	0.4681
3418	A	0.355	0.0191
3419	U	0.636	0.3196
3420	A	0.971	0.4504
3421	C	0.302	0.0962
3422	C	0.533	0.3239
3423	G	0.640	0.2277
3424	A	0.859	0.4582
3425	G	0.631	0.1131
3426	G	0.337	0.1351
3427	A	0.558	0.1704
3428	A	0.669	0.2744
3429	U	0.410	0.0410
3430	C	0.370	0.0856
3431	U	1.072	0.3606
3432	A	1.095	0.4151
3433	U	0.424	0.0658
3434	C	0.538	0.0629
3435	A	0.712	0.1061
3436	U	0.660	0.2022
3437	C	0.514	0.2864
3438	G	0.628	0.1838
3439	C	0.361	0.0707
3440	U	0.461	0.1039
3441	G	0.501	0.0940
3442	A	0.467	0.2220
3443	U	0.116	0.0064
3444	C	0.196	0.0156
3445	U	0.386	0.0438
3446	C	0.212	0.0438
3447	C	-	0.0000
3448	C	0.594	0.2616
3449	A	0.371	0.0145

3450	C	0.182	0.0057
3451	U	0.376	0.0810
3452	C	0.250	0.0615
3453	C	0.281	0.0506
3454	C	0.389	0.0781
3455	U	0.421	0.1092
3456	G	0.286	0.0346
3457	A	0.567	0.0665
3458	U	0.168	0.0230
3459	C	0.183	0.0000
3460	U	0.536	0.0633
3461	A	0.432	0.0131
3462	C	0.125	0.0583
3463	C	0.226	0.0064
3464	U	0.193	0.0124
3465	C	0.262	0.0156
3466	C	0.193	0.0216
3467	A	0.585	0.0926
3468	G	0.313	0.0407
3469	A	0.433	0.0495
3470	A	0.464	0.0470
3471	U	0.182	0.0170
3472	C	0.191	0.1764
3473	U	0.237	0.0456
3474	C	0.165	0.0725
3475	C	0.108	0.0782
3476	U	0.293	0.1051
3477	A	0.315	0.1214
3478	C	0.237	0.0154
3479	C	0.570	0.1608
3480	G	0.443	0.1490
3481	A	0.333	0.0460
3482	A	0.241	0.0690
3483	U	0.153	0.0396
3484	U	0.169	0.0777
3485	C	0.157	0.0621
3486	C	0.127	0.0900
3487	C	0.140	0.0682
3488	U	0.236	0.0921
3489	G	0.117	0.0100
3490	A	0.323	0.0486
3491	C	0.403	0.0835
3492	C	0.765	0.0601
3493	C	-	0.0000
3494	A	0.842	0.2233
3495	U	0.298	0.0759
3496	U	2.404	0.3004
3497	U	0.652	0.0689
3498	A	0.728	0.0577
3499	A	0.848	0.1562

3500	A	0.450	0.0408
3501	G	0.651	0.1166
3502	A	0.320	0.0531
3503	A	0.392	0.0323
3504	C	0.228	0.0283
3505	U	0.330	0.0255
3506	C	0.259	0.0619
3507	C	0.219	0.0212
3508	C	0.275	0.0481
3509	A	0.883	0.2785
3510	C	-	0.0000
3511	C	2.352	0.0000
3512	G	0.630	0.0375
3513	A	0.741	0.0668
3514	U	0.469	0.0743
3515	A	0.879	0.0691
3516	A	1.080	0.0120
3517	A	0.715	0.0663
3518	U	0.382	0.0033
3519	U	0.390	0.0308
3520	C	0.151	0.0403
3521	U	0.082	0.0378
3522	C	0.966	0.4125
3523	G	0.599	0.1518
3524	U	0.285	0.0918
3525	C	0.059	0.0290
3526	A	0.540	0.1608
3527	A	0.486	0.0110
3528	A	0.342	0.0667
3529	C	0.347	0.0420
3530	U	0.283	0.0292
3531	A	0.354	0.1920
3532	A	0.169	0.0757
3533	U	0.256	0.0856
3534	U	0.204	0.1790
3535	C	0.069	0.0010
3536	C	0.751	0.2871
3537	A	1.082	0.4503
3538	G	0.616	0.3005
3539	U	0.355	0.1485
3540	U	0.201	0.1510
3541	U	0.497	0.1860
3542	G	0.166	0.0439
3543	G	0.367	0.1376
3544	G	-	0.0000
3545	U	-	0.0000
3546	G	-	0.0000
3547	G	2.547	0.5063
3548	U	0.551	0.1514
3549	A	0.268	0.0289

3550	U	0.321	0.0543
3551	U	0.256	0.0642
3552	G	0.801	0.1652
3553	G	0.520	0.0771
3554	U	1.515	0.8499
3555	G	0.535	0.0651
3556	A	-	0.0000
3557	C	-	0.0000
3558	U	0.661	0.3173
3559	C	0.453	0.1641
3560	U	0.194	0.0641
3561	A	0.379	0.0957
3562	A	0.049	0.0100
3563	U	0.429	0.6140
3564	G	0.524	0.9023
3565	C	0.374	0.2390
3566	C	0.425	0.1319
3567	U	0.450	0.2070
3568	A	0.290	0.1794
3569	U	0.689	0.2241
3570	A	0.132	0.0685
3571	C	0.467	0.1236
3572	U	0.605	0.2143
3573	A	0.583	0.1833
3574	C	0.519	0.2022
3575	U	0.372	0.1242
3576	A	0.800	0.2649
3577	U	0.179	0.1605
3578	C	0.713	0.2076
3579	A	0.106	0.0262
3580	A	0.617	0.1410
3581	C	0.622	0.1346
3582	A	0.338	0.1104
3583	G	0.289	0.2326
3584	U	0.345	0.2034
3585	A	1.071	0.5554
3586	A	-	0.0000
3587	G	0.544	0.1168
3588	A	0.495	0.1802
3589	A	0.693	0.0250
3590	A	0.830	0.2501
3591	A	0.358	0.0145
3592	G	0.263	0.1250
3593	A	0.508	0.5798
3594	U	0.215	0.5501
3595	C	0.219	0.0397
3596	A	0.493	0.1703
3597	U	0.312	0.1752
3598	U	0.493	0.0991
3599	A	0.567	0.2092

3600	G	0.076	0.3797
3601	A	0.436	0.4985
3602	A	0.554	0.5170
3603	G	0.506	0.5182
3604	A	0.551	0.2191
3605	U	0.310	0.0277
3606	A	0.536	0.1732
3607	A	0.698	0.1563
3608	U	0.662	0.1743
3609	G	0.294	0.0459
3610	A	0.348	0.0345
3611	A	0.265	0.0438
3612	A	0.560	0.7799
3613	C	-	0.0000
3614	U	-	0.0000
3615	G	-	0.0000
3616	A	-	0.0000
3617	A	0.310	1.5669
3618	A	0.488	0.1371
3619	U	0.204	0.0311
3620	U	0.164	0.0965
3621	A	0.566	0.2343
3622	A	0.641	0.6102
3623	G	0.325	1.4949
3624	G	0.180	0.9695
3625	U	0.143	1.2113
3626	A	0.541	0.4458
3627	U	0.494	0.1574
3628	C	0.592	0.1244
3629	A	0.366	0.2944
3630	C	-	0.0000
3631	G	0.013	0.1647
3632	A	0.265	0.2428
3633	G	0.355	0.7499
3634	A	-	0.0000
3635	C	-	0.0000
3636	A	0.197	0.2532
3637	C	0.220	0.0494
3638	A	0.618	0.1050
3639	U	-	0.0000
3640	G	0.685	0.1753
3641	G	0.795	0.0857
3642	A	0.224	0.1030
3643	A	0.796	0.0529
3644	U	0.462	0.0630
3645	A	0.398	0.1315
3646	C	0.186	0.1035
3647	U	0.496	0.0634
3648	A	0.774	0.1617
3649	A	0.751	0.1287

3650	G	0.665	0.0448
3651	A	0.466	0.3220
3652	A	0.337	0.3220
3653	U	0.127	0.1168
3654	A	0.196	0.1648
3655	U	0.411	0.3953
3656	G	0.065	0.2333
3657	C	0.221	0.1431
3658	G	0.115	0.1427
3659	U	0.295	0.1520
3660	A	0.456	0.1821
3661	G	0.228	0.1313
3662	U	0.388	0.1862
3663	U	0.348	0.0645
3664	U	0.807	0.0771
3665	A	0.408	0.2191
3666	G	0.139	0.1620
3667	A	0.476	0.0858
3668	A	0.077	0.1593
3669	C	0.509	0.3015
3670	C	0.367	0.0732
3671	U	0.306	0.0902
3672	C	0.347	0.1662
3673	C	0.619	0.1788
3674	G	0.776	0.1134
3675	A	-	0.0000
3676	G	0.534	0.2315
3677	A	0.807	0.1407
3678	U	0.171	0.0429
3679	C	0.424	0.1436
3680	G	0.399	0.0739
3681	A	0.177	0.0598
3682	A	0.501	0.1900
3683	G	0.221	0.2761
3684	A	0.234	0.2226
3685	A	0.532	0.1777
3686	A	0.595	0.0933
3687	C	0.365	0.2270
3688	G	0.762	0.1083
3689	A	0.665	0.1475
3690	A	0.799	0.1249
3691	U	-	0.0000
3692	U	0.244	0.0574
3693	C	0.603	0.0410
3694	A	0.507	0.0735
3695	C	0.305	0.0446
3696	C	0.301	0.0827
3697	U	0.492	0.1045
3698	G	0.403	0.1139
3699	A	0.253	0.0460

3700	U	0.410	0.0972
3701	U	0.742	0.2271
3702	G	0.104	0.0177
3703	C	0.448	0.3058
3704	A	0.610	0.1859
3705	G	0.245	0.0429
3706	C	0.438	0.0737
3707	U	0.618	0.0396
3708	G	0.895	0.2265
3709	U	0.723	0.2430
3710	A	1.338	0.2092
3711	A	1.220	0.0811
3712	A	1.071	0.1132
3713	A	1.020	0.1202
3714	G	0.438	0.0923
3715	C	0.692	0.0926
3716	A	0.947	0.1079
3717	G	0.743	0.1709
3718	U	0.554	0.3021
3719	A	0.965	0.3798
3720	A	0.507	0.1738
3721	A	0.731	0.1663
3722	A	0.634	0.0456
3723	U	0.616	0.0387
3724	C	0.697	0.1208
3725	A	0.861	0.1940
3726	A	0.723	0.4296
3727	U	0.527	0.3095
3728	C	0.620	0.1930
3729	A	0.511	0.2180
3730	A	0.856	0.1319
3731	A	0.865	0.1426
3732	C	0.500	0.0751
3733	C	1.019	0.1536
3734	A	1.045	0.1643
3735	A	1.019	0.1949
3736	U	0.798	0.1100
3737	A	1.067	0.1046
3738	C	0.716	0.2105
3739	G	0.876	0.1617
3740	G	0.976	0.1609
3741	A	0.577	0.1199
3742	C	0.327	0.0802
3743	A	0.300	0.1248
3744	A	0.247	0.0842
3745	C	0.420	0.0715
3746	C	0.740	0.0159
3747	U	0.233	0.1137
3748	U	0.853	0.1560
3749	A	1.170	0.2629

3750	C	1.140	0.2972
3751	G	0.275	0.0921
3752	A	0.306	0.1008
3753	U	0.345	0.0788
3754	A	0.252	0.0638
3755	C	0.123	0.0952
3756	G	0.241	0.1922
3757	A	0.128	0.1078
3758	U	0.204	0.0486
3759	G	0.070	0.1959
3760	A	-	0.0000
3761	G	-	0.0000
3762	G	0.345	0.1397
3763	C	0.349	0.0634
3764	A	0.560	0.2258
3765	A	0.508	0.1182
3766	U	0.041	0.0414
3767	C	0.400	0.1523
3768	A	0.441	0.0945
3769	C	0.099	0.0373
3770	C	0.243	0.1068
3771	U	0.284	0.1495
3772	A	0.430	0.1836
3773	U	0.365	0.1097
3774	A	0.605	0.2127
3775	A	0.552	0.2288
3776	U	0.101	0.1178
3777	A	0.348	0.1767
3778	A	0.925	0.2441
3779	A	0.505	0.1262
3780	G	0.217	0.1916
3781	A	0.303	0.0988
3782	U	0.134	0.0604
3783	A	0.461	0.0642
3784	U	0.372	0.2519
3785	U	0.333	0.0826
3786	A	0.479	0.1029
3787	A	0.691	0.2064
3788	A	0.551	0.1941
3789	G	0.511	0.1966
3790	A	0.258	0.0948
3791	A	0.441	0.0894
3792	A	0.436	0.0436
3793	A	0.528	0.0225
3794	A	0.609	0.4263
3795	G	0.356	0.0934
3796	A	0.262	0.1055
3797	A	0.445	0.0983
3798	A	0.580	0.1253
3799	A	0.519	0.1194

3800	A	0.262	0.0860
3801	U	0.280	0.1216
3802	A	0.209	0.0413
3803	U	0.364	0.0919
3804	A	0.509	0.1217
3805	U	0.193	0.1033
3806	C	0.326	0.0642
3807	G	0.393	0.1039
3808	A	0.467	0.1425
3809	G	0.354	0.0944
3810	G	0.280	0.3530
3811	C	0.232	0.3403
3812	A	0.135	0.3312
3813	U	0.226	0.0891
3814	A	0.190	0.0453
3815	C	0.135	0.0387
3816	C	0.230	0.0816
3817	A	0.350	0.0557
3818	C	0.095	0.0618
3819	A	0.346	0.0782
3820	A	0.434	0.1434
3821	A	0.290	0.0717
3822	G	0.234	0.5292
3823	A	0.254	0.0461
3824	A	0.458	0.1086
3825	G	0.248	0.0737
3826	U	0.099	0.0712
3827	C	0.285	0.1269
3828	A	0.494	0.2444
3829	A	0.467	0.2197
3830	U	0.096	1.0534
3831	C	0.405	0.0671
3832	A	0.256	0.0461
3833	A	0.403	0.1705
3834	C	0.199	0.0697
3835	U	0.367	0.1377
3836	G	0.389	0.0539
3837	U	0.189	0.1380
3838	U	0.295	0.0754
3839	G	0.274	0.1870
3840	A	0.254	0.0666
3841	A	0.285	0.1263
3842	G	0.285	0.0384
3843	A	0.431	0.3835
3844	U	0.407	0.1787
3845	G	0.506	0.2529
3846	A	0.496	0.0923
3847	A	0.830	0.0372
3848	A	1.327	0.2136
3849	A	-	0.0000

3850	C	0.230	0.2086
3851	U	0.224	0.0535
3852	U	0.551	0.0776
3853	G	0.331	0.0504
3854	G	0.417	0.0106
3855	G	0.312	0.0567
3856	A	0.330	0.0820
3857	C	0.273	0.0510
3858	A	0.496	0.0468
3859	C	0.174	0.0191
3860	U	0.245	0.0638
3861	G	0.375	0.0408
3862	A	0.504	0.0707
3863	C	0.760	0.0773
3864	G	0.790	0.0309
3865	A	0.752	0.0476
3866	A	0.739	0.0938
3867	U	0.576	0.1713
3868	A	1.074	0.1178
3869	U	0.163	0.0930
3870	U	0.508	0.2323
3871	A	0.656	0.1199
3872	U	0.310	0.0156
3873	G	0.159	0.0937
3874	A	0.616	0.1774
3875	C	0.277	0.1755
3876	A	-	0.0000
3877	G	-	0.0000
3878	A	0.453	0.0665
3879	A	0.855	0.1755
3880	A	0.700	0.1417
3881	A	0.883	0.2474
3882	G	0.811	0.2883
3883	A	1.038	0.1964
3884	A	0.923	0.2119
3885	A	0.588	0.1060
3886	U	0.726	0.1077
3887	A	0.130	0.0576
3888	G	0.177	0.0391
3889	A	0.681	0.1623
3890	C	0.413	0.1751
3891	C	0.363	0.0366
3892	C	0.329	0.0672
3893	U	0.638	0.1011
3894	A	0.774	0.1272
3895	A	0.773	0.0599
3896	A	0.490	0.1724
3897	A	0.941	0.1583
3898	G	0.540	0.0793
3899	A	0.337	0.1959

3900	G	0.319	0.1334
3901	U	0.143	0.0530
3902	A	0.652	0.2024
3903	A	1.202	0.2311
3904	U	0.310	0.1319
3905	A	0.876	0.2345
3906	A	0.742	0.0468
3907	A	0.483	0.1548
3908	C	0.457	0.1311
3909	U	0.364	0.0390
3910	C	0.320	0.0667
3911	A	0.496	0.0895
3912	A	0.677	0.1365
3913	U	0.944	0.0763
3914	G	0.559	0.0497
3915	U	0.406	0.1013
3916	U	0.038	0.0379
3917	U	0.210	0.0569
3918	A	0.419	0.0961
3919	U	0.150	0.0635
3920	C	0.338	0.1340
3921	U	0.269	0.1717
3922	U	-	0.0000
3923	C	0.316	0.1761
3924	A	0.770	0.3005
3925	A	0.147	0.0643
3926	C	0.400	0.0697
3927	A	0.531	0.1168
3928	A	0.925	0.2106
3929	G	0.404	0.1383
3930	A	0.244	0.1369
3931	A	0.698	0.1533
3932	A	0.301	0.0649
3933	C	2.935	0.2788
3934	G	0.277	0.0485
3935	U	0.334	0.1162
3936	G	0.302	0.1891
3937	A	0.559	0.2233
3938	C	1.707	0.1520
3939	G	0.124	0.0134
3940	G	0.823	0.2682
3941	U	0.556	0.3492
3942	A	0.413	0.1549
3943	C	0.557	0.2558
3944	U	0.625	0.0757
3945	C	0.553	0.2290
3946	A	0.844	0.2550
3947	U	0.597	0.2171
3948	A	-	0.0000
3949	A	1.210	0.0057

3950	A	0.973	0.1134
3951	G	0.619	0.1486
3952	C	0.596	0.1446
3953	U	0.854	0.1353
3954	A	0.882	0.0540
3955	G	0.616	0.0727
3956	A	0.384	0.0245
3957	U	0.395	0.1404
3958	U	0.184	0.0211
3959	U	0.444	0.0564
3960	G	0.523	0.0580
3961	U	0.271	0.0755
3962	U	0.462	0.0367
3963	G	0.598	0.1655
3964	C	0.504	0.1359
3965	A	0.501	0.0690
3966	A	0.827	0.0356
3967	G	1.267	0.3012
3968	A	2.269	0.5710
3969	G	4.065	3.2937
3970	G	2.968	2.8973
3971	U	1.823	1.1288
3972	G	0.572	0.0014
3973	A	-	0.0000
3974	U	0.382	0.1741
3975	A	0.932	0.2198
3976	U	0.574	0.1712
3977	U	0.745	0.1584
3978	C	0.463	0.1199
3979	A	1.156	0.1228
3980	G	0.145	0.0536
3981	C	0.437	0.0421
3982	A	0.974	0.0909
3983	U	0.398	0.0643
3984	C	0.382	0.0360
3985	C	0.349	0.2259
3986	U	1.013	0.0837
3987	G	0.697	0.1695
3988	A	0.616	0.0956
3989	C	0.370	0.1015
3990	A	1.038	0.1145
3991	C	0.365	0.0556
3992	U	0.546	0.0815
3993	U	2.137	0.5962
3994	A	0.547	0.1046
3995	C	0.694	0.1029
3996	G	0.595	0.0857
3997	A	0.739	0.1421
3998	C	0.545	0.1119
3999	U	0.453	0.0842

4000	C	0.332	0.0716
4001	A	0.842	0.0396
4002	G	0.793	0.0821
4003	G	0.535	0.1681
4004	C	0.405	0.0384
4005	A	0.725	0.0680
4006	U	0.726	0.0934
4007	G	0.340	0.0897
4008	C	0.381	0.1478
4009	A	-	0.0000
4010	A	0.468	0.1428
4011	U	0.664	0.0346
4012	C	0.833	0.3436
4013	C	0.762	0.2116
4014	A	0.812	0.1255
4015	A	0.592	0.1178
4016	U	0.387	0.0731
4017	A	0.736	0.0225
4018	C	0.599	0.1595
4019	C	0.473	0.0841
4020	G	0.641	0.1357
4021	U	0.749	0.1350
4022	A	0.517	0.2115
4023	C	0.678	0.0644
4024	A	0.298	0.0281
4025	U	0.596	0.1672
4026	C	0.596	0.0382
4027	A	0.519	0.1723
4028	C	0.540	0.0439
4029	U	0.409	0.0770
4030	A	0.779	0.0180
4031	U	0.652	0.0503
4032	G	0.790	0.1805
4033	C	0.498	0.0879
4034	A	1.059	0.2148
4035	U	0.721	0.1027
4036	U	0.540	0.0451
4037	A	1.191	0.1268
4038	A	0.695	0.1209
4039	U	0.901	0.1097
4040	G	0.758	0.0891
4041	A	0.271	0.1021
4042	C	0.584	0.0116
4043	A	0.518	0.0676
4044	U	0.269	0.0925
4045	C	0.475	0.0720
4046	C	0.644	0.1246
4047	C	0.335	0.0304
4048	U	0.901	0.0540
4049	G	0.576	0.0458

4050	U	0.558	0.0456
4051	C	0.333	0.0453
4052	A	0.570	0.1039
4053	C	0.464	0.1040
4054	U	0.460	0.0362
4055	U	0.371	0.0162
4056	G	0.519	0.0981
4057	C	0.678	0.0265
4058	A	0.461	0.0879
4059	U	0.691	0.1217
4060	U	0.574	0.0318
4061	A	0.863	0.0855
4062	G	0.625	0.0497
4063	A	0.827	0.1486
4064	C	0.552	0.0570
4065	A	0.968	0.1198
4066	A	0.708	0.1409
4067	U	0.791	0.1970
4068	A	1.388	0.1837
4069	A	0.959	0.3199
4070	C	0.466	0.0930
4071	U	0.693	0.1615
4072	A	0.997	0.2573
4073	C	0.497	0.1502
4074	U	0.606	0.1342
4075	A	0.889	0.2601
4076	U	0.710	0.1603
4077	A	0.733	0.2428
4078	U	0.641	0.1814
4079	U	0.552	0.1152
4080	A	0.865	0.2082
4081	C	0.435	0.1151
4082	A	0.557	0.0946
4083	C	0.518	0.2273
4084	A	0.655	0.1008
4085	A	0.520	0.1158
4086	U	0.508	0.1405
4087	U	0.631	0.0631
4088	A	0.657	0.0231
4089	G	0.568	0.0643
4090	A	0.419	0.0697
4091	C	0.399	0.0454
4092	A	0.296	0.0765
4093	U	0.396	0.0950
4094	A	0.387	0.0939
4095	U	0.218	0.0404
4096	C	0.226	0.1448
4097	U	0.280	0.0852
4098	U	0.168	0.0626
4099	C	0.334	0.0815

4100	G	0.439	0.1214
4101	G	0.147	0.0081
4102	C	0.241	0.0835
4103	A	0.434	0.0792
4104	U	0.363	1.2258
4105	A	0.339	0.0230
4106	U	0.518	0.0841
4107	U	0.471	0.0750
4108	U	0.270	0.0166
4109	G	0.285	0.0758
4110	U	0.281	0.1123
4111	A	0.252	0.1414
4112	U	0.410	0.3363
4113	G	0.190	0.0472
4114	C	0.265	0.1248
4115	A	0.222	0.0644
4116	G	0.451	0.0782
4117	A	0.403	0.0593
4118	C	0.205	0.0521
4119	A	0.398	0.1311
4120	U	0.142	0.0468
4121	C	0.110	0.0616
4122	A	0.245	0.1195
4123	A	0.348	0.0735
4124	A	0.442	0.1503
4125	G	0.230	0.0547
4126	A	0.211	0.0209
4127	A	0.368	0.0590
4128	G	0.097	0.1110
4129	A	0.186	0.0722
4130	A	0.252	0.0519
4131	U	0.153	0.1148
4132	U	0.184	0.1044
4133	A	0.293	0.2603
4134	U	0.171	0.0651
4135	A	0.301	0.0028
4136	C	0.212	0.0643
4137	A	0.282	0.1409
4138	U	0.584	0.0494
4139	A	0.429	0.0545
4140	A	0.275	0.1229
4141	G	0.288	0.0959
4142	A	0.279	0.1128
4143	C	0.174	0.2931
4144	C	0.243	0.0810
4145	U	0.234	0.1234
4146	C	0.200	0.1001
4147	C	0.276	0.0817
4148	A	0.223	0.0198
4149	C	0.220	0.0021

4150	C	0.177	1.0200
4151	A	0.335	0.0863
4152	C	0.492	0.0909
4153	A	0.487	0.0813
4154	U	0.198	0.0902
4155	U	0.222	0.0131
4156	U	0.304	0.0438
4157	A	0.458	0.0672
4158	G	0.337	0.0477
4159	G	0.179	0.0088
4160	A	0.304	0.0431
4161	A	0.409	0.0841
4162	U	0.316	0.1068
4163	G	0.273	0.0598
4164	A	0.276	0.1001
4165	A	0.411	0.1163
4166	U	0.450	0.1160
4167	G	0.299	0.0647
4168	A	0.187	0.0000
4169	U	0.335	0.0854
4170	A	0.532	0.0385
4171	A	0.304	0.0669
4172	G	0.365	0.0053
4173	U	0.276	0.0640
4174	U	0.354	0.0150
4175	G	0.277	0.0660
4176	A	0.143	0.0014
4177	U	0.247	0.0502
4178	A	0.355	0.0468
4179	C	0.117	0.0483
4180	G	0.113	0.0368
4181	U	0.253	0.0402
4182	U	0.230	0.0241
4183	U	0.210	0.0126
4184	G	0.364	0.0420
4185	A	0.523	0.0668
4186	A	0.802	0.0846
4187	G	0.603	0.2038
4188	A	0.266	0.1071
4189	A	0.706	0.3596
4190	A	0.286	0.1843
4191	U	0.531	0.1223
4192	C	0.272	0.1374
4193	A	0.439	0.0627
4194	C	0.285	0.0196
4195	U	0.427	0.1423
4196	U	0.261	0.1046
4197	U	0.221	0.1254
4198	A	0.721	0.1361
4199	U	0.487	0.1886

4200	G	0.162	0.1282
4201	G	0.544	0.2847
4202	A	-	0.0000
4203	U	0.504	0.0721
4204	U	0.597	0.1059
4205	G	0.330	0.1301
4206	A	1.048	0.1259
4207	A	0.924	0.2065
4208	A	0.476	0.1467
4209	C	0.188	0.0578
4210	A	0.478	0.1389
4211	A	0.503	0.0911
4212	A	0.429	0.0635
4213	G	0.047	0.0608
4214	U	0.211	0.0505
4215	G	0.215	0.0728
4216	G	0.196	0.0570
4217	A	0.517	0.2305
4218	G	0.555	0.2399
4219	C	0.340	0.1067
4220	G	0.483	0.1354
4221	A	0.590	0.0917
4222	A	0.284	0.0365
4223	C	0.153	0.0554
4224	U	0.879	0.1042
4225	G	0.349	0.0750
4226	G	0.157	0.0726
4227	U	0.260	0.1351
4228	A	0.348	0.1158
4229	C	0.347	0.1967
4230	G	0.150	0.0346
4231	A	0.639	0.0304
4232	A	-	0.0000
4233	A	0.947	0.5589
4234	C	0.391	0.5631
4235	U	0.518	0.1229
4236	A	0.707	0.0104
4237	U	0.337	0.1051
4238	C	0.459	0.0634
4239	A	0.339	0.1428
4240	A	0.730	0.0886
4241	A	0.598	0.0807
4242	U	0.367	0.0930
4243	C	0.341	0.1038
4244	A	0.155	0.1099
4245	U	0.394	0.1285
4246	A	0.421	0.1780
4247	C	0.217	0.0495
4248	C	0.168	0.1013
4249	U	0.363	0.1548

4250	G	0.530	0.1419
4251	A	0.678	0.1500
4252	U	0.585	0.1721
4253	A	0.876	0.1933
4254	C	0.448	0.0885
4255	A	1.340	0.3008
4256	A	0.984	4.9179
4257	C	0.723	1.6263
4258	A	0.705	1.3468
4259	A	0.945	0.6389
4260	U	0.664	0.4539
4261	G	0.446	0.1373
4262	U	0.389	0.0159
4263	G	0.307	0.0499
4264	G	0.097	0.0546
4265	U	0.188	0.0552
4266	A	0.607	0.0524
4267	U	0.363	0.1429
4268	G	0.243	0.0548
4269	G	0.378	0.0225
4270	A	0.791	0.0569
4271	A	0.518	0.1504
4272	G	0.382	0.0911
4273	A	1.041	0.2284
4274	A	0.797	0.1769
4275	G	0.289	0.0929
4276	U	0.765	0.1099
4277	U	0.893	0.1678
4278	C	0.273	0.0350
4279	G	0.517	0.0802
4280	U	0.585	0.0659
4281	G	0.309	0.0437
4282	G	0.219	0.0569
4283	A	1.039	0.1060
4284	U	0.696	0.0570
4285	G	0.913	0.0201
4286	G	0.749	0.1341
4287	U	0.278	0.1060
4288	C	0.242	0.0896
4289	A	0.332	0.1448
4290	U	1.032	0.2699
4291	G	0.273	0.1726
4292	C	0.434	0.2186
4293	G	0.236	0.1094
4294	U	0.649	0.3262
4295	A	0.930	0.2803
4296	U	0.705	0.1499
4297	U	1.014	0.0337
4298	U	0.830	0.2262
4299	A	1.349	0.2057

4300	A	1.069	0.3200
4301	A	1.180	0.2674
4302	A	1.002	0.3419
4303	A	0.895	0.3475
4304	C	0.415	0.1556
4305	A	0.975	0.1203
4306	G	0.564	0.1171
4307	U	0.210	0.1170
4308	C	0.364	0.1094
4309	A	0.424	0.2139
4310	A	1.161	0.2604
4311	G	0.571	0.1914
4312	U	1.685	0.6069
4313	G	0.899	0.3709
4314	A	1.237	0.6507
4315	C	3.422	2.4095
4316	A	0.698	0.0956
4317	A	0.498	0.1727
4318	U	0.674	0.3283
4319	U	0.519	0.1779
4320	U	0.702	0.1140
4321	G	0.533	0.1099
4322	U	0.393	0.0843
4323	U	0.476	0.1194
4324	U	0.559	0.0490
4325	A	0.667	0.1793
4326	U	0.751	0.1025
4327	U	0.552	0.1336
4328	C	0.568	0.1097
4329	G	0.564	0.1709
4330	U	0.454	0.0831
4331	A	0.845	0.1556
4332	G	0.506	0.0101
4333	A	0.327	0.1476
4334	U	0.685	0.2032
4335	G	0.661	0.1465
4336	A	0.522	0.3722
4337	U	0.313	0.1768
4338	A	0.686	0.1520
4339	U	0.830	0.2109
4340	G	0.560	0.1110
4341	G	0.413	0.0903
4342	U	0.181	0.0596
4343	A	0.525	0.0624
4344	U	0.422	0.1280
4345	U	0.524	0.1764
4346	G	0.360	0.1019
4347	U	0.447	0.1569
4348	U	0.408	0.1826
4349	U	0.385	0.1475

4350	A	0.772	0.1325
4351	G	0.404	0.0571
4352	C	0.509	0.1855
4353	A	0.966	0.1146
4354	A	1.382	0.2664
4355	A	0.582	0.1322
4356	A	0.857	0.0330
4357	A	0.874	0.0801
4358	U	0.549	0.0992
4359	C	0.439	0.0060
4360	U	0.552	0.1278
4361	A	0.982	0.0775
4362	A	0.375	0.0796
4363	A	0.645	0.0804
4364	U	0.447	0.1498
4365	U	0.264	0.1544
4366	C	0.394	0.0620
4367	A	0.672	0.0468
4368	A	-	0.0000
4369	A	1.705	0.1021
4370	C	0.589	0.0716
4371	A	1.097	0.0883
4372	A	1.029	0.0353
4373	A	0.873	0.0414
4374	A	0.886	0.0357
4375	G	0.288	0.0482
4376	A	0.335	0.0516
4377	A	0.743	0.0438
4378	U	0.324	0.0332
4379	U	0.349	0.0815
4380	A	0.954	0.1696
4381	U	0.361	0.0790
4382	A	0.794	0.0889
4383	G	0.469	0.0791
4384	A	0.480	0.1165
4385	G	0.182	0.0284
4386	A	0.448	0.0520
4387	A	0.665	0.0735
4388	G	0.228	0.0140
4389	C	0.237	0.1311
4390	U	0.342	0.0822
4391	U	0.664	0.1198
4392	A	0.815	0.1306
4393	A	0.777	0.1460
4394	G	0.519	0.2494
4395	A	0.561	0.1370
4396	U	0.559	0.1238
4397	G	0.382	0.1741
4398	C	0.881	0.3326
4399	A	0.083	0.0606

4400	A	0.371	0.1082
4401	U	0.801	0.2607
4402	A	0.304	0.0413
4403	C	0.614	0.3486
4404	G	0.504	0.1280
4405	A	0.397	0.0475
4406	C	0.277	0.0424
4407	A	0.554	0.0348
4408	C	0.190	0.0540
4409	C	0.395	0.0382
4410	A	0.867	0.1222
4411	A	0.729	0.0684
4412	G	0.280	0.0078
4413	A	0.535	0.0316
4414	U	0.559	0.0683
4415	U	0.365	0.0259
4416	A	0.886	0.0581
4417	U	0.733	0.0558
4418	A	0.517	0.0760
4419	A	0.827	0.1169
4420	A	0.670	0.0740
4421	U	0.248	0.0179
4422	C	0.586	0.0393
4423	U	0.473	0.0429
4424	A	0.511	0.0130
4425	G	0.693	0.0328
4426	G	0.393	0.0432
4427	C	0.479	0.0029
4428	G	0.331	0.0485
4429	A	0.700	0.0796
4430	A	0.488	0.0256
4431	A	0.614	0.0428
4432	G	0.369	0.0263
4433	U	0.356	0.0015
4434	G	0.356	0.0246
4435	A	0.326	0.0589
4436	U	0.424	0.0605
4437	G	0.290	0.0406
4438	A	0.596	0.0707
4439	G	0.362	0.0217
4440	G	0.179	0.0413
4441	A	0.501	0.2568
4442	A	0.543	0.2062
4443	A	0.543	0.2306
4444	U	0.221	0.1255
4445	U	0.384	0.2103
4446	C	0.576	0.2167
4447	A	0.757	0.2906
4448	A	0.580	0.2533
4449	U	0.519	0.2319

4450	A	0.405	0.1008
4451	U	0.512	0.1392
4452	G	0.248	0.0293
4453	A	0.588	0.1872
4454	C	0.149	0.0618
4455	A	0.496	0.1240
4456	U	0.442	0.0837
4457	A	0.431	0.1035
4458	C	0.377	0.0671
4459	U	0.260	0.0352
4460	U	0.410	0.1040
4461	G	0.689	0.1613
4462	G	0.240	0.0651
4463	C	0.178	0.0192
4464	U	0.461	0.1717
4465	U	0.533	0.2097
4466	A	0.527	0.2127
4467	G	0.481	0.1422
4468	A	0.356	0.1600
4469	A	0.718	0.2360
4470	A	0.392	0.1288
4471	U	0.238	0.1234
4472	C	0.408	0.1706
4473	A	0.534	0.2858
4474	A	0.624	0.1690
4475	A	0.364	0.1475
4476	U	0.441	0.3124
4477	A	0.620	0.4732
4478	U	0.223	0.3578
4479	C	0.238	0.3525
4480	A	0.808	0.2333
4481	A	0.663	0.2132
4482	A	0.358	0.0247
4483	G	0.265	0.0279
4484	A	0.284	0.1078
4485	G	0.195	0.0237
4486	G	0.230	0.0417
4487	U	0.245	0.0859
4488	A	0.517	0.0778
4489	A	0.417	0.0279
4490	A	0.480	0.0007
4491	U	0.392	0.0375
4492	A	0.475	0.0156
4493	C	0.385	0.0187
4494	A	0.519	0.0046
4495	U	0.455	0.0067
4496	G	0.400	0.0191
4497	A	0.605	0.0134
4498	A	0.706	0.0619
4499	A	0.507	0.0619

4500	U	0.363	0.0187
4501	U	0.419	0.0499
4502	A	0.623	0.0849
4503	G	0.445	0.0209
4504	G	0.260	0.0552
4505	U	0.124	0.0011
4506	A	0.441	0.0000
4507	U	0.558	0.2864
4508	G	0.734	0.3726
4509	G	0.616	0.1584
4510	A	0.215	0.0728
4511	A	0.738	0.1768
4512	A	0.568	0.0134
4513	A	0.405	0.1237
4514	C	0.128	0.0262
4515	U	0.324	0.0283
4516	C	0.277	0.0290
4517	A	0.783	0.1973
4518	U	-	0.0000
4519	U	0.354	0.0948
4520	A	1.076	0.1132
4521	A	0.718	0.1024
4522	C	0.149	0.0359
4523	U	0.534	0.0950
4524	G	0.363	0.1465
4525	A	0.422	0.0878
4526	G	0.546	0.0482
4527	A	0.753	0.1510
4528	A	0.837	0.4501
4529	A	0.544	0.2679
4530	A	0.218	0.1406
4531	U	0.426	0.1580
4532	A	0.513	0.1612
4533	C	0.227	0.0810
4534	C	0.154	0.0713
4535	C	0.250	0.0731
4536	A	0.501	0.1585
4537	A	0.537	0.1005
4538	A	0.404	0.1142
4539	U	0.614	0.1606
4540	U	0.467	0.2206
4541	A	0.567	0.3147
4542	A	0.455	0.1600
4543	A	0.449	0.1815
4544	C	0.450	0.2772
4545	G	-	0.0000
4546	U	0.181	0.0668
4547	A	0.385	0.1188
4548	C	0.188	0.1099
4549	C	0.181	0.0710

4550	U	0.171	0.0831
4551	U	-	0.0000
4552	U	0.437	0.1003
4553	G	0.447	0.0686
4554	A	0.307	0.0255
4555	A	0.431	0.0800
4556	U	0.386	0.1049
4557	C	0.316	0.0419
4558	C	0.288	0.0286
4559	A	0.416	0.1415
4560	A	0.545	0.1512
4561	A	0.607	0.0826
4562	A	0.772	0.1093
4563	G	0.349	0.1306
4564	G	0.695	0.2780
4565	A	1.127	0.4491
4566	A	0.943	0.2065
4567	G	0.734	0.1080
4568	A	0.089	0.0933
4569	A	0.570	0.0785
4570	A	0.649	0.0963
4571	A	0.398	0.0308
4572	C	0.305	0.0306
4573	U	0.269	0.0465
4574	U	0.788	0.0720
4575	A	0.562	0.0242
4576	G	0.343	0.0216
4577	C	0.206	0.2018
4578	G	0.255	0.0847
4579	C	0.236	0.1527
4580	U	0.224	0.1768
4581	C	0.142	0.0523
4582	C	0.346	0.0992
4583	A	0.644	0.1481
4584	G	0.760	0.1751
4585	G	0.479	0.1256
4586	U	0.095	0.0764
4587	C	0.164	0.0516
4588	A	0.277	0.1057
4589	A	0.363	0.0621
4590	C	0.286	0.0679
4591	C	1.258	0.4278
4592	A	0.270	0.1421
4593	G	-	0.0000
4594	G	-	0.0000
4595	U	0.865	0.2605
4596	C	0.269	0.1143
4597	U	0.236	0.1040
4598	U	0.214	0.0845
4599	U	0.420	0.2262

4600	A	1.603	0.2135
4601	U	0.235	0.1175
4602	A	0.523	0.0025
4603	U	0.417	0.0595
4604	A	0.332	0.1021
4605	G	0.237	0.0904
4606	A	0.199	0.0758
4607	C	0.155	0.1204
4608	C	0.607	0.0569
4609	A	1.011	0.1808
4610	G	1.746	0.0438
4611	G	0.895	0.1433
4612	A	0.578	0.1396
4613	U	0.105	0.0526
4614	G	0.690	0.1562
4615	A	0.421	0.1161
4616	A	0.215	0.0962
4617	C	0.423	0.1203
4618	U	0.466	0.1346
4619	A	0.506	0.0975
4620	G	0.817	0.1121
4621	A	0.394	0.0250
4622	A	0.376	0.0656
4623	A	0.584	0.0583
4624	U	0.515	0.1645
4625	A	0.652	0.1020
4626	G	1.037	0.0764
4627	A	0.617	0.0341
4628	U	0.425	0.0829
4629	G	0.720	0.0096
4630	A	0.535	0.0670
4631	A	0.647	0.1796
4632	G	0.706	0.0822
4633	A	0.417	0.0389
4634	U	0.483	0.0425
4635	G	0.352	0.1742
4636	A	0.212	0.1018
4637	A	0.413	0.0773
4638	U	0.274	0.0975
4639	A	0.641	0.0903
4640	C	0.599	0.1834
4641	A	0.808	0.0990
4642	A	0.941	0.1465
4643	A	-	0.0000
4644	G	0.687	0.1288
4645	A	0.302	0.0705
4646	G	0.179	0.0830
4647	A	0.218	0.0512
4648	A	-	0.0000
4649	G	-	0.0000

4650	G	-	0.0000
4651	U	-	0.0000
4652	A	-	0.0000
4653	C	-	0.0000
4654	A	0.227	0.0626
4655	U	0.524	0.1240
4656	G	0.516	0.0260
4657	A	0.150	0.1744
4658	A	0.473	0.2147
4659	A	-	0.0000
4660	U	-	0.0000
4661	G	-	0.0000
4662	C	-	0.0000
4663	A	-	0.0000
4664	A	-	0.0000
4665	A	0.662	0.0820
4666	A	0.563	0.0432
4667	G	0.151	0.1382
4668	U	0.180	0.0707
4669	U	0.341	0.1502
4670	G	0.313	0.1301
4671	A	0.074	0.0163
4672	U	0.392	0.0728
4673	U	0.360	0.2242
4674	G	0.522	0.0163
4675	G	0.263	0.0233
4676	U	0.148	0.0219
4677	C	0.155	0.0548
4678	U	0.349	0.1002
4679	A	0.724	0.0878
4680	G	0.297	0.1031
4681	C	0.460	0.0486
4682	U	0.573	0.0794
4683	U	0.580	0.1069
4684	C	0.576	0.1944
4685	A	0.456	0.1296
4686	U	0.391	0.1501
4687	A	0.820	0.1481
4688	U	0.388	0.1563
4689	G	0.319	0.0959
4690	U	0.240	0.0580
4691	U	0.475	0.1017
4692	G	0.251	0.0537
4693	G	0.202	0.0735
4694	A	0.505	0.1899
4695	U	0.319	0.2059
4696	A	0.994	0.2021
4697	U	0.278	0.1441
4698	A	1.096	0.0789
4699	A	-	0.0000

4700	A	1.274	0.2043
4701	U	0.189	0.0519
4702	U	0.325	0.0812
4703	U	0.464	0.2110
4704	A	0.884	0.0966
4705	G	0.870	0.2545
4706	A	0.999	0.9705
4707	U	0.746	0.3601
4708	U	0.213	0.2060
4709	U	0.589	0.1703
4710	G	0.790	0.0912
4711	A	1.010	0.0318
4712	C	0.273	0.0320
4713	U	0.837	0.0531
4714	U	0.811	0.0658
4715	A	0.931	0.0707
4716	C	0.591	0.1631
4717	U	0.372	0.0989
4718	A	0.913	0.1275
4719	U	0.959	0.0737
4720	A	0.552	0.0988
4721	C	0.485	0.0394
4722	U	0.884	0.0362
4723	A	0.672	0.1404
4724	C	0.528	0.0594
4725	A	1.042	0.0728
4726	U	0.486	0.0689
4727	C	0.863	0.0287
4728	A	0.763	0.1000
4729	A	0.916	0.1487
4730	C	0.401	0.0307
4731	A	0.851	0.1144
4732	C	0.508	0.1158
4733	A	0.946	0.1187
4734	C	0.453	0.1595
4735	U	0.741	0.1820
4736	U	0.716	0.1231
4737	G	0.571	0.1534
4738	C	0.654	0.2046
4739	U	0.481	0.0900
4740	C	0.956	0.2770
4741	A	0.894	0.2322
4742	A	0.962	0.1850
4743	C	0.593	0.0951
4744	A	0.917	0.1210
4745	U	0.492	0.0429
4746	A	0.899	0.0944
4747	U	0.681	0.0938
4748	A	0.821	0.0709
4749	C	0.551	0.0254

4750	U	0.819	0.0466
4751	A	0.713	0.1826
4752	U	0.532	0.0539
4753	U	0.384	0.0254
4754	C	0.475	0.0872
4755	C	0.261	0.0300
4756	C	0.527	0.1355
4757	C	0.611	0.1351
4758	U	0.521	0.1862
4759	C	0.572	0.1746
4760	U	0.419	0.1074
4761	A	0.893	0.1813
4762	G	0.670	0.1522
4763	G	0.210	0.0708
4764	C	0.366	0.1385
4765	A	0.601	0.1989
4766	A	0.650	0.1591
4767	G	0.748	0.1609
4768	U	0.521	0.2318
4769	U	0.345	0.0734
4770	U	0.705	0.2596
4771	U	0.702	0.2158
4772	A	0.980	0.3122
4773	G	0.807	0.1666
4774	A	0.569	0.1467
4775	C	0.722	0.2731
4776	A	0.560	0.1811
4777	U	0.526	0.1093
4778	G	0.479	0.1034
4779	A	0.533	0.1151
4780	C	0.439	0.1534
4781	A	0.579	0.2014
4782	U	0.473	0.1670
4783	A	0.304	0.1273
4784	U	0.517	0.2338
4785	G	0.494	0.1706
4786	A	0.369	0.1187
4787	G	0.345	0.2162
4788	U	0.372	0.1804
4789	U	0.454	0.2651
4790	G	0.412	0.2168
4791	A	0.583	0.2671
4792	U	0.395	0.1797
4793	A	0.820	0.2962
4794	C	0.550	0.1713
4795	A	0.604	0.2326
4796	A	0.855	0.4144
4797	U	0.616	0.2701
4798	U	0.476	0.1762
4799	C	0.659	0.2366

4800	A	0.551	0.2521
4801	U	0.435	0.1926
4802	G	0.275	0.0738
4803	U	0.519	0.1735
4804	G	0.257	0.0801
4805	G	0.288	0.0827
4806	G	0.235	0.0357
4807	A	0.601	0.1182
4808	C	0.368	0.0990
4809	A	0.402	0.1068
4810	C	0.272	0.0924
4811	U	0.237	0.0627
4812	A	0.507	0.0853
4813	G	0.326	0.1172
4814	A	0.548	0.1627
4815	G	0.350	0.1069
4816	A	0.612	0.2279
4817	U	0.461	0.1763
4818	A	0.495	0.1490
4819	A	0.751	0.3859
4820	A	0.249	0.1816
4821	C	0.216	0.0852
4822	A	0.352	0.1760
4823	A	0.417	0.1484
4824	C	0.227	0.1011
4825	U	0.289	0.1589
4826	G	0.140	0.0035
4827	A	0.280	0.0106
4828	U	0.231	0.0906
4829	A	0.244	0.1039
4830	U	0.305	0.1824
4831	G	0.281	0.1253
4832	G	0.229	0.0413
4833	C	0.196	0.0954
4834	A	0.389	0.0886
4835	C	0.240	0.0096
4836	A	0.473	0.2179
4837	A	0.514	0.0941
4838	A	0.507	0.0679
4839	A	0.481	0.1579
4840	A	0.478	0.1001
4841	C	0.381	0.1250
4842	A	0.345	0.0759
4843	A	0.524	0.0825
4844	A	0.494	0.0342
4845	C	0.183	0.0457
4846	C	0.116	0.0211
4847	U	0.274	0.0318
4848	A	0.381	0.0541
4849	C	0.087	0.0606

4850	C	0.332	0.1912
4851	G	0.248	0.2468
4852	A	0.328	0.2732
4853	G	0.250	0.2242
4854	C	0.168	0.0821
4855	C	0.102	0.0453
4856	A	0.119	0.0801
4857	G	0.090	0.1537
4858	A	-	0.0000
4859	U	0.272	0.2037
4860	A	0.187	0.0552
4861	A	0.424	0.0895
4862	U	0.589	0.3114
4863	A	0.891	0.2265
4864	A	1.306	0.0781
4865	A	0.744	0.1599
4866	C	0.402	0.0448
4867	U	0.637	0.0454
4868	A	-	0.0000
4869	G	-	0.0000
4870	U	-	0.0000
4871	C	-	0.0000
4872	G	-	0.0000
4873	C	-	0.0000
4874	A	-	0.0000
4875	A	-	0.0000
4876	U	-	0.0000
4877	A	-	0.0000
4878	A	-	0.0000
4879	G	-	0.0000
4880	U	-	0.0000
4881	G	-	0.0000
4882	A	-	0.0000
4883	U	0.450	0.1221
4884	G	0.471	0.0312
4885	C	0.016	0.0132
4886	U	-	0.0000
4887	U	0.165	0.1024
4888	C	0.225	0.2674
4889	G	0.527	0.1604
4890	U	0.168	0.0864
4891	A	0.507	0.0251
4892	U	0.243	0.2173
4893	G	0.249	0.1231
4894	G	0.116	0.0500
4895	C	0.047	0.1325
4896	A	-	0.0000
4897	A	-	0.0000
4898	C	0.240	0.2247
4899	C	0.455	0.5658

4900	A	-	0.0000
4901	A	0.403	0.1722
4902	C	0.248	0.0305
4903	C	0.266	0.0817
4904	G	0.192	0.0156
4905	U	0.180	0.0237
4906	A	0.304	0.0506
4907	U	0.057	0.2621
4908	U	-	0.0000
4909	A	-	0.0000
4910	U	-	0.0000
4911	A	-	0.0000
4912	A	-	0.0000
4913	A	-	0.0000
4914	U	-	0.0000
4915	C	-	0.0000
4916	A	-	0.0000
4917	C	0.106	0.2743
4918	A	0.256	0.3019
4919	A	0.223	0.2526
4920	A	0.200	0.1763
4921	U	0.220	0.3862
4922	U	-	0.0000
4923	G	0.153	0.0000
4924	G	0.461	0.0000
4925	C	0.238	0.0000
4926	A	-	0.0000
4927	A	0.301	0.0406
4928	C	0.743	0.0752
4929	A	0.209	0.1074
4930	U	0.307	0.0718
4931	A	0.082	0.5357
4932	U	0.369	0.2859
4933	A	0.297	0.0839
4934	U	0.235	0.1175
4935	U	0.067	0.0209
4936	U	0.090	0.1942
4937	A	0.283	0.0841
4938	C	0.143	0.0358
4939	U	0.230	0.1064
4940	U	0.232	0.1382
4941	A	0.147	0.0109
4942	A	0.304	0.0594
4943	U	0.191	0.0696
4944	G	0.028	0.0877
4945	G	0.031	0.0162
4946	A	0.613	0.0845
4947	A	0.350	0.0872
4948	A	2.305	0.0317
4949	G	0.289	0.0463

4950	G	0.285	0.0545
4951	U	0.312	0.0431
4952	A	0.075	0.1352
4953	A	0.334	0.6771
4954	U	0.201	0.0700
4955	U	0.332	0.0225
4956	G	0.099	0.0365
4957	G	0.892	0.0454
4958	A	-	0.0000
4959	G	0.359	0.1655
4960	G	0.075	0.0639
4961	A	0.318	0.0340
4962	A	1.060	0.0217
4963	A	0.350	0.0272
4964	G	0.676	0.0211
4965	U	0.552	0.0666
4966	C	0.123	0.0410
4967	C	2.176	0.2723
4968	A	1.906	1.3484
4969	C	0.435	1.0905
4970	C	0.319	0.5321
4971	A	1.521	0.2132
4972	A	1.941	0.1489
4973	G	0.667	0.1526
4974	G	-	0.0000
4975	C	1.732	0.0514
4976	U	0.708	0.1011
4977	U	0.345	0.0727
4978	C	0.141	0.0148
4979	A	0.561	0.0599
4980	U	0.433	0.0615
4981	U	0.025	0.0558
4982	A	0.090	0.0940
4983	A	0.541	0.0109
4984	C	0.345	0.2108
4985	A	0.682	0.0349
4986	U	0.501	0.0184
4987	G	0.008	0.0395
4988	U	0.278	0.0746
4989	A	0.019	0.0195
4990	C	0.083	0.0696
4991	U	0.066	0.0584
4992	U	0.122	0.0638
4993	C	-	0.0000
4994	A	0.542	0.1066
4995	A	0.202	0.0667
4996	C	0.631	0.1247
4997	U	0.461	0.1109
4998	A	0.390	0.1250
4999	C	0.393	0.1107

5000	G	0.614	0.0146
5001	G	1.794	0.1097
5002	A	1.575	0.0805
5003	A	2.085	0.0826
5004	G	1.618	0.0456
5005	C	-	0.0000
5006	A	1.943	0.0807
5007	G	0.869	0.0469
5008	A	0.161	0.0619
5009	A	0.349	0.1150
5010	A	0.340	0.0299
5011	U	0.405	0.0576
5012	A	0.360	0.0871
5013	C	0.789	0.0084
5014	A	0.439	0.0395
5015	C	0.437	0.0768
5016	G	0.517	0.0706
5017	C	0.525	0.2353
5018	G	0.466	0.4474
5019	A	0.473	0.2030
5020	U	0.630	0.2788
5021	A	1.748	0.1867
5022	A	0.935	0.1463
5023	G	1.188	0.1838
5024	U	0.701	0.1765
5025	G	0.639	0.0285
5026	A	0.899	0.0775
5027	A	1.519	0.0196
5028	U	0.404	0.1839
5029	C	0.501	0.0733
5030	U	0.208	0.0997
5031	G	0.557	0.1324
5032	U	0.405	0.1484
5033	C	0.227	0.0367
5034	C	0.107	0.0313
5035	C	0.511	0.0142
5036	A	0.341	0.0810
5037	U	-	0.0000
5038	U	0.251	0.1397
5039	A	0.415	0.0989
5040	U	0.442	0.0983
5041	U	1.198	0.1813
5042	A	0.542	0.3415
5043	A	0.342	0.0863
5044	A	0.190	0.0742
5045	U	0.339	0.4681
5046	A	0.411	0.1082
5047	A	0.225	0.1294
5048	U	-	0.0000
5049	C	0.738	0.1556

5050	U	0.528	0.0134
5051	A	0.710	0.1612
5052	A	0.514	0.2680
5053	G	0.157	0.1655
5054	U	0.034	0.0049
5055	U	0.171	0.0000
5056	A	0.348	0.1541
5057	C	0.131	0.1138
5058	C	0.239	0.0000
5059	U	0.358	0.0488
5060	G	0.096	0.0000
5061	A	0.482	0.1612
5062	U	0.254	0.0700
5063	A	0.172	0.1775
5064	C	0.278	0.0021
5065	A	0.480	0.0700
5066	A	0.519	0.1315
5067	G	0.471	0.1039
5068	A	0.378	0.0000
5069	A	0.577	0.1365
5070	C	0.701	0.0000
5071	U	0.416	0.0007
5072	U	0.651	0.3231
5073	A	0.645	0.1980
5074	A	0.514	0.0431
5075	C	0.511	0.1351
5076	A	0.614	0.2934
5077	A	0.459	0.0071
5078	G	0.452	0.2623
5079	A	0.299	0.1365
5080	A	0.285	0.0240
5081	A	0.236	0.2390
5082	C	-	0.0000
5083	C	-	0.0000
5084	A	-	0.0000
5085	A	0.331	0.2355
5086	U	0.308	0.2779
5087	U	0.466	0.0290
5088	A	0.760	0.0912
5089	U	0.720	0.0318
5090	U	0.323	0.1011
5091	A	0.407	0.2560
5092	A	0.392	0.0445
5093	A	0.594	0.0042
5094	G	0.660	0.2934
5095	G	0.374	0.2949
5096	C	0.262	0.1280
5097	U	0.395	0.4327
5098	U	0.267	0.0000
5099	A	0.581	0.1584

5100	C	0.318	0.1358
5101	U	0.447	0.1110
5102	U	0.386	0.0000
5103	A	0.592	0.5346
5104	C	0.539	0.0000
5105	U	0.475	0.0693
5106	G	0.568	0.5190
5107	A	0.319	0.2291
5108	U	0.324	0.0000
5109	A	0.400	0.2022
5110	G	0.935	0.2397
5111	U	0.731	0.1146
5112	A	0.911	0.2107
5113	G	0.367	0.1131
5114	A	0.701	0.1237
5115	U	0.443	0.0000
5116	C	0.522	0.1138
5117	A	0.701	0.2687
5118	A	0.562	0.1626
5119	C	0.541	0.1980
5120	G	0.519	0.0000
5121	A	0.372	0.0764
5122	U	0.168	0.5827
5123	C	0.235	0.0000
5124	A	0.380	0.2574
5125	G	0.521	0.0608
5126	U	0.258	0.0205
5127	A	0.666	0.3111
5128	U	0.681	0.2312
5129	A	0.793	0.2029
5130	A	0.742	0.2348
5131	U	0.464	0.0000
5132	U	0.507	0.0014
5133	A	0.429	0.2432
5134	A	0.266	0.0834
5135	G	0.203	0.2567
5136	U	0.198	0.0396
5137	C	0.315	0.2312
5138	U	0.502	0.0000
5139	A	0.538	0.0000
5140	C	0.367	0.0495
5141	A	0.427	0.2546
5142	A	0.424	0.3366
5143	A	0.384	0.1506
5144	U	0.483	0.2835
5145	G	0.279	0.2496
5146	A	1.518	0.0502
5147	A	0.815	0.1831
5148	G	0.760	0.1450
5149	A	0.584	0.3090

5150	G	0.444	0.3642
5151	A	0.426	0.2751
5152	A	0.514	0.1223
5153	A	0.535	0.0000
5154	U	0.380	0.5289
5155	U	0.378	0.2100
5156	U	0.525	0.0573
5157	A	0.939	0.0000
5158	G	0.672	0.0000
5159	A	0.527	0.3507
5160	A	0.493	0.4342
5161	A	0.507	0.2800
5162	C	0.385	0.3528
5163	A	0.586	0.1294
5164	G	0.410	0.2765
5165	A	0.382	0.0000
5166	U	0.495	0.0000
5167	U	0.408	0.0000
5168	U	0.484	0.0000
5169	U	0.494	0.0672
5170	U	0.720	0.1322
5171	U	0.574	0.0658
5172	G	0.574	0.0785
5173	G	0.536	0.3359
5174	C	0.672	0.1994
5175	A	0.685	0.0354
5176	C	0.772	0.1245
5177	A	0.592	0.0000
5178	A	0.428	0.0000
5179	A	0.853	0.1153
5180	G	0.530	0.0849
5181	G	0.436	0.0912
5182	C	0.243	0.4320
5183	A	0.446	0.1287
5184	A	0.373	0.1075
5185	U	0.423	0.1379
5186	G	0.345	0.0438
5187	A	0.524	0.2029
5188	G	0.477	0.2418
5189	A	0.404	0.4236
5190	C	0.382	0.3076
5191	U	0.357	0.1372
5192	U	0.260	0.2687
5193	A	0.493	0.2065
5194	G	0.215	0.0566
5195	A	0.513	0.2143
5196	G	0.502	0.0990
5197	A	0.581	0.0269
5198	U	0.420	0.1909
5199	G	0.360	0.1923

5200	A	0.487	0.0000
5201	A	0.474	0.0000
5202	G	0.324	0.7234
5203	U	0.409	0.6583
5204	A	0.385	0.1414
5205	U	0.109	0.0467
5206	C	0.477	0.0594
5207	A	0.706	0.3041
5208	G	0.464	0.3472
5209	G	0.495	0.0658
5210	U	0.191	0.1819
5211	A	0.411	0.1397
5212	A	0.464	0.3845
5213	U	0.412	0.0000
5214	A	0.590	0.6293
5215	A	0.679	0.6611
5216	U	0.747	0.0416
5217	U	0.850	0.4532
5218	U	0.503	0.1241
5219	A	0.413	0.0000
5220	U	0.372	0.1276
5221	A	0.539	0.3239
5222	C	0.537	0.0514
5223	G	0.552	0.0843
5224	U	0.418	0.2921
5225	A	0.471	0.0040
5226	U	0.606	0.1264
5227	A	0.355	0.1940
5228	C	0.385	0.0491
5229	U	0.731	0.5866
5230	A	0.131	0.1830
5231	C	0.188	0.0479
5232	A	0.561	0.2477
5233	U	0.435	0.3054
5234	C	0.610	0.2315
5235	G	0.593	0.2332
5236	A	0.631	0.2939
5237	G	0.946	0.1334
5238	A	0.772	0.1882
5239	C	0.135	0.0410
5240	C	0.545	0.0658
5241	A	0.660	0.2748
5242	A	0.580	0.5046
5243	G	0.357	0.2055
5244	A	0.565	0.3828
5245	A	0.736	0.1189
5246	G	0.693	0.3060
5247	A	0.724	0.1749
5248	A	0.808	0.3741
5249	C	0.445	0.0554

5250	A	0.599	0.1876
5251	U	0.098	0.0277
5252	U	0.398	0.0006
5253	G	0.688	0.0254
5254	C	0.356	0.0000
5255	U	0.325	0.2107
5256	G	0.580	0.0098
5257	A	0.333	0.0323
5258	U	0.488	0.0318
5259	G	0.620	0.1380
5260	U	0.515	0.0000
5261	G	0.295	0.0548
5262	A	0.956	0.3799
5263	U	0.429	0.2858
5264	G	0.376	0.0179
5265	A	0.435	0.1744
5266	C	0.523	0.1738
5267	A	0.356	0.0173
5268	A	0.784	0.0849
5269	A	0.596	0.3222
5270	A	0.916	0.2217
5271	C	0.403	0.1553
5272	C	0.509	0.2789
5273	U	0.953	0.3597
5274	C	0.552	0.2460
5275	U	1.159	1.1749
5276	U	0.316	0.1068
5277	C	0.582	0.3914
5278	C	0.312	0.0098
5279	G	0.721	0.0000
5280	A	0.623	0.4388
5281	U	1.308	1.3498
5282	A	1.061	0.9803
5283	A	1.115	0.8216
5284	A	0.685	0.3262
5285	A	0.575	0.5606
5286	A	0.895	0.5283
5287	C	1.439	0.3868
5288	A	-	0.0000
5289	U	0.964	0.1871
5290	U	1.002	0.4498
5291	U	0.606	0.0254
5292	A	0.900	0.1374
5293	A	0.914	0.6022
5294	A	0.855	0.8268
5295	C	0.571	0.2500
5296	U	0.534	0.0023
5297	A	0.781	0.2731
5298	U	0.607	0.6801
5299	U	0.226	0.1039

5300	A	0.718	0.0000
5301	A	0.657	0.7309
5302	C	0.678	0.5797
5303	U	-	0.0000
5304	A	0.600	0.3753
5305	A	0.953	0.6830
5306	C	0.719	0.3730
5307	A	0.287	0.1120
5308	A	0.536	0.4861
5309	A	0.737	0.0000
5310	U	0.593	0.0000
5311	G	0.738	0.0000
5312	G	0.826	0.0256
5313	A	0.160	0.1547
5314	U	0.337	0.1420
5315	U	0.252	0.2102
5316	C	0.498	0.3239
5317	A	0.516	0.1380
5318	U	0.452	0.3441
5319	U	0.328	0.0981
5320	A	0.514	0.3170
5321	G	0.224	0.1005
5322	A	0.220	0.0023
5323	U	0.514	0.0658
5324	C	0.363	0.2633
5325	U	0.036	0.0000
5326	A	0.214	0.0277
5327	U	0.467	0.4070
5328	C	0.403	0.4157
5329	G	0.432	0.3620
5330	A	0.290	0.1045
5331	U	0.448	0.3066
5332	A	0.530	0.1276
5333	G	0.254	0.2679
5334	A	0.413	0.0219
5335	U	0.459	0.1726
5336	C	0.295	0.1149
5337	U	0.109	0.1039
5338	A	0.620	0.2524
5339	U	0.199	0.1351
5340	U	0.218	0.0149
5341	A	0.378	0.2027
5342	C	0.692	0.1424
5343	A	0.266	0.1640
5344	U	0.545	0.2418
5345	U	0.219	0.2084
5346	A	0.263	0.1253
5347	U	0.488	0.3679
5348	G	0.875	0.4772
5349	G	0.288	0.1171

5350	G	0.111	0.0554
5351	U	0.217	0.0141
5352	G	0.473	0.4050
5353	G	0.260	0.0824
5354	U	-	0.0000
5355	A	0.204	0.2262
5356	U	0.391	0.1477
5357	G	0.354	0.3499
5358	U	0.210	0.0518
5359	U	0.051	0.0242
5360	G	0.140	0.0908
5361	G	0.344	0.0662
5362	A	0.077	0.1398
5363	A	0.168	0.1357
5364	U	0.454	0.3787
5365	A	0.364	0.2819
5366	G	0.887	0.1391
5367	A	0.581	0.2825
5368	A	0.546	0.1591
5369	A	0.605	0.2091
5370	U	0.636	0.1624
5371	C	0.135	0.0964
5372	A	0.414	0.3159
5373	A	0.514	0.3451
5374	C	0.421	0.3241
5375	U	0.282	0.2514
5376	A	0.349	0.1836
5377	U	0.542	0.0912
5378	C	0.205	0.0000
5379	A	0.305	0.0000
5380	U	0.506	0.0000
5381	C	0.068	0.0000
5382	U	0.074	0.0000
5383	A	0.505	0.0000
5384	C	0.321	0.0000
5385	U	0.614	0.0000
5386	A	0.185	0.0000
5387	A	0.441	0.0000
5388	C	0.677	0.0000
5389	U	0.443	0.0000
5390	A	0.311	0.0000
5391	G	0.471	0.0000
5392	U	0.617	0.0000
5393	A	0.378	0.0000
5394	U	0.312	0.0000
5395	U	0.782	0.0000
5396	U	0.492	0.0000
5397	A	0.619	0.0000
5398	C	-	0.0000
5399	A	-	0.0000

5400	U	-	0.0000
5401	U	-	0.0000
5402	A	-	0.0000
5403	C	-	0.0000
5404	U	-	0.0000
5405	A	-	0.0000
5406	G	-	0.0000
5407	U	-	0.0000
5408	A	-	0.0000
5409	U	-	0.0000
5410	A	-	0.0000
5411	U	-	0.0000
5412	U	-	0.0000
5413	A	-	0.0000
5414	U	-	0.0000
5415	C	-	0.0000
5416	A	-	0.0000
5417	U	-	0.0000
5418	A	-	0.0000
5419	U	-	0.0000
5420	A	-	0.0000
5421	C	-	0.0000
5422	G	-	0.0000
5423	G	-	0.0000
5424	U	-	0.0000
5425	G	-	0.0000
5426	U	-	0.0000
5427	U	-	0.0000
5428	A	-	0.0000
5429	G	-	0.0000
5430	A	-	0.0000
5431	A	-	0.0000
5432	G	-	0.0000
5433	A	-	0.0000
5434	U	-	0.0000
5435	G	-	0.0000
5436	A	-	0.0000
5437	C	-	0.0000
5438	G	-	0.0000
5439	C	-	0.0000
5440	A	-	0.0000
5441	A	-	0.0000
5442	A	-	0.0000
5443	U	-	0.0000
5444	G	-	0.0000
5445	A	-	0.0000
5446	U	-	0.0000
5447	G	-	0.0000
5448	A	-	0.0000
5449	G	-	0.0000

5450	A	-	0.0000
5451	A	-	0.0000
5452	A	-	0.0000
5453	U	-	0.0000
5454	A	-	0.0000
5455	G	-	0.0000
5456	U	-	0.0000
5457	C	-	0.0000
5458	A	-	0.0000
5459	U	-	0.0000
5460	C	-	0.0000
5461	U	-	0.0000
5462	A	-	0.0000
5463	A	-	0.0000
5464	A	-	0.0000
5465	U	-	0.0000
5466	U	-	0.0000
5467	A	-	0.0000
5468	G	-	0.0000
5469	U	-	0.0000
5470	G	-	0.0000
5471	G	-	0.0000
5472	A	-	0.0000
5473	A	-	0.0000
5474	G	-	0.0000
5475	C	-	0.0000
5476	U	-	0.0000
5477	G	-	0.0000
5478	A	-	0.0000
5479	A	-	0.0000
5480	A	-	0.0000
5481	C	-	0.0000
5482	G	-	0.0000
5483	C	-	0.0000
5484	A	-	0.0000
5485	A	-	0.0000
5486	G	-	0.0000
5487	G	-	0.0000
5488	A	-	0.0000
5489	U	-	0.0000
5490	U	-	0.0000
5491	G	-	0.0000
5492	A	-	0.0000
5493	U	-	0.0000
5494	A	-	0.0000
5495	A	-	0.0000
5496	U	-	0.0000
5497	G	-	0.0000
5498	U	-	0.0000
5499	A	-	0.0000

5500	A	-	0.0000
5501	U	-	0.0000
5502	A	-	0.0000
5503	G	-	0.0000
5504	G	-	0.0000
5505	A	-	0.0000
5506	U	-	0.0000
5507	C	-	0.0000
5508	A	-	0.0000
5509	A	-	0.0000
5510	U	-	0.0000
5511	G	-	0.0000
5512	A	-	0.0000
5513	A	-	0.0000
5514	U	-	0.0000
5515	A	-	0.0000
5516	U	-	0.0000
5517	A	-	0.0000
5518	A	-	0.0000
5519	A	-	0.0000
5520	C	-	0.0000
5521	A	-	0.0000
5522	U	-	0.0000
5523	A	-	0.0000
5524	U	-	0.0000
5525	A	-	0.0000
5526	A	-	0.0000
5527	A	-	0.0000
5528	A	-	0.0000
5529	U	-	0.0000
5530	G	-	0.0000
5531	A	-	0.0000
5532	U	-	0.0000
5533	G	-	0.0000
5534	A	-	0.0000
5535	U	-	0.0000
5536	A	-	0.0000
5537	A	-	0.0000
5538	U	-	0.0000
5539	A	-	0.0000
5540	A	-	0.0000
5541	U	-	0.0000
5542	A	-	0.0000
5543	U	-	0.0000
5544	U	-	0.0000
5545	U	-	0.0000
5546	A	-	0.0000
5547	U	-	0.0000
5548	A	-	0.0000
5549	G	-	0.0000

5550	A	-	0.0000
5551	A	-	0.0000
5552	U	-	0.0000
5553	U	-	0.0000
5554	G	-	0.0000
5555	U	-	0.0000
5556	G	-	0.0000
5557	U	-	0.0000
5558	A	-	0.0000
5559	G	-	0.0000
5560	A	-	0.0000
5561	A	-	0.0000
5562	U	-	0.0000
5563	U	-	0.0000
5564	G	-	0.0000
5565	C	-	0.0000
5566	A	-	0.0000
5567	G	-	0.0000
5568	A	-	0.0000
5569	U	-	0.0000
5570	U	-	0.0000
5571	C	-	0.0000
5572	C	-	0.0000
5573	C	-	0.0000
5574	U	-	0.0000
5575	U	-	0.0000
5576	U	-	0.0000
5577	U	-	0.0000
5578	A	-	0.0000
5579	U	-	0.0000
5580	G	-	0.0000
5581	G	-	0.0000
5582	A	-	0.0000
5583	U	-	0.0000
5584	U	-	0.0000
5585	C	-	0.0000
5586	C	-	0.0000
5587	U	-	0.0000
5588	A	-	0.0000
5589	A	-	0.0000
5590	A	-	0.0000
5591	U	-	0.0000
5592	C	-	0.0000
5593	C	-	0.0000
5594	U	-	0.0000
5595	U	-	0.0000
5596	G	-	0.0000
5597	A	-	0.0000
5598	G	-	0.0000
5599	G	-	0.0000

5600	A	-	0.0000
5601	G	-	0.0000
5602	A	-	0.0000
5603	A	-	0.0000
5604	C	-	0.0000
5605	U	-	0.0000
5606	U	-	0.0000
5607	C	-	0.0000
5608	U	-	0.0000
5609	A	-	0.0000
5610	G	-	0.0000
5611	U	-	0.0000
5612	A	-	0.0000
5613	U	-	0.0000
5614	A	-	0.0000
5615	U	-	0.0000
5616	U	-	0.0000
5617	C	-	0.0000
5618	U	-	0.0000
5619	G	-	0.0000
5620	U	-	0.0000
5621	A	-	0.0000
5622	U	-	0.0000
5623	A	-	0.0000
5624	C	-	0.0000
5625	C	-	0.0000
5626	U	-	0.0000
5627	A	-	0.0000
5628	A	-	0.0000
5629	U	-	0.0000
5630	A	-	0.0000
5631	U	-	0.0000
5632	U	-	0.0000
5633	A	-	0.0000
5634	U	-	0.0000
5635	A	-	0.0000
5636	G	-	0.0000
5637	C	-	0.0000
5638	C	-	0.0000
5639	U	-	0.0000
5640	U	-	0.0000
5641	U	-	0.0000
5642	A	-	0.0000
5643	U	-	0.0000
5644	C	-	0.0000
5645	A	-	0.0000
5646	A	-	0.0000
5647	C	-	0.0000
5648	A	-	0.0000
5649	A	-	0.0000

5650	U	-	0.0000
5651	G	-	0.0000
