# Supplementary material for: Cell Compartment-Specific Folding of Ty1 Long Terminal Repeat Retrotransposon RNA Genome
Source: Viruses. 2022 Sep 10;14(9):2007. doi: 10.3390/v14092007 (PMC9503155; doi:10.3390/v14092007)
Supplement: Supplementary file 1 [file viruses-14-02007-s001.zip › Supplemetary_File_Zawadzka_et_al.pdf]

SUPPLEMENTARY TABLES AND FIGURES

Cell compartment-specific folding of Ty1 retrotransposon RNA genome

Małgorzata Zawadzka[1], Angelika Andrzejewska-Romanowska[1], Julita Gumna[1], David J. Garfinkel[2] and Katarzyna Pachulska-Wieczorek[1,*]

Table S1 . Reverse transcription primers.

PRIMER	SEQUENCE (5'-3')
Ty1 PR_1	TCAGGTGATGGAGTGCTCAG
Ty1 PR_2	TCGTTTGCCTCTTGGGTATC
Ty1 PR_3	CATCACTCGGATTTCTCCTG
Ty1 PR_4	TTGGTGTTGTCCTGGAAGTGA
Ty1 PR_5	TCGAATTGTCTGTGCATTGG
Ty1 PR_6	AGACACTGGCCTGAAACTGG
Ty1 PR_7	GCCACGAGGATGTATTTTGG
Ty1 PR_8	GGAGGTGTGGAATCGGTTGG
Ty1 PR_9	GGTACGTTTGTATGATTAGTCTCA
Ty1 PR_10	GTCTCGTGATACCTTAATTTCAG
Ty1 PR_11	TGCTGAATATCACCTCTTGC
Ty1 PR_12	CAAATTGTCACTTGACTG
Ty1 PR_13	TGCATTTCATGTACCTTCTC
Ty1 PR_14	GCTTCCGTAGTTGAAGTACAT
Ty1 PR_15	TCTATTCCAACATACCACCC
Ty1 PR_16	CTCCTCAAGGATTTAGGAATCC
Ty1 PR_17	CATTGTTGATAAAGGCTATA
U1 PR	TAAGATCCACCCGTTCTAC

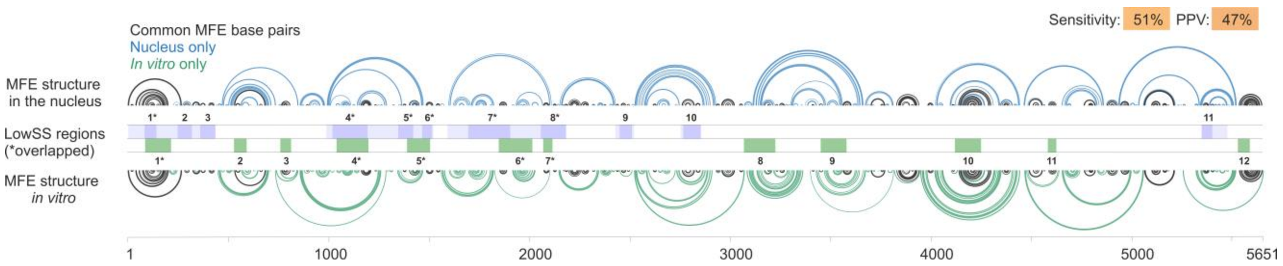

Figure S1. Comparison of SHAPE-directed MFE model predicted for nuclear and Ty1 gRNA *in vitro*. Location of lowSS regions identified for nuclear and in vitro Ty1 gRNA are presented. Overlapped regions are marked by asterix *.
